# Prevalence of statin intolerance: a meta-analysis^[Author-notes ehac015-FM1]^

**DOI:** 10.1093/eurheartj/ehac015

**Published:** 2022-02-16

**Authors:** Ibadete Bytyçi, Peter E Penson, Dimitri P Mikhailidis, Nathan D Wong, Adrian V Hernandez, Amirhossein Sahebkar, Paul D Thompson, Mohsen Mazidi, Jacek Rysz, Daniel Pella, Željko Reiner, Peter P Toth, Maciej Banach

**Affiliations:** Department of Public Health and Clinical Medicine, Umeå University, Umeå, Sweden; Clinic of Cardiology, University Clinical Centre of Kosovo, Prishtina, Kosovo; School of Pharmacy and Biomolecular Sciences, Liverpool John Moores University, Liverpool, UK; Liverpool Centre for Cardiovascular Science, Liverpool, UK; Department of Clinical Biochemistry, Royal Free Hospital Campus, University College London Medical School, University College London (UCL), London, UK; Heart Disease Prevention Program, Division of Cardiology, University of California, Irvine School of Medicine Predictive Health Diagnostics, Irvine, CA, USA; Health Outcomes, Policy, and Evidence Synthesis (HOPES) Group, University of Connecticut School of Pharmacy, Storrs, CT, USA; Vicerrectorado de Investigación, Universidad San Ignacio de Loyola (USIL), Lima, Peru; Biotechnology Research Center, Pharmaceutical Technology Institute, Mashhad University of Medical Sciences, Mashhad, Iran; Applied Biomedical Research Center, Mashhad University of Medical Sciences, Mashhad, Iran; School of Pharmacy, Mashhad University of Medical Sciences, Mashhad, Iran; Division of Cardiology, Hartford Hospital, 80 Seymour Street, Hartford, CT, USA; Department of Internal Medicine, University of Connecticut, Farmington, CT, USA; Department of Twin Research and Genetic Epidemiology, King’s College London, London, UK; Department of Nutritional Sciences, King’s College London, London, UK; Department of Hypertension, Nephrology and Family Medicine, Medical University of Lodz (MUL), Lodz, Poland; 2nd Department of Cardiology, Faculty of Medicine, Pavol Jozef Safarik University and East Slovak Institute of Cardiovascular Diseases, Kosice, Slovakia; Department of Internal Diseases, University Hospital Center Zagreb, School of Medicine, Zagreb University, Zagreb, Croatia; CGH Medical Center, Sterling, IL, USA; Cicarrone Center for the Prevention of Cardiovascular Disease, Johns Hopkins University School of Medicine, Baltimore, MD, USA; Department of Preventive Cardiology and Lipidology, Medical University of Lodz (MUL), Rzgowska 281/289, 93-338 Lodz, Poland; Cardiovascular Research Centre, University of Zielona Gora, Zielona Gora, Poland

**Keywords:** Cardiovascular disease, Prevalence, Risk factors, Statin intolerance

## Abstract

**Aims:**

Statin intolerance (SI) represents a significant public health problem for which precise estimates of prevalence are needed. Statin intolerance remains an important clinical challenge, and it is associated with an increased risk of cardiovascular events. This meta-analysis estimates the overall prevalence of SI, the prevalence according to different diagnostic criteria and in different disease settings, and identifies possible risk factors/conditions that might increase the risk of SI.

**Methods and results:**

We searched several databases up to 31 May 2021, for studies that reported the prevalence of SI. The primary endpoint was overall prevalence and prevalence according to a range of diagnostic criteria [National Lipid Association (NLA), International Lipid Expert Panel (ILEP), and European Atherosclerosis Society (EAS)] and in different disease settings. The secondary endpoint was to identify possible risk factors for SI. A random-effects model was applied to estimate the overall pooled prevalence. A total of 176 studies [112 randomized controlled trials (RCTs); 64 cohort studies] with 4 143 517 patients were ultimately included in the analysis. The overall prevalence of SI was 9.1% (95% confidence interval 8.0–10%). The prevalence was similar when defined using NLA, ILEP, and EAS criteria [7.0% (6.0–8.0%), 6.7% (5.0–8.0%), 5.9% (4.0–7.0%), respectively]. The prevalence of SI in RCTs was significantly lower compared with cohort studies [4.9% (4.0–6.0%) vs. 17% (14–19%)]. The prevalence of SI in studies including both primary and secondary prevention patients was much higher than when primary or secondary prevention patients were analysed separately [18% (14–21%), 8.2% (6.0–10%), 9.1% (6.0–11%), respectively]. Statin lipid solubility did not affect the prevalence of SI [4.0% (2.0–5.0%) vs. 5.0% (4.0–6.0%)]. Age [odds ratio (OR) 1.33, *P* = 0.04], female gender (OR 1.47, *P* = 0.007), Asian and Black race (*P* < 0.05 for both), obesity (OR 1.30, *P* = 0.02), diabetes mellitus (OR 1.26, *P* = 0.02), hypothyroidism (OR 1.37, *P* = 0.01), chronic liver, and renal failure (*P* < 0.05 for both) were significantly associated with SI in the meta-regression model. Antiarrhythmic agents, calcium channel blockers, alcohol use, and increased statin dose were also associated with a higher risk of SI.

**Conclusion:**

Based on the present analysis of >4 million patients, the prevalence of SI is low when diagnosed according to international definitions. These results support the concept that the prevalence of complete SI might often be overestimated and highlight the need for the careful assessment of patients with potential symptoms related to SI.

## Structured Graphical Abstract

**Structured Graphical Abstract:**
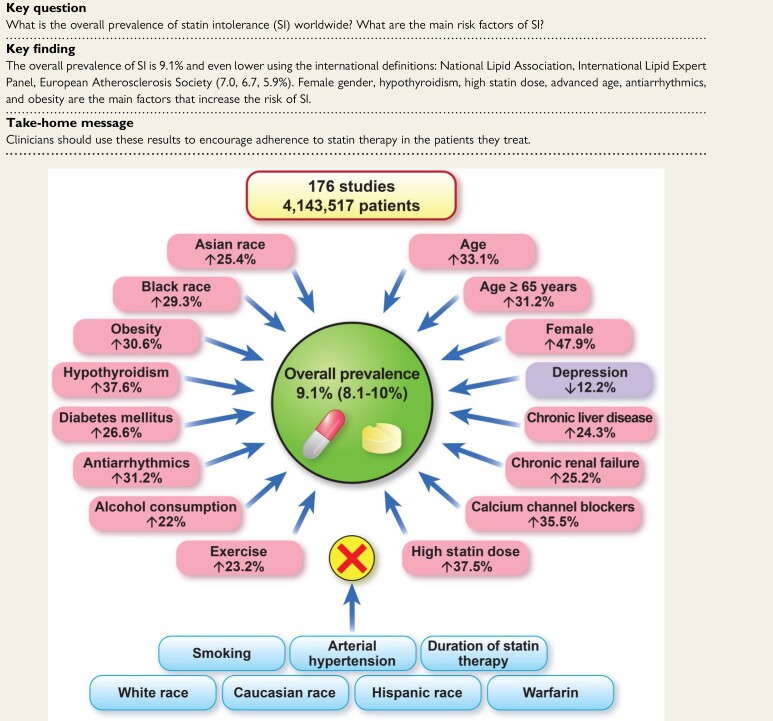
The worldwide prevalence of statin intolerance and risk factors/conditions that effect or do not effect the risk of statin intolerance.

**See the editorial comment for this article ‘Statin intolerance: how common is it and how do we work with patients to overcome it?’, by Christopher P. Cannon, https://doi.org/10.1093/eurheartj/ehac156.**

## Introduction

Cardiovascular (CV) disease (CVD) is the leading cause of morbidity and mortality worldwide, despite continuous improvement of medical treatment, diagnosis, and risk factor control.^1^ It has been clearly demonstrated that statin therapy confers significant mortality and morbidity benefits in both the primary and secondary prevention of CVD.^2^ Although statins are among the most commonly prescribed drugs, non-adherence and discontinuation of statin therapy is an ongoing problem worldwide.^3^ The most common cause of discontinuation of statin therapy is statin-associated muscle symptoms (SAMS).^4,5^ Other possible statin-related adverse effects include neurocognitive disorders, hepatotoxicity, haemorrhagic stroke, and renal toxicity.^6,7^ These conditions may lead to discontinuation, but causality has been confirmed only for SAMS, temporary elevation of aminotransferase alanine, and newly diagnosed diabetes.^6^ According to the International Lipid Expert Panel (ILEP), statin intolerance (SI) is an inability to tolerate a dose of statin required to sufficiently reduce an individual’s CV risk, limiting the effective treatment of patients at risk of, or with, CVD.^7^ The National Lipid Association (NLA) has a wider definition, including any adverse effects relating to the quality of life and leading to the decision to decrease or stop the use of an otherwise beneficial drug.^8^ The Luso-Latin American Consortium (LLAC) definition of SI is similar to that of the Canadian Consensus Working Group (CCWG). It refers to an inability to tolerate ≥2 statins at any dose or an inability to tolerate increasing doses. The symptoms must not be attributable to drug–drug interactions or conditions known to increase SI.^9,10^ They indicate that symptomatic criteria include intolerable muscle symptoms [pain, weakness, or cramps with or without creatine kinase (CK) changes] or severe myopathy, and they must appear in the first 12 weeks after initiating treatment or following an increase in dose.^9,10^

The prevalence of SI is widely debated, in part because of difficulties in identification and diagnosis, possible interaction of different risk factors, different diseases, drugs, and other clinical and demographic indices.^11^ In contrast with randomized controlled trials (RCTs) (prevalence usually 5–7%), cohort studies suggest that SI occurs in as many as 30% of treated patients.^8,12^ However, this is likely to be an overestimate or underestimate and in many cases, the symptoms are likely to be attributable to the nocebo/drucebo effect.^11^

Because of these inconsistent findings, the present meta-analysis aimed to estimate the overall prevalence of SI, its prevalence according to various diagnostic criteria, in different disease settings, and to identify possible risk factors for SI.

## Methods

### Search strategy and selection criteria

We followed the methods recommended by the Cochrane Collaboration and complied with the reporting standards of the Preferred Reporting Items for Systematic Review and Meta-analysis (PRISMA) guideline of 2020.^13^ A PECOS (population, exposure, comparison, outcomes, study design) model was used to shape the clinical question and to design the search strategy (see Supplementary material online, *Table S1*). The following databases were searched from inception through 31 May 2021: PubMed-Medline, EMBASE, Scopus, Google Scholar, the Cochrane Central Registry of Controlled Trials, and ClinicalTrial.gov. The following keywords were used: statin intolerance, statin toxicity, statin adverse effects, statin side effects, statin-associated muscle symptoms, SAMS, statin-related myopathy, statin-related side effects, statin-related myalgia, statin discontinuation, statin withdrawal, prevalence, occurrence rate, and frequency rate (see Supplementary material online, *Table S2*). In addition, the references from the selected articles and relevant review articles, and the abstracts from selected congresses: scientific sessions of the European Society of Cardiology, the American Heart Association (AHA), American College of Cardiology (ACC), NLA, and European Atherosclerosis Society (EAS) were screened for additional relevant articles. The wild-card term ‘*’ was used to increase the sensitivity of the search strategy.

Articles were eligible if they reported the prevalence of SI either in primary or secondary prevention and met the following inclusion criteria: (i) trials or cohorts reporting SI, (ii) at least 100 participants included in the analysis, and (iii) available criteria for SI diagnosis. Exclusion criteria were as follows: (i) studies with unclear methodologies to obtain the estimates of SI frequency, (ii) studies that investigated a statin that has been withdrawn from the market, (iii) ongoing trials (unless they reported relevant interim results), (iv) studies only investigating statin discontinuation without specifying intolerance, and (v) short follow-up (<1.5 month/6 weeks).

The search, screening, and data extraction were performed independently by two reviewers (I.B. and J.R.); any disagreements were resolved through discussion with senior investigators (M.B. and P.E.P.). Non-relevant articles were excluded on the basis of title and abstract screening. For each trial, the risk of bias was independently assessed by the same investigators using the revised Cochrane RoB2 tool involving five domains (randomization process, deviation from intended interventions, missing outcome data, outcome measurement, and selection of reported results). The risk of bias in each study was judged to be ‘low’, ‘high’, or ‘unclear’.^14^ For the assessment of the risk of bias in cohort studies, the Newcastle-Ottawa Scale (NOS) was used. Three domains were evaluated with the following items: (i) selection, (ii) comparability, and (iii) exposure. The risk of bias in each study was judged to be ‘good’, ‘fair’, or ‘poor’.^15^

### Outcome measures

The primary endpoint was the overall prevalence and the prevalence based on each of the international diagnostic criteria: NLA, EAS, and ILEP. The secondary endpoint was the prevalence of SI in groups of patients with different diseases and the analysis of the association between possible risk factors/conditions and the risk of SI. According to the NLA, SI is defined as adverse effects relating to the quality of life, leading to decisions to decrease or stop the use of an otherwise beneficial drug.^8^ The ILEP definition stated that SI is an inability to tolerate a dose of statin required to reduce a person’s CV risk sufficiently from their baseline risk and could result from different statin-related side effects.^7^ The EAS definition focused only on SAMS: the assessment of the probability of SAMS being due to a statin considering the nature of the muscle symptoms, the elevation in CK levels, and their temporal association with statin initiation, discontinuation, and re-challenge.^16^ As stated by the CCWG and LLAC, SI was defined as a clinical syndrome characterized by significant symptoms and biomarker abnormalities that is documented by challenge/dechallenge/re-challenge using ≥2 statins that is not due to drug interactions or untreated risk factors for intolerance^9,10^ (see Supplementary material online, *Figure S1*). Because the main outcome was not limited by the type of statin, the CCWG and LLAC criteria were not used in further analyses.

### Data synthesis and statistical analyses

The meta-analysis was conducted using R Statistical Software (v3.5.1, Boston, MA, USA), using the packages ‘meta’ and ‘metafor’ for meta-analysis. A random-effects model (DerSimonian and Laird method) was applied to estimate the pooled prevalence across the studies. The 95% confidence intervals (CIs) for the prevalence reported in the individual studies (see Supplementary material online, *Table S1*) were estimated from the proportion of cases of SI and sample size using the binomial exact method (Clopper–Pearson method). An inverse variance method was used for weighting each study in the meta-analysis. For the difference of subgroup analysis, we employed *post hoc* analysis. To investigate the differences between groups, we used the significance test. An *I*^2^ statistic was also computed for subgroup differences.^14^ With the inverse variance method, when the estimated probability of the condition of a single study approaches 0 or 1, the variance of the study approaches zero, which in turn causes the inverse variance to approach infinity; subsequently, the inflated inverse variance substantially increases the adjusted weight of the study in the pooled mean, resulting in an over-contribution of the study in the final pooled estimation of the meta-analysis. Therefore, to avoid the overestimated results, we conducted the Freeman–Tukey double arcsine. The final pooled result and 95% CIs were then back-transformed and expressed as percentages for ease of interpretation. The baseline characteristics are reported as the median and range. The mean and standard deviation values were estimated using the method described by Hozo *et al*.^16^ Heterogeneity between studies was assessed using Cochrane’s *Q*-test and the *I*^2^ index. As a guide, *I*^2^ < 25% indicated low, 25–50% moderate, and >50% high heterogeneity.^17^

Potential demographic, clinical, and drugs as modifiers of SI were further explored by meta-regression. Meta-regression coefficients and corresponding *P*-values are reported. For summary estimates, *P* < 0.05 (two-tailed) was considered statistically significant.^18^

## Results

### Study selection and patient population

**Figure 1:**
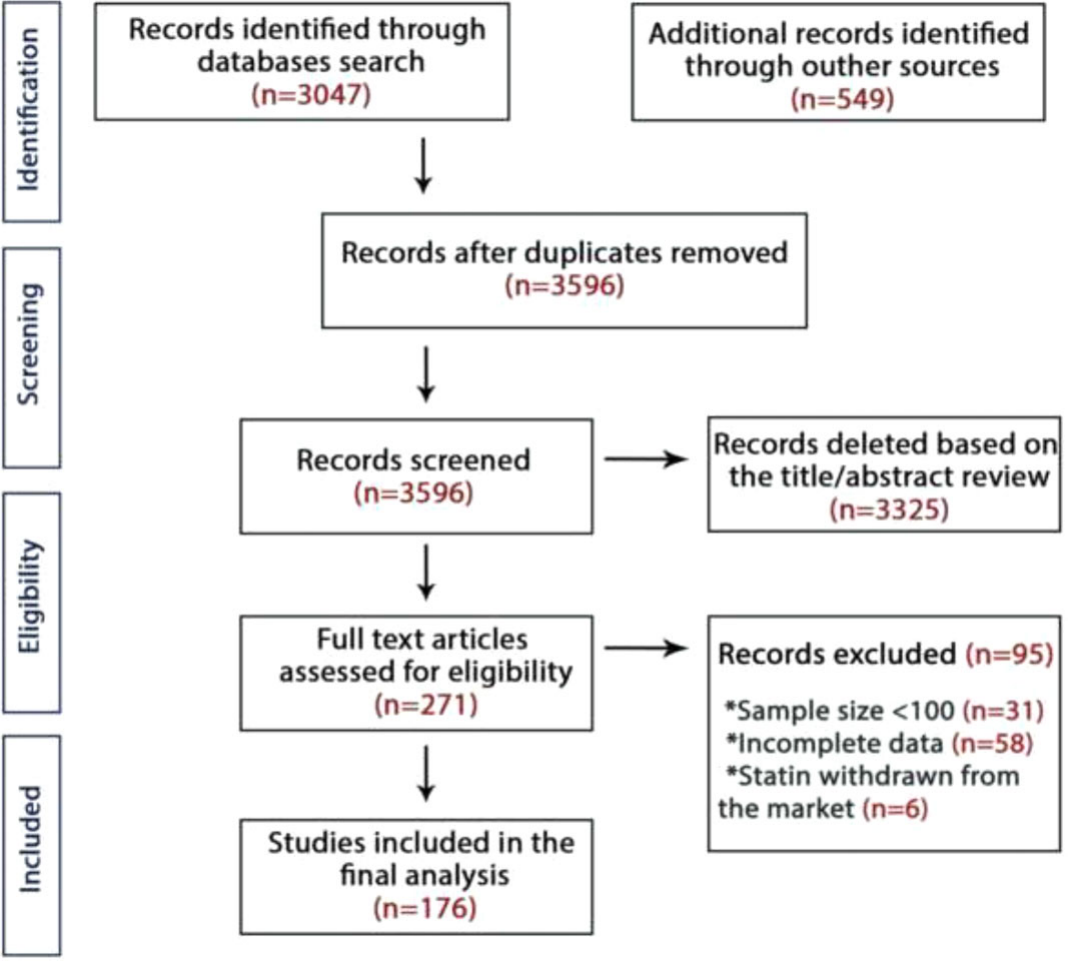
The Preferred Reporting Items for Systematic Review and Meta-analysis flow-chart of studies included in the meta-analysis. *Incomplete data:* Studies that reported only statin discontinuation without specifying the reasons for discontinuation.

A total of 3569 articles were retrieved from the search after duplicates from the different databases were discarded. These articles were first screened by title and abstract, leading to 271 articles that underwent full-text review. After a stringent selection process, a total of 176 studies with 4 143 517 patients and a mean follow-up of 19 ± 7.3 months were included in the analysis.^19–194^ Out of 176 articles, 112 were RCTs (195 575 patients) and the remaining 64 were cohort studies with 3 947 942 patients. The PRISMA flow diagram is shown in *Figure 1* and the key characteristics of the included studies are presented in Supplementary material online, *Table S3*. The mean age of patients was 60.5 ± 8.9 and 40.9% were females. The White or Caucasian race made up a greater proportion of participants than Afro-American, Asian, Hispanic, or others (81.1, 8.25.1, 4.5, and 1.2%, respectively; *P* < 0.001; *Table 1*).

**Table 1.** Summary of main characteristics of studies included in the present meta-analysis

	All studies	RCT studies	Cohort studies	Primary prevention	Secondary prevention	Combined patients^a^
**No. of studies**	176	112	64	93	54	29
**Overall prevalence, % (95% CI)**	9.1 (8.0–10)	4.9 (4.0–6.0)	17 (14–19)	8.2 (6.0–10)	9.1 (6.0–11)	18 (14–21)
NLA	7.0 (6.0–8.0)	4.8 (3.0–6.0)	11 (6.0–16)	7.5 (5.0–9.8)	8.7 (5.9–11)	16 (11–19)
ILEP	6.7 (5.0–8.0)	4.9 (3.5–6.2)	10 (7.2–15)	7.3 (5.0–9.1)	8.1 (6.0–11)	15.3 (10–18)
EAS	5.9 (4.0–7.0)	3.8 (2.4–5.4)	8.4 (5.7–11)	6.2 (4.8–8.9)	5.5 (4.0–9.1)	12 (9.1–17)
**Sample size, *n***	4 143 517	195 575	3 947 942	1 726 384	1 166 745	1 250 388
**Female sex, %**	40.9	38.6	43.1	44.4	31.2	47.3
**Age, years, mean ± SD**	60.54 ± 8.88	59.2 ± 8.12	61.9 ± 7.89	58.3 ± 7.12	62.9 ± 9.1	62.9 ± 9.1
**Race, %**						
White or Caucasian	81.1	78	80	81	85	82
Black	8.2	6.2	11	7.5	3.3	11.9
Asian	5.1	6.1	3.2	6.2	2.9	5.1
Hispanic	4.5	8	5.4	4.9	4.5	0.6
Other	1.2	1.7	0.3	0.4	0.3	0.4

CI, confidence interval; NLA, National Lipid Association; ILEP, International Lipid Expert Panel; EAS, European Atherosclerosis Society; RCT, randomized controlled trial; SD, standard deviation.

a Combined: primary and secondary prevention patients.

### Prevalence of statin intolerance

The pooled prevalence of SI was 9.1% (95% CI 8.0–10%, see Supplementary material online, *Figure S2*). The prevalence based on NLA criteria was similar compared with using the ILEP or EAS definitions [7.0% (6.0–8.0%), *I*^2^ = 98%; 6.7% (5.0–8.0%), *I*^2^ = 98%; 5.9% (4.0–7.0%), *I*^2^ = 93%, respectively; see Supplementary material online, *Figures S3–S5*]. The prevalence of SI in RCTs was significantly lower compared with cohort studies [4.9% (4.0–6.0%), *I*^2^ = 93% vs. 17% (14–19%), *I*^2^ = 98%; *P* < 0.001, see Supplementary material online, *Figures S6* and *S7*].

**Figure 2:**
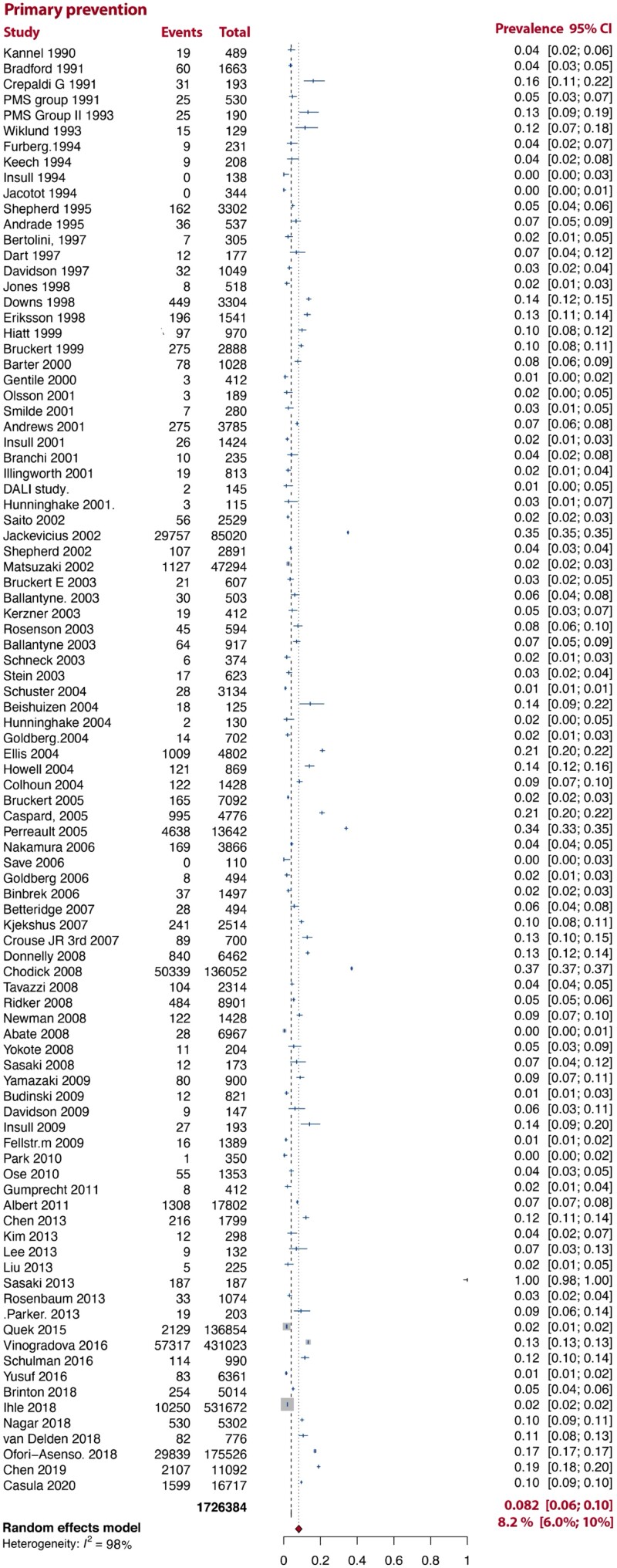
Prevalence of statin intolerance in primary prevention studies. *Note:* D–L random-effects model was used.

**Figure 3:**
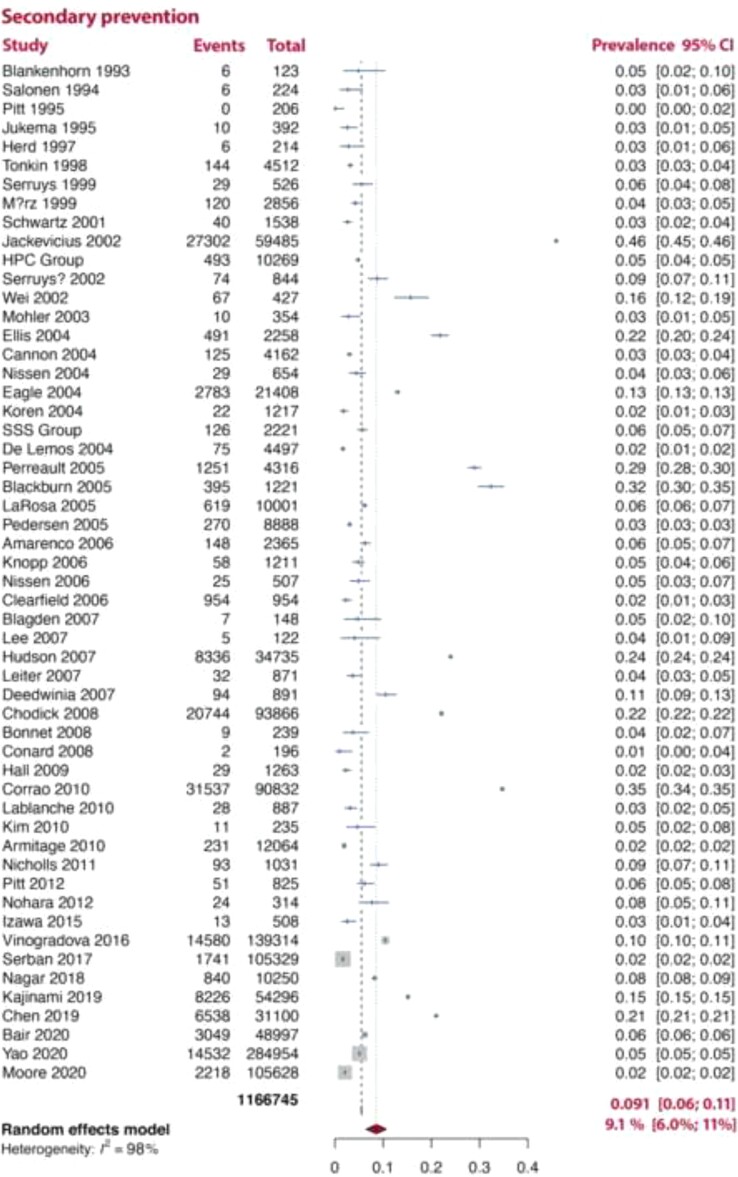
Prevalence of statin intolerance in secondary prevention studies. *Note:* D–L random-effects model was used.

**Figure 4:**
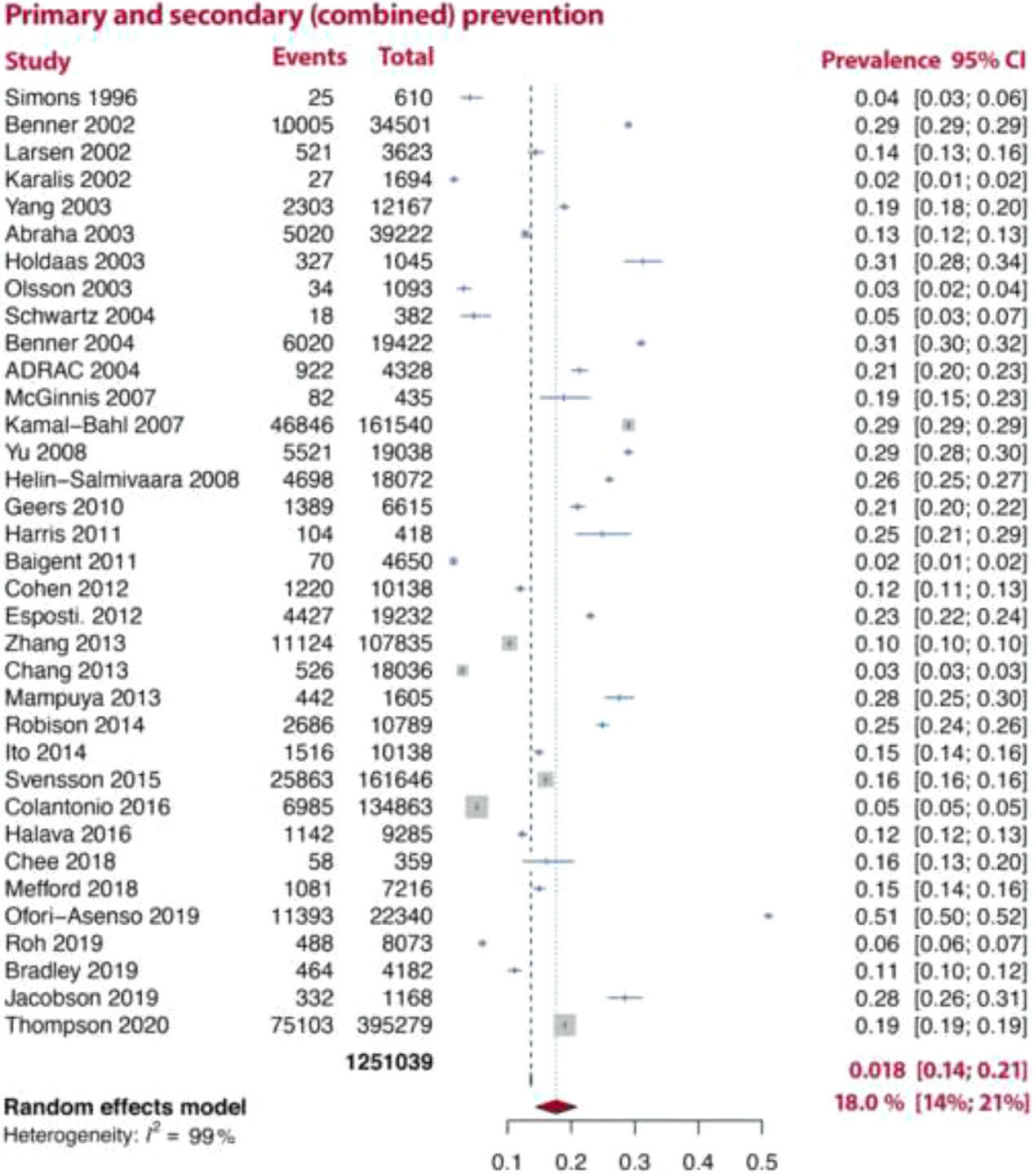
Prevalence of statin intolerance in combined primary and secondary prevention studies. *Note:* D–L random-effects model was used.

In an analysis stratified by the type of disease prevention, SI was more common in pooled analyses of studies which included both primary and secondary prevention [18% (14–21%), *I*^2^ = 99%] patients than in either pooled analyses of studies which only included primary or secondary prevention patients [8.2% (6.0–10%, *I*^2^ = 98%), 9.1% (6.0–11%, *I*^2^ = 98%), respectively; *Figures 2–4*].

In the subgroup analysis according to disease states, in primary prevention patients with familial hypercholesterolaemia (FH), hypercholesterolaemia, dyslipidaemia, and Type 2 diabetes mellitus (T2DM), the prevalence of SI was 9.0% (6.0–13%, *I*^2^ = 96%), 12% (11–13%, *I*^2^ = 99%), 13% (7.0–18%, *I*^2^ = 98%), and 6.0% (2.0–10%, *I*^2^ = 99%) (see Supplementary material online, *Figure S8*), respectively. In secondary prevention: stable coronary artery disease (CAD), acute coronary syndrome (ACS), myocardial infarction (MI), and stroke/transient ischaemic attack were associated with SI prevalence of 8% (2.0–18%, *I*^2^ = 98%), 13% (2.0–24%, *I*^2^ = 98%), 13% (2.0–24%, *I*^2^ = 98%), and 5.4% (3.9–9.1%, *I*^2^ = 96%), respectively (see Supplementary material online, *Figure S9*).

**Figure 5:**
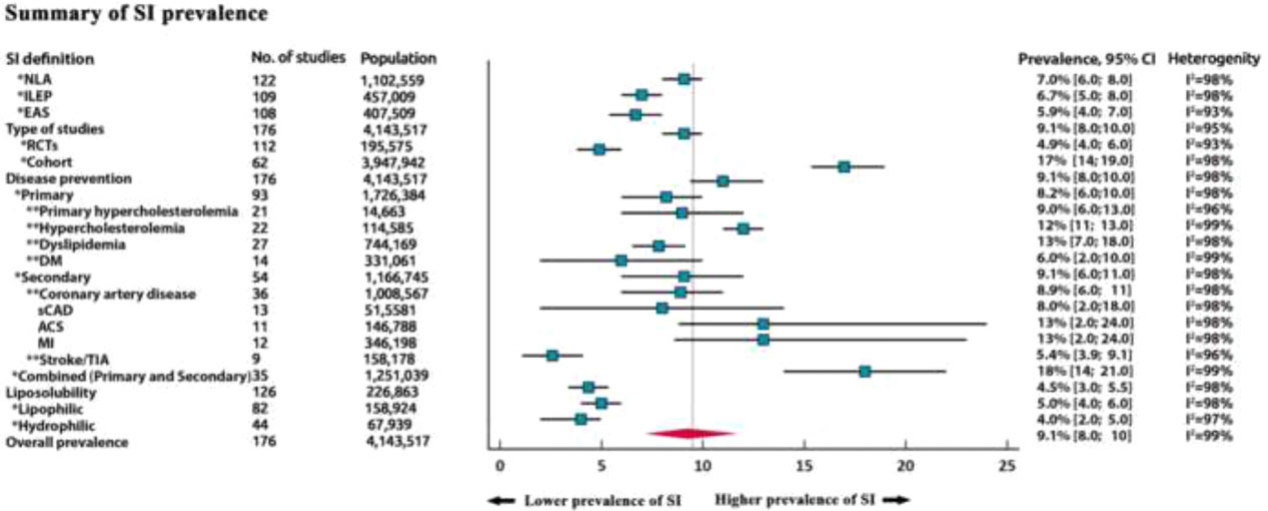
Prevalence of statin intolerance—summary figure. NLA, National Lipid Association; ILEP, International Lipid Expert Panel; EAS, European Atherosclerosis Society; RCTs, randomized controlled trials; DM, diabetes mellitus; sCAD, stable coronary artery disease; ASC, acute coronary syndrome; MI, myocardial infarction; TIA, transient ischaemic attack; SI, statin intolerance.

We also compared the prevalence of SI in patients treated with lipophilic (atorvastatin, simvastatin, lovastatin, fluvastatin, and pitavastatin) and hydrophilic statins (pravastatin and rosuvastatin). The pooled prevalence was similar in these two types [4.0% (2.0–5.0%, *I*^2^ = 97%) vs. 5.0% (4.0–6.0%, *I*^2^ = 98%), respectively; *P* = 0.33, see Supplementary material online, *Figures S10* and *S11*]. A summary of SI prevalence is shown in *Figure 5*. Between-study heterogeneity was large (*I*^2^ ≥ 93%). Tests assessing bias were non-significant (*P* > 0.28).

### Interaction of demographic indices with statin intolerance

**Figure 6:**
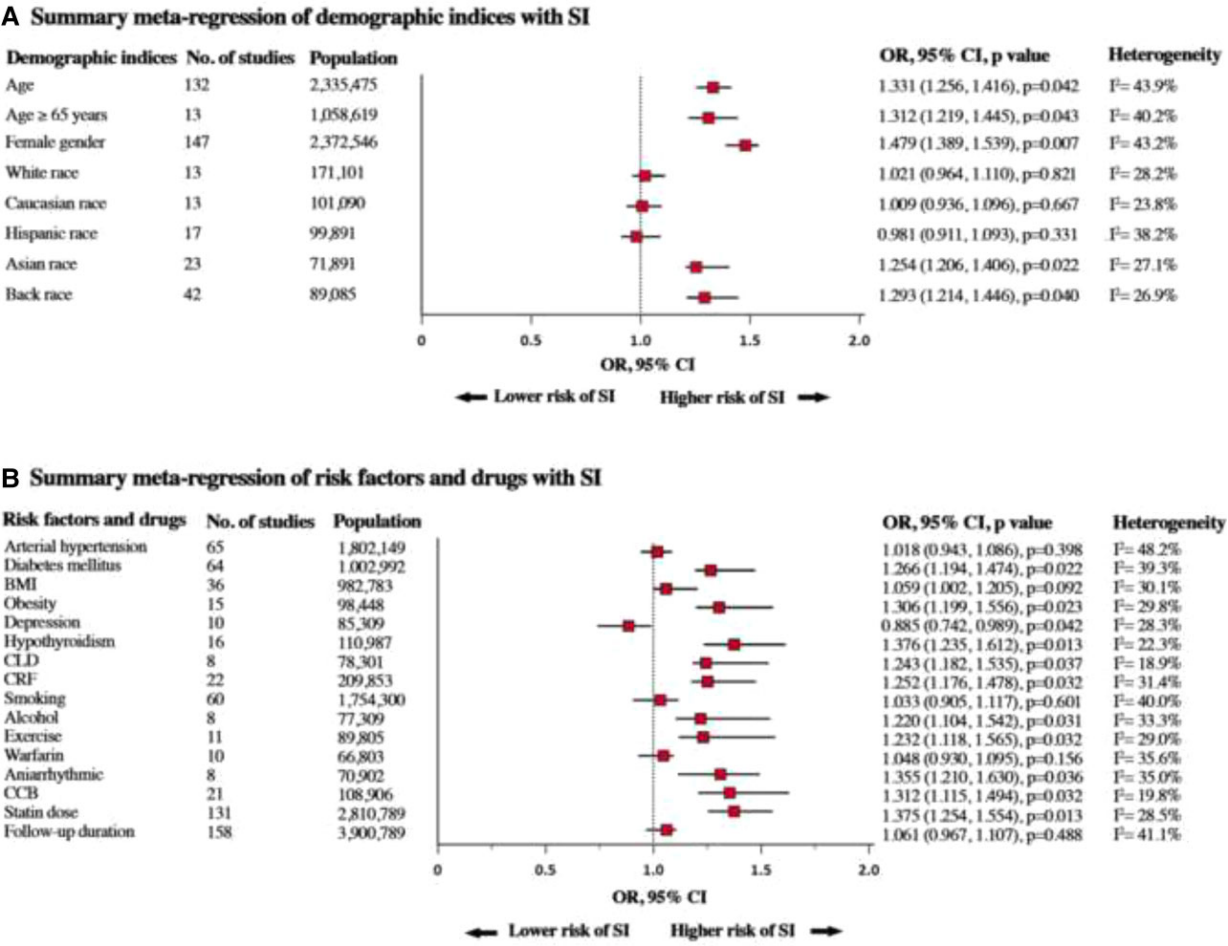
Summary meta-regression of (*A*) demographic and (*B*) risk factors and drugs with statin intolerance. SI, statin intolerance; BMI, body mass index; CLD, chronic liver disease; CRF, chronic renal failure; CCB, calcium channel blockers.

In meta-regression analyses, age (as a continuous variable) was found to be significantly associated with the higher risk for SI [odds ratio (OR) 1.33, 95% CI 1.25–1.41; *P* = 0.04, see Supplementary material online, *Figure S12A*]. Likewise, the older age ≥65 years (OR 1.31, 95% CI 1.22–1.45; *P* = 0.04, see Supplementary material online, *Figure S12B*) and female sex were associated with a higher risk of SI (OR 1.47, 95% CI 1.38–1.53; *P* = 0.007) (see Supplementary material online, *Figure S12C*). Analysis of demographic indices revealed that the prevalence of SI was associated with the percentage of participants of Asian and African-American race (*P* < 0.05 for both, see Supplementary material online, *Figure S12G* and *H*). However, no association was observed with White, Caucasian, and Hispanic races with SI (*P* > 0.05 for all, see Supplementary material online, *Figure S12D–F*). A summary of the meta-regression of demographic indices on SI is shown in *Figure 6*A.

### Interaction of clinical indices with statin intolerance

A range of potential factors was tested for possible interaction with SI. Positive associations were found for obesity (OR 1.30, *P* = 0.02), diabetes mellitus (OR 1.26, *P* = 0.02), hypothyroidism (OR 1.37, *P* = 0.01), chronic liver disease (OR 1.24, *P* = 0.03), and chronic renal failure (OR 1.25, *P* = 0.03), whereas the percentage of individuals with depression was found to have a negative association with SI (OR 0.88, *P* = 0.04). Conversely, arterial hypertension was not associated with the prevalence of SI (see Supplementary material online, *Figure S13*).

### Interaction of drugs and addiction diseases with statin intolerance

The percentage of smokers was not significantly associated with the prevalence of SI (OR 1.03, *P* = 0.60), whereas the percentage of alcohol users used showed a significant association with the prevalence of SI (OR 1.22, *P* = 0.03). Moreover, exercise (OR 1.23, *P* = 0.03), calcium channel blockers (CCB) (OR 1.31, *P* = 0.03), and antiarrhythmic agents (OR 1.35, *P* = 0.03) were associated with higher risk of SI, whereas warfarin use was not (OR 1.04, *P* = 0.15). In addition, increased statin dose was associated with a higher prevalence of SI (OR 1.37, *P* = 0.01), whereas the duration of study follow-up was not associated with the occurrence of SI (OR 1.06, *P* = 0.48, see Supplementary material online, *Figure S14*). A summary of the results of meta-regression with respect to associations between risk factors and drugs on SI is shown in *Figure 6*B.

### Risk of bias assessment

The assessment of the risk of bias in the included studies using RoB2 for RCTs and NOS for cohort studies showed that most studies had moderate to high-quality level in defining objectives and the main outcomes (see Supplementary material online, *Tables S4* and *S5*).

## Discussion

To the best of our knowledge, the present meta-analysis is the first to evaluate the overall prevalence of SI worldwide, the prevalence based on different diagnostic criteria and in different disease settings. The results of our meta-analysis of 176 studies with 4 143 517 patients and a mean follow-up of 19 ± 7.3 months showed that the worldwide prevalence of SI is 9.1%, irrespective of the definition applied. Older age, female gender, Asian and African-American races, obesity, T2DM, alcohol use, hypothyroidism, chronic liver, and renal diseases were associated with a higher risk of SI, as were increased statin doses and the concomitant administration of antiarrhythmic agents (*Structured Graphical abstract*).

Statin intolerance and the discontinuation of statin therapy is an ongoing clinical problem worldwide.^1–3^ Statin intolerance is associated with suboptimal lipid-lowering therapy and a high risk of first and recurrent CVD events.^176^ Numerous studies, systematic reviews, and meta-analyses have demonstrated an association between statin non-adherence and discontinuation and the risk of CVD and mortality.^195,196^

Although a wide range of values for the prevalence of SI has been reported in the literature (from 2 to 3% to as high as 50%),^3,11,91,117^ our findings show that the pooled overall worldwide prevalence ranges from 8.1 to 10% (1 in every 10–12 patients). There is debate on the definition of SI. We compared the prevalence of SI according to all major definitions. Despite the fact that the EAS definition of SI is focused solely on SAMS, the pooled prevalence in our analysis did not show significant differences between the EAS, NLA, and ILEP definitions.

The prevalence of SI in cohort studies was significantly higher than that reported in RCTs. This is associated with large difficulties of correct SI diagnosis in clinical practice and lack of possibility of using of new one-of-trial approach or even cross-over design as it was applied, e.g. in PCSK9 inhibitors trials.^197–199^ This also suggests that the prevalence of SI is overestimated in real-life data. It is also possible that RCTs underestimate the prevalence by excluding older patients and those with comorbidities such as chronic liver and kidney diseases and abnormal laboratory values that may increase the risk of SI. Some previous studies have reported substantially lower adherence rates in primary prevention compared with patients with CVD or after MI.^61,87,100,200^ In contrast, our sub-analysis of the pooled prevalence of SI in primary prevention (93 papers with 1 762 384 participants) and secondary prevention (54 papers with 1 166 745 participants) did not find a significant difference (8.2 vs. 9.1%). However, in observational cohort studies which included mixed patients (both primary and secondary prevention), the pooled prevalence of SI was twice as high (18%). This finding suggests that such studies overestimate the prevalence of SI. Similarly, in the subgroup analysis based on different diseases in the primary prevention cohorts (FH, hypercholesterolaemia, dyslipidaemia, and T2DM) and secondary prevention (stable CAD, ACS, and MI), the mean overall SI prevalence was not significantly different. Likewise, regarding the safety of different classes of statins, we found no difference between lipophilic and hydrophilic statins.

Because statins are the gold standard for the treatment of dyslipidaemia and in the management of elevated CV risk, the most important issue during the diagnosis and management of patients with SI is the urgent need to continue statin therapy. To predict the risk of SI and to be effective in lipid management, it is critically important to know the risk factors and conditions that might increase the risk of SI.^4^ It is now 20 years since the ACC/AHA/National Heart Lung and Blood Institute first identified risk factors in their recommendations for statin safety; however, there has been no attempt to validate their suggested risk factors using data from clinical trials or observational studies.^201^ In this meta-analysis, we have attempted to investigate what risk factors/conditions might be linked to SI prevalence using meta-regression. Pooled analysis demonstrated that many demographic, clinical, and other risk factors are associated with SI. Older age, female gender, Asian, and African-American races were associated with a higher incidence of SI, whereas White, Caucasian, and Hispanic races were not associated with higher SI risk. Many commonly observed risk factors and conditions may also be significantly associated with SI occurrence, including obesity, diabetes mellitus, hypothyroidism, chronic liver disease, and renal failure. Depression was negatively associated with SAMS, perhaps because of under-reporting in these patients.^202–205^ Smoking and anticoagulant drugs were not associated with SI; however, the use of alcohol, exercise, antiarrhythmic agents, and CCB was positively associated with SI. Finally, as previously reported, higher doses of statins were associated with a greater prevalence of SI.^5,7^

### Strength and limitations

Our meta-analysis has some limitations. Heterogeneity between studies was present in our analysis (*I*^2^ = 93–99%; unknown confounding may have led to this), although this was anticipated because of the broad scope of this systematic analysis, and due to very large data, we could not test the influence analysis that would resolve the effect size of different weight across the studies. The statistical examination of potential publication bias through Egger and funnel plots is not appropriate because studies with <100 patients were excluded from this systematic review.

Our analysis depended upon data reported in published studies. Some potential risk factors for SI were not reported with ideal detail or precision, such as the amount of alcohol consumption, types of exercise, and physical activity endurance. In this line, race distribution was not similar with predominantly Caucasian/White race (81.1%). It is also important to emphasize the importance of the nocebo/drucebo effect that was not examined in the included studies and might have distorted the final results to some extent (it might be responsible even for >50% of SAMS).^202,206^ However, besides the new effective one-of-trial approach that does not apply in clinical practice, we do not have suitable tools to exclude this phenomenon.^199^ Moreover, in most of the included trials, the diagnosis was based on the approved definitions, and the final SI prevalence based on this was <7%, which suggests that the potential effect of the nocebo/drucebo effect seemed to be minimized.

The data obtained do not allow us to draw conclusions in relation to the doses of other drugs used in the included studies that could have interacted with statin therapy. Nor can we draw conclusions relating to the stage or severity of diseases such as those affecting the liver, kidney, and thyroid. Finally, our analysis cannot be used to suggest appropriate management techniques (e.g. doses of drugs and/or the severity of the diseases when statins might be used without increasing the risk of SI).

## Conclusion

Based on the data from >4 million patients, we demonstrated that the overall prevalence of SI is relatively low, especially when SI is objectively determined using the recognized international definitions. These results support the concept that the prevalence of complete SI is often overestimated and highlights the need for a very careful assessment of patients with SI, to decrease the risk of unnecessary statin discontinuation, and suboptimal lipid-lowering therapy. Clinicians should use these results to encourage adherence to statin therapy in their patients.

## Supplementary material

Supplementary material is available at *European Heart Journal* online.

## Supplementary Material

ehac015_Supplementary_Data

## References

[1] Roth GA , Mensah GA, Johnson CO, Addolorato G, Ammirati E, Baddour LM, et al Global burden of cardiovascular diseases and risk factors, 1990–2019: update from the GBD 2019 study. J Am Coll Cardiol 2020;76:2982–3021. 33309175 10.1016/j.jacc.2020.11.010 PMC7755038

[2] Stone NJ , Robinson J, Lichtenstein AH, Bairey Merz CN, Blum CB, Eckel RH, et al 2013 ACC/AHA guideline on the treatment of blood cholesterol to reduce atherosclerotic cardiovascular risk in adults: a report of the American College of Cardiology/American Heart Association Task Force on Practice Guidelines. J Am Coll Cardiol 2014;63:2889–2934. 24239923 10.1016/j.jacc.2013.11.002

[3] Banach M , Stulc T, Dent R, Toth PP. Statin non-adherence and residual cardiovascular risk: there is need for substantial improvement. Int J Cardiol 2016;225:184–196. 27728862 10.1016/j.ijcard.2016.09.075

[4] Toth PP , Patti AM, Giglio RV, Nikolic D, Castellino G, Rizzo M, et al Management of statin intolerance in 2018: still more questions than answers. Am J Cardiovasc Drugs 2018;18:157–173. 29318532 10.1007/s40256-017-0259-7 PMC5960491

[5] Rosenson RS , Baker S, Banach M, Borow KM, Braun LT, Bruckert E, et al Optimizing cholesterol treatment in patients with muscle complaints. J Am Coll Cardiol 2017;70:1290–1301. 28859793 10.1016/j.jacc.2017.07.752

[6] Stroes ES , Thompson PD, Corsini A, Vladutiu GD, Raal FJ, Ray KK, et al Statin-associated muscle symptoms: impact on statin therapy—European Atherosclerosis Society Consensus Panel Statement on Assessment, Aetiology and Management. Eur Heart J 2015;36:1012–1022. 25694464 10.1093/eurheartj/ehv043 PMC4416140

[7] Banach M , Rizzo M, Toth PP, Farnier M, Davidson MH, Al-Rasadi K, et al Statin intolerance: an attempt at a unified definition. Position paper from an International Lipid Expert Panel. Arch Med Sci 2015;11:1–23. 25861286 10.5114/aoms.2015.49807 PMC4379380

[8] Guyton JR , Bays HE, Grundy SM, Jacobson TA, The National Lipid Association Statin Intolerance Panel. An assessment by the Statin Intolerance Panel: 2014 update. J Clin Lipidol 2014;8(3 Suppl):S72–S81. 24793444 10.1016/j.jacl.2014.03.002

[9] Mancini GB , Baker S, Bergeron J, Fitchett D, Frohlich J, Genest J, et al Diagnosis, prevention, and management of statin adverse effects and intolerance: Canadian Consensus Working Group Update (2016). Can J Cardiol 2016;32:S35–S65. 27342697 10.1016/j.cjca.2016.01.003

[10] Sposito A , Rocha Faria J, de Carvalho L, Lorenzatti A, Cafferata A, Elikir G, et al Statin-associated muscle symptoms: position paper from the Luso-Latin American Consortium. Curr Med Res Opin 2017;33:239–251. 27776432 10.1080/03007995.2016.1252740

[11] Banach M , Mikhailidis DP. Statin intolerance: some practical hints. Cardiol Clin 2018;36:225–231. 29609752 10.1016/j.ccl.2017.12.004

[12] Keen HI , Krishnarajah J, Bates TR, Watts GF. Statin myopathy: the fly in the ointment for the prevention of cardiovascular disease in the 21st century? Expert Opin Drug Saf 2014;13:1227–1239. 25017015 10.1517/14740338.2014.937422

[13] Page MJ , McKenzie JE, Bossuyt PM, Boutron I, Hoffmann TC, Mulrow CD, et al The PRISMA 2020 statement: an updated guideline for reporting systematic reviews. BMJ 2021;372:n71. 33782057 10.1136/bmj.n71 PMC8005924

[14] Higgins JPT , Thomas J, Chandler J, Cumpston M, Li T, Page MJ, et al Cochrane Handbook for Systematic Reviews of Interventions version 6.2. Cochrane, 2021. www.training.cochrane.org/handbook.

[15] Zeng X , Zhang Y, Kwong JS, Zhang C, Li S, Sun F, et al The methodological quality assessment tools for preclinical and clinical studies, systematic review and meta-analysis, and clinical practice guideline: a systematic review. J Evid Based Med 2015;8:2–10. 25594108 10.1111/jebm.12141

[16] Hozo SP , Djulbegovic B, Hozo I. Estimating the mean and variance from the median, range, and the size of a sample. BMC Med Res Methodol 2005;5:13. 15840177 10.1186/1471-2288-5-13 PMC1097734

[17] Higgins JPT , Thompson SG. Quantifying heterogeneity in a meta-analysis. Stat Med 2002;21:1539–1558. 12111919 10.1002/sim.1186

[18] Harbord RM , Egger M, Sterne JA. A modified test for small-study effects in meta-analyses of controlled trials with binary endpoints. Stat Med 2006;25:3443–3457. 16345038 10.1002/sim.2380

[19] Kannel WB , D’Agostino RB, Stepanians M, D’Agostino LC. Efficacy and tolerability of lovastatin in a six-month study: analysis by gender, age and hypertensive status. Am J Cardiol 1990;66:1B–10B. 2206030 10.1016/0002-9149(90)90434-3

[20] Bradford RH , Shear CL, Chremos AN, Dujovne C, Downton M, Franklin FA, et al Expanded Clinical Evaluation of Lovastatin (EXCEL) study results. I. Efficacy in modifying plasma lipo-proteins and adverse event profile in 8245 patients with moderate hypercholesterolemia. Arch Intern Med 1991;151:43–49. 1985608 10.1001/archinte.151.1.43

[21] Crepaldi G , Baggio G, Arca M, Avellone G, Avogaro P, Bittolo Bon G, et al Pravastatin vs gemfibrozil in the treatment of primary hypercholesterolemia: the Italian Multicenter Pravastatin Study I. Arch Intern Med 1991;151:146–152. 1898694

[22] Pravastatin Multicenter Study Group II . Comparative efficacy and safety of pravastatin and cholestyramine alone and combined in patients with hypercholesterolemia. Arch Intern Med 1993;153:1321–1329. 8507122

[23] The Pravastatin Multinational Study Group for Cardiac Risk Patients . Effects of pravastatin in patients with serum total cholesterol levels from 5.2 to 7.8 mmol/liter (200 to 300 mg/dl) plus two additional atherosclerotic risk factors. Am J Cardiol 1993;72:1031–1037. 8213583 10.1016/0002-9149(93)90858-a

[24] Blankenhorn DH , Azen SP, Kramsch DM, Mack WJ, Cashin-Hemphill L, Hodis HN, et al Coronary angiographic changes with lovastatin therapy: the Monitored Atherosclerosis Regression Study (MARS). Ann Intern Med 1993;119:969–976. 8214993 10.7326/0003-4819-119-10-199311150-00002

[25] Wiklund O , Angelin B, Bergman M, Berglund L, Bondjers G, Carlsson A, et al Pravastatin and gemfibrozil alone and in combination for the treatment of hypercholesterolemia. Am J Med 1993;94:13–20. 8420296 10.1016/0002-9343(93)90114-5

[26] Furberg CD , Adams HP Jr, Applegate WB, Byington RP, Espeland MA, Hartwell T, et al Effect of lovastatin on early carotid atherosclerosis and cardiovascular events. Asymptomatic Carotid Artery Progression Study (ACAPS) Research Group. Circulation 1994;90:1679–1687. 7734010 10.1161/01.cir.90.4.1679

[27] Keech A , Collins R, MacMahon S, Armitage J, Lawson A, Wallendszus K, et al Three-year follow-up of the Oxford Cholesterol Study: assessment of the efficacy and safety of simvastatin in preparation for a large mortality study. Eur Heart J 1994;15:255–269. 8005129 10.1093/oxfordjournals.eurheartj.a060485

[28] Insull W Jr , Black D, Dujovne C, Hosking JD, Hunninghake D, Keilson L, et al Efficacy and safety of once-daily vs twice-daily dosing with fluvastatin, a synthetic reductase inhibitor, in primary hypercholesterolemia. Arch Intern Med 1994;154:2449–2455. 7979841

[29] Jacotot B , Banga JD, Pfister P, Mehra M, for the French-Dutch Fluvastatin Study Group. Efficacy of a low dose-range of fluvastatin (XU 62-320) in the treatment of primary hypercholesterolaemia: a dose–response study in 431 patients. Br J Clin Pharmacol 1994;38:257–263. 7826828 10.1111/j.1365-2125.1994.tb04350.x PMC1364798

[30] Salonen R , Nyyssonen K, Porkkala E, Rummukainen J, Belder R, Park JS, et al Kuopio Atherosclerosis Prevention Study (KAPS): a population-based primary preventive trial of the effect of LDL lowering on atherosclerotic progression in carotid and femoral arteries. Circulation 1995;92:1758–1764. 7671358 10.1161/01.cir.92.7.1758

[31] Shepherd J , Cobbe SM, Ford I, Isles CG, Lorimer AR, MacFarlane PW, et al Prevention of coronary heart disease with pravastatin in men with hypercholesterolemia: West of Scotland Coronary Prevention Study Group. N Engl J Med 1995;333:1301–1307. 7566020 10.1056/NEJM199511163332001

[32] Andrade SE , Walker AM, Gottlieb LK, Hollenberg NK, Testa MA, Saperia GM, et al Discontinuation of antihyperlipidemic drugs—do rates reported in clinical trials reflect rates in primary care settings? N Engl J Med 1995;332:1125–1131. 7700285 10.1056/NEJM199504273321703

[33] Pitt B , Mancini GB, Ellis SG, Rosman HS, Park JS, McGovern ME. Pravastatin limitation of atherosclerosis in the coronary arteries (PLAC I): reduction in atherosclerosis progression and clinical events. PLAC I investigation. J Am Coll Cardiol 1995;26:1133–1139. 7594023 10.1016/0735-1097(95)00301-0

[34] Jukema JW , Bruschke AV, van Boven AJ, Reiber JH, Bal ET, Zwinderman AH, et al Effects of lipid lowering by pravastatin on progression and regression of coronary artery disease in symptomatic men with normal to moderately elevated serum cholesterol levels. The Regression Growth Evaluation Statin Study (REGRESS). Circulation 1995;91:2528–2540. 7743614 10.1161/01.cir.91.10.2528

[35] Simons LA , LevIs G, Simons J. Apparent discontinuation rates in patients prescribed lipid-lowering drugs. Med J Aust 1996;164:208–211. 8604188 10.5694/j.1326-5377.1996.tb94138.x

[36] Sacks FM , Pfeffer MA, Moye LA, Rouleau JL, Rutherford JD, Cole TG, et al The effect of pravastatin on coronary events after myocardial infarction in patients with average cholesterol levels. Cholesterol and Recurrent Events Trial Investigators. N Engl J Med 1996;335:1001–1009. 8801446 10.1056/NEJM199610033351401

[37] Bertolini S , Bon GB, Campbell LM, Farnier M, Langan J, Mahla G, et al Efficacy and safety of atorvastatin compared to pravastatin in patients with hypercholesterolemia. Atherosclerosis 1997;130:191–197. 9126664 10.1016/s0021-9150(96)06052-2

[38] Dart A , Jerums G, Nicholson G, d’Emden M, d’Emden M, Hamilton-Craig I, et al A multicenter, double-blind, one-year study comparing safety and efficacy of atorvastatin versus simvastatin in patients with hypercholesterolemia. Am J Cardiol 1997;80:39–44. 9205017 10.1016/s0002-9149(97)00280-4

[39] Davidson M , McKenney J, Stein E, Schrott H, Bakker-Arkema R, Fayyad R, et al Comparison of the one-year efficacy and safety of atorvastatin versus lovastatin in primary hypercholesterolemia. Am J Cardiol 1997;79:1475–1481. 9185636 10.1016/s0002-9149(97)00174-4

[40] Herd JA , Ballantyne CM, Farmer JA, Ferguson JJ III, Jones PH, West MS, et al Effects of fluvastatin on coronary atherosclerosis in patients with mild to moderate cholesterol elevations (Lipoprotein and Coronary Atherosclerosis Study [LCAS]). Am J Cardiol 1997;80:278–286. 9264419 10.1016/s0002-9149(97)00346-9

[41] Jones P , Kafonek S, Laurora I, Hunninghake D, for the CURVES Investigators. Comparative dose efficacy study of atorvastatin versus simvastatin, pravastatin, lovastatin and fluvastatin in patients with hypercholesterolemia (the CURVES study). Am J Cardiol 1998;81:582–587. 9514454 10.1016/s0002-9149(97)00965-x

[42] Long-Term Intervention with Pravastatin in Ischaemic Disease (LIPID) Study Group . Prevention of cardiovascular events and death with pravastatin in patients with coronary heart disease and a broad range of initial cholesterol levels. N Engl J Med 1998;339:1349–1357. 9841303 10.1056/NEJM199811053391902

[43] Downs JR , Clearfield M, Weis S, Whitney E, Shapiro DR, Beere PA, et al Primary prevention of acute coronary events with lovastatin in men and women with average cholesterol levels: results of AFCAPS/TexCAPS. Air Force/Texas Coronary Atherosclerosis Prevention Study. JAMA 1998;279:1615–1622. 9613910 10.1001/jama.279.20.1615

[44] Eriksson M , Hadell K, Holme I, Walldius G, Kjellström T. Compliance with and efficacy of treatment with pravastatin and cholestyramine. J Intern Med 1998;243:373–380. 9651560 10.1046/j.1365-2796.1998.00294.x

[45] Hlatt JG , Shamsle SG, Schectman G. Discontinuation rates of cholesterol lowering medications: implications for primary care. Am J Manag Care 1999;5:437–444. 10387383

[46] Bruckert E , Simonetta C, Giral P. Compliance with fluvastatin treatment characterization of the noncompliant population within a population of 3845 patients with hyperlipidemia. CREOLE Study Team. J Clin Epidemiol 1999;52:589–594. 10408999 10.1016/s0895-4356(99)00019-0

[47] Serruys PW , Foley DP, Jackson G, Bonnier H, Macaya C, Vrolix M, et al A randomized placebo-controlled trial of fluvastatin for prevention of restenosis after successful coronary balloon angioplasty; final results of the fluvastatin angiographic restenosis (FLARE) trial. Eur Heart J 1999;20:58–69. 10075142 10.1053/euhj.1998.1150

[48] Maerz W , Wollschlaeger H, Klein G, Neiss A, Wehling M. Safety of low-density lipoprotein cholesterol reduction with atorvastatin versus simvastatin in a coronary heart disease population (the TARGET TANGIBLE trial). Am J Cardiol 1999;84:7–13. 10404843 10.1016/s0002-9149(99)00183-6

[49] Barter PJ , O’Brien RC. Achievement of target plasma cholesterol levels in hypercholesterolaemic patients being treated in general practice. Atherosclerosis 2000;149:199–205. 10704632 10.1016/s0021-9150(99)00402-5

[50] Gentile S , Turco S, Guarino G, Sasso CF, Amodio M, Magliano P, et al Comparative efficacy study of atorvastatin vs. simvastatin, pravastatin, lovastatin and placebo in type 2 diabetic patients with hypercholesterolaemia. Diabetes Obes Metab 2000;2:355–362. 11225965 10.1046/j.1463-1326.2000.00106.x

[51] Schwartz GG , Olsson AG, Ezekowitz MD, Ganz P, Oliver MF, Waters D, et al Effects of atorvastatin on early recurrent ischemic events in acute coronary syndromes: the MIRACL study: a randomized controlled trial. JAMA 2001;28:1711–1718. 10.1001/jama.285.13.1711 11277825

[52] Olsson AG , Pears J, McKellar J, Mizan J, Raza A. Effect of rosuvastatin on low-density lipoprotein cholesterol in patients with hypercholesterolemia. Am J Cardiol 2001;88:504–508. 11524058 10.1016/s0002-9149(01)01727-1

[53] Smilde TJ , van Wissen S, Wollersheim H, Trip MD, Kastelein JJ, Stalenhoef AF. Effect of aggressive versus conventional lipid lowering on atherosclerosis progression in familial hypercholesterolaemia (ASAP): a prospective, randomised, double-blind trial. Lancet 2001;357:577–581. 11558482 10.1016/s0140-6736(00)04053-8

[54] Andrews TC , Ballantyne CM, Hsia JA, Kramer JH. Achieving and maintaining National Cholesterol Education Program low-density lipoprotein cholesterol goals with five statins. Am J Med 2001;111:185–191. 11530028 10.1016/s0002-9343(01)00799-9

[55] Insull W , Kafonek S, Goldner D, Zieve F, ASSET Investigators. Comparison of efficacy and safety of *atorvastatin* (10 mg) with *simvastatin* (10 mg) at six weeks. Am J Cardiol 2001;87:554–559. 11230838 10.1016/s0002-9149(00)01430-2

[56] Branchi A , Fiorenza AM, Torri A, Muzio F, Berra C, Colombo E, et al Effects of low doses of simvastatin and atorvastatin on high-density lipoprotein cholesterol levels in patients with hypercholesterolemia. Clin Ther 2001;23:851–857. 11440285 10.1016/s0149-2918(01)80073-4

[57] Illingworth DR , Crouse JR III, Hunninghake DB, Davidson MH, Escobar ID, Stalenhoef AFH, et al A comparison of simvastatin and atorvastatin up to maximal recommended doses in a large multicenter randomized clinical trial. Curr Med Res Opin 2001;17:43–50. 11464446

[58] Diabetes Atorvastin Lipid Intervention (DALI) Study Group . The effect of aggressive versus standard lipid lowering by atorvastatin on diabetic dyslipidemia: the DALI study: a double-blind, randomized, placebo-controlled trial in patients with type 2 diabetes and diabetic dyslipidemia. Diabetes Care 2001;24:1335–1341. 11473066 10.2337/diacare.24.8.1335

[59] Hunninghake D , Insull W Jr, Toth P, Davidson D, Donovan JM, Burke SK. Coadministration of colesevelam hydrochloride with atorvastatin lowers LDL cholesterol additively. Atherosclerosis 2001;158:407–416. 11583720 10.1016/s0021-9150(01)00437-3

[60] Saito Y , Shirai K, Sasaki N, Shinomiya M, Yoshida S, Committee of the Chiba Lipid Intervention Program Study. Prognosis of hypercholesterolemic patients taking pravastatin for five years: the Chiba Lipid Intervention Program (CLIP) study. J Atherscler Thromb 2002;9:99–108. 10.5551/jat.9.99 12236319

[61] Jackevicius CA , Mamdani M, Tu JV. Adherence with statin therapy in elderly patients with and without acute coronary syndromes. JAMA 2002;288:462–467. 12132976 10.1001/jama.288.4.462

[62] Benner JS , Glynn RJ, Mogun H, Neumann PJ, Weinstein MC, Avorn J. Long-term persistence in use of statin therapy in elderly patients. JAMA 2002;288:455–461. 12132975 10.1001/jama.288.4.455

[63] Larsen J , Andersen M, Kragstrup J, Gram LF. High persistence of statin use in a Danish population: compliance study 1993–1998. Br J Clin Pharmacol 2002;53:375–378. 11966668 10.1046/j.1365-2125.2002.01563.x PMC1874277

[64] Heart Protection Study Collaborative Group . MRC/BHF Heart Protection Study of cholesterol lowering with simvastatin in 20 536 high risk individuals: a randomised placebo-controlled trial. Lancet 2002;360:7–22.12114036

[65] Shepherd J , Blauw GJ, Murphy MB, Bollen EL, Buckley BM, Cobbe SM, et al Pravastatin in elderly individuals at risk of vascular disease (PROSPER): a randomised controlled trial. Lancet 2002;360:1623–1630. 12457784 10.1016/s0140-6736(02)11600-x

[66] Serruys PW , de Feyter P, Macaya C, Kokott N, Puel J, Vrolix M, et al Fluvastatin for prevention of cardiac events following successful first percutaneous coronary intervention: a randomized controlled trial. JAMA 2002;287:3215–3222. 12076217 10.1001/jama.287.24.3215

[67] Wei L , Wang J, Thompson P, Wong S, Struthers AD, MacDonald TM. Adherence to statin treatment and readmission of patients after myocardial infarction: a six year follow up study. Heart 2002;88:229–233. 12181210 10.1136/heart.88.3.229 PMC1767352

[68] Karalis DG , Ross AM, Vacari RM, Zarren H, Scott R. Comparison of efficacy and safety of atorvastatin and simvastatin in patients with dyslipidemia with and without coronary heart disease. Am J Cardiol 2002;89:667–671. 11897207 10.1016/s0002-9149(01)02337-2

[69] Matsuzaki M , Kita T, Mabuchi H, Matsuzawa Y, Nakaya N, Oikawa S, et al Large scale cohort study of the relationship between serum cholesterol concentration and coronary events with low-dose simvastatin therapy in Japanese patients with hypercholesterolemia. Circ J 2002;66:1087–1095. 12499611 10.1253/circj.66.1087

[70] Mohler ER III , Hiatt WR, Creager MA. Cholesterol reduction with atorvastatin improves walking distance in patients with peripheral arterial disease. Circulation 2003;108:1481–1486. 12952839 10.1161/01.CIR.0000090686.57897.F5

[71] Bruckert E , Lievre M, Giral P, Crepaldi G, Masana L, Vrolix M, et al Short-term efficacy and safety of extended-release fluvastatin in a large cohort of elderly patients. Am J Geriatr Cardiol 2003;12:225–231. 12888702 10.1111/j.1076-7460.2003.02000.x

[72] Ballantyne CM , Houri J, Notarbartolo A, Melani L, Lipka LJ, Suresh R, et al Effect of ezetimibe coadministered with atorvastatin in 628 patients with primary hypercholesterolemia: a prospective, randomized, double-blind trial. Circulation 2003;107:2409–2415. 12719279 10.1161/01.CIR.0000068312.21969.C8

[73] Kerzner B , Corbelli J, Sharp S, Lipka LJ, Melani L, LeBeaut A, et al Efficacy and safety of ezetimibe coadministered with lovastatin in primary hypercholesterolemia. Am J Cardiol 2003;91:418–424. 12586255 10.1016/s0002-9149(02)03236-8

[74] Rosenson RS , Bays HE. Results of two clinical trials on the safety and efficacy of pravastatin 80 and 160 mg per day. Am J Cardiol 2003;91:878–881. 12667578 10.1016/s0002-9149(03)00026-2

[75] Yang CC , Jick SS, Testa MA. Discontinuation and switching of therapy after initiation of lipid-lowering drugs: the effects of comorbidities and patient characteristics. Br J Clin Pharmacol 2003;56:84–91. 12848779 10.1046/j.1365-2125.2003.01818.x PMC1884321

[76] Abraha I , Montedori A, Stracci F, Rossi M, Romagnoli C. Statin compliance in the Umbrian population. Eur J Clin Pharmacol 2003;59:659–661. 14508622 10.1007/s00228-003-0675-2

[77] Holdaas H , Fellström B, Jardine AG, Holme I, Nyberg G, Fauchald P, et al Effect of fluvastatin on cardiac outcomes in renal transplant recipients: a multicentre, randomised, placebo-controlled trial. Lancet 2003;361:2024–2031. 12814712 10.1016/S0140-6736(03)13638-0

[78] Olsson AG , Eriksson M, Johnson O, Kjellstrom T, Lanke J, Larsen ML, et al A 52-week, multicenter, randomized, parallel-group, double-blind, double-dummy study to assess the efficacy of atorvastatin and simvastatin in reaching low-density lipoprotein cholesterol and triglyceride targets: the Treat-to-Target (3T) study. Clin Ther 2003;25:119–138. 12637115 10.1016/s0149-2918(03)90015-4

[79] Ballantyne CM , Blazing MA, Hunninghake DB, Davidson MH, Yuan Z, DeLucca P, et al Effect on high-density lipoprotein cholesterol of maximum dose simvastatin and atorvastatin in patients with hypercholesterolemia: results of the Comparative HDL Efficacy and Safety Study (CHESS). Am Heart J 2003;146:862–869. 14597936 10.1016/S0002-8703(03)00440-X

[80] Schneck DW , Knopp RH, Ballantyne CM, McPherson R, Chitra RR, Simonson SG. Comparative effects of rosuvastatin and atorvastatin across their dose ranges in patients with hypercholesterolemia and without active arterial disease. Am J Cardiol 2003;91:33–41. 12505568 10.1016/s0002-9149(02)02994-6

[81] Stein EA , Strutt K, Southworth H, Diggle PJ, Miller E, HeFH Study Group. Comparison of rosuvastatin versus atorvastatin in patients with heterozygous familial hypercholesterolemia. Am J Cardiol 2003;92:1287–1293. 14636905 10.1016/j.amjcard.2003.08.009

[82] Schuster H , Barter PJ, Stender S, Cheung RC, Bonnet J, Morrell JM, et al Effects of switching statins on achievement of lipid goals: measuring effective reductions in cholesterol using rosuvastatin therapy (MERCURY I) study. Am Heart J 2004;147:705–712. 15077101 10.1016/j.ahj.2003.10.004

[83] Schwartz GG , Bolognese MA, Tremblay BP, Cheung RC, Bonnet J, Morrell JM, et al Efficacy and safety of rosuvastatin and atorvastatin in patients with hypercholesterolemia and a high risk of coronary heart disease: a randomized, controlled trial. Am Heart J 2004;148:e4. 15215813 10.1016/j.ahj.2004.01.020

[84] Beishuizen ED , Ree MA, Jukema JW, Tamsma JT, Vijver JC, Meinders AE, et al Two year statin therapy does not alter the progression of intima medica thickness in patients with type 2 diabetes without manifest cardiovascular disease. Diabetes Care 2004;27:2887–2891. 15562202 10.2337/diacare.27.12.2887

[85] Hunninghake DB , Stein EA, Bays HE, Rader DJ, Chitra RR, Simonson SG, et al Rosuvastatin improves the atherogenic and atheroprotective lipid profiles in patients with hypertriglyceridemia. Coron Artery Dis 2004;15:115–123. 15024300 10.1097/00019501-200403000-00008

[86] Goldberg AC , Sapre A, Liu J, Capece R, Mitchel YB. Efficacy and safety of ezetimibe coadministered with simvastatin in patients with primary hypercholesterolemia: a randomized, double-blind, placebo-controlled trial. Mayo Clin Proc 2004;79:620–629. 15132403 10.4065/79.5.620

[87] Ellis JJ , Erickson SR, Stevenson JG, Bernstein SJ, Stiles RA, Fendrick AM. Suboptimal statin adherence and discontinuation in primary and secondary prevention populations. J Gen Intern Med 2004;19:638–645. 15209602 10.1111/j.1525-1497.2004.30516.x PMC1492382

[88] Cannon CP , Braunwald E, McCabe CH, Rader DJ, Rouleau JL, Belder R, et al Intensive versus moderate lipid lowering with statins after acute coronary syndromes. N Engl J Med 2004;350:1495–1504. 15007110 10.1056/NEJMoa040583

[89] Nissen SE , Tuzcu EM, Schoenhagen P, Brown BG, Ganz P, Vogel RA, et al Effect of intensive compared with moderate lipid-lowering therapy on progression of coronary atherosclerosis: a randomized controlled trial. JAMA 2004;291:1071–1080. 14996776 10.1001/jama.291.9.1071

[90] Benner JS , Tierce JC, Ballantyne CM, Prasad C, Bullano MF, Willey VJ, et al Follow-up lipid tests and physician visits are associated with improved adherence to statin therapy. Pharmacoeconomics 2004;22:13–23. 10.2165/00019053-200422003-00003 15669150

[91] Eagle KA , Kline-Rogers E, Goodman SG, Gurfinkel EP, Avezum A, Flather MD, et al Adherence to evidence-based therapies after discharge for acute coronary syndromes: an ongoing prospective, observational study. Am J Med 2004;117:73–81. 15234641 10.1016/j.amjmed.2003.12.041

[92] Howell N , Trotter R, Mottram DR, Rowe P. Compliance with statins in primary care. Pharm J 2004;272:23–32.

[93] Koren MJ , Hunninghake DB. Clinical outcomes in managed-care patients with coronary heart disease treated aggressively in lipid lowering disease management clinics: the alliance study. J Am Coll Cardiol 2004;44:1772–1779. 15519006 10.1016/j.jacc.2004.07.053

[94] Colhoun HM , Betteridge DJ, Durrington PN, Hitman GA, Neil HA, Livingstone SJ, et al Primary prevention of cardiovascular disease with atorvastatin in type 2 diabetes in the Collaborative Atorvastatin Diabetes Study: multicentre randomised placebo-controlled trial. Lancet 2004;364:685–696. 15325833 10.1016/S0140-6736(04)16895-5

[95] Scandinavian Simvastatin Survival Study Group . Randomized trial of cholesterol lowering in 4444 patients with coronary heart disease: the Scandinavian Simvastatin Survival Study (4S). Lancet 1994;344:1383–1389. 7968073

[96] De Lemos JA , Blazing MA, Wiviott SD, Lewis EF, Fox KA, White HD, et al Early intensive vs a delayed conservative simvastatin strategy in patients with acute coronary syndromes: phase Z of the A to Z trial. JAMA 2004;292:1307–1316. 15337732 10.1001/jama.292.11.1307

[97] Adverse Drug Reactions Advisory Committee (ADRAC) . Risk factors for myopathy and rhabdomyolysis with the statins. Aust Adv Drug Reactions Bull 2004;23:1–2.

[98] Bruckert E , Hayem G, Dejager S, Yau C, Bégaud B. Mild to moderate muscular symptoms with high-dosage statin therapy in hyperlipidemic patients—the PRIMO Study. Cardiovasc Drugs Ther 2005;19:403–414. 16453090 10.1007/s10557-005-5686-z

[99] Caspard H , Chan AK, Walker AM. Compliance with a statin treatment in a usual-care setting: retrospective database analysis over 3 years after treatment initiation in health maintenance organization enrollees with dyslipidemia. Clin Ther 2005;27:1639–1646. 16330301 10.1016/j.clinthera.2005.10.005

[100] Perreault S , Blais L, Lamarre D, Dragomir A, Berbiche D, Lalonde L, et al Persistence and determinants of statin therapy among middle-aged patients for primary and secondary prevention. Br J Clin Pharmacol 2005;59:564–573. 15842555 10.1111/j.1365-2125.2005.02355.x PMC1884848

[101] Blackburn DF , Dobson RT, Blackburn JL, Wilson TW. Cardiovascular morbidity associated with nonadherence to statin therapy. Pharmacotherapy 2005;25:1035–1043. 16207093 10.1592/phco.2005.25.8.1035

[102] La Rosa JC , Grundy SM, Waters DD, Shear C, Barter P, Fruchart JC, et al Intensive lipid lowering with atorvastatin in patients with stable coronary disease. N Engl J Med 2005;352:1425–1435. 15755765 10.1056/NEJMoa050461

[103] Pedersen TR , Faergeman O, Kastelein JJ, Olsson AG, Tikkanen MJ, Holme I, et al High-dose atorvastatin vs usual-dose simvastatin for secondary prevention after myocardial infarction. JAMA 2005;294:2437–2445. 16287954 10.1001/jama.294.19.2437

[104] Amarenco P , Bogousslavsky J, Callahan A III, Goldstein LB, Hennerici M, Rudolph AE, et al High-dose atorvastatin after stroke or transient ischemic attack. N Engl J Med 2006;355:549–559. 16899775 10.1056/NEJMoa061894

[105] Knopp RH , d’Emden M, Smilde JG, Pocock SJ. Efficacy and safety of atorvastatin in the prevention of cardiovascular end points in subjects with type 2 diabetes: the Atorvastatin Study for Prevention of Coronary Heart Disease Endpoints in non-insulin-dependent diabetes mellitus (ASPEN). Diabetes Care 2006;29:1478–1485. 16801565 10.2337/dc05-2415

[106] Nakamura H , Arakawa K, Itakura H, Kitabatake A, Goto Y, Toyota T, et al Primary prevention of cardiovascular disease with pravastatin in Japan: a prospective randomised controlled trial. Lancet 2006;368:1155–1163. 17011942 10.1016/S0140-6736(06)69472-5

[107] Nissen SE , Nicholls SJ, Sipahi I, Libby P, Raichlen JS, Ballantyne CM, et al Effect of very high-intensity statin therapy on regression of coronary atherosclerosis: the ASTEROID trial. JAMA 2006;295:1556–1565. 16533939 10.1001/jama.295.13.jpc60002

[108] Save V , Patil N, Moulik N, Rajadhyaksha G. Effect of atorvastatin on type 2 diabetic dyslipidemia. J Cardiovasc Pharmacol Ther 2006;11:262–270. 17220473 10.1177/1074248406295523

[109] Goldberg RB , Guyton JR, Mazzone T, Weinstock RS, Polis A, Edwards P, et al Ezetimibe/simvastatin vs atorvastatin in patients with type 2 diabetes mellitus and hypercholesterolemia: the VYTAL study. Mayo Clin Proc 2006;81:1579–1588. 17165637 10.4065/81.12.1579

[110] Binbrek AS , Elis A, Al-Zaibag M, Eha J, Keber I, Cuevas AM, et al Rosuvastatin versus atorvastatin in achieving lipid goals in patients at high risk for cardiovascular disease in clinical practice: a randomized, open-label, parallel-group, multicenter study (DISCOVERY alpha study). Curr Ther Res Clin Exp 2006;67:21–43. 24936119 10.1016/j.curtheres.2006.02.005 PMC4052636

[111] Clearfield MB , Amerena J, Bassand JP, García HRH, Miller SS, Sosef FFM, et al Comparison of the efficacy and safety of rosuvastatin 10 mg and atorvastatin 20 mg in high-risk patients with hypercholesterolemia—Prospective study to evaluate the Use of Low doses of the Statins Atorvastatin and Rosuvastatin (PULSAR). Trials 2006;7:35. 17184550 10.1186/1745-6215-7-35 PMC1779361

[112] Betteridge DJ , Gibson JM. Effects of rosuvastatin on lipids, lipoproteins and apolipoproteins in the dyslipidaemia of diabetes. Diabet Med 2007;24:541–549. 17367312 10.1111/j.1464-5491.2007.02095.x

[113] Blagden MD , Chipperfield R, Blagden MD, Chipperfield R. Efficacy and safety of ezetimibe co-administered with atorvastatin in untreated patients with primary hypercholesterolaemia and coronary heart disease. Curr Med Res Opin 2007;23:767–775. 17407633 10.1185/030079907x182059

[114] Lee SH , Chung N, Kwan J, Kim DI, Kim WH, Kim CJ, et al Comparison of the efficacy and tolerability of pitavastatin and atorvastatin: an 8-week, multicenter, randomized, open-label, dose-titration study in Korean patients with hypercholesterolemia. Clin Ther 2007;29:2365–2373. 18158077 10.1016/j.clinthera.2007.11.002

[115] McGinnis B , Olson KL, Magid D, Bayliss E, Korner EJ, Brand DW, et al Factors related to adherence to statin therapy. Ann Pharmacother 2007;41:1805–1811. 17925498 10.1345/aph.1K209

[116] Hudson M , Richard H, Pilote L. Parabolas of medication use and discontinuation after myocardial infarction—are we closing the treatment gap? Pharmacoepidemiol Drug Saf 2007;16:773–785. 17486661 10.1002/pds.1414

[117] Kamal-Bahl SJ , Burke T, Watson D, Wentworth C. Discontinuation of lipid modifying drugs among commercially insured United States patients in recent clinical practice. Am J Cardiol 2007;99:530–534. 17293198 10.1016/j.amjcard.2006.08.063

[118] Kjekshus J , Apetrei E, Barrios V, Böhm M, Cleland JG, Cornel JH, et al Rosuvastatin in older patients with systolic heart failure. N Engl J Med 2007;357:2248–2261. 17984166 10.1056/NEJMoa0706201

[119] Crouse JR III , Raichlen JS, Riley WA, Evans GW, Palmer MK, O’Leary DH, et al Effect of rosuvastatin on progression of carotid intima-media thickness in low-risk individuals with subclinical atherosclerosis: the METEOR Trial. JAMA 2007;297:1344–1353. 17384434 10.1001/jama.297.12.1344

[120] Leiter L , Rosenson RS, Stein E, Reckless JP, Schulte KL, Schleman M, et al Efficacy and safety of rosuvastatin 40 mg versus atorvastatin 80 mg in high-risk patients with hypercholesterolemia: results of the POLARIS study. Atherosclerosis 2007;94:e154–e164. 10.1016/j.atherosclerosis.2006.12.001 17229426

[121] Deedwania P , Stone PH, Bairey Merz CN, Cosin-Aguilar J, Koylan N, Luo D, et al Effects of intensive versus moderate lipid-lowering therapy on myocardial ischemia in older patients with coronary heart disease: results of the Study Assessing Goals in the Elderly (SAGE). Circulation 2007;115:700–707. 17283260 10.1161/CIRCULATIONAHA.106.654756

[122] Yu AP , Yu YF, Nichol MB, Gwadry-Sridhar F. Delay in filling the initial prescription for a statin: a potential early indicator of medication nonpersistence. Clin Ther 2008;30:761–774. 18498924 10.1016/j.clinthera.2008.04.015

[123] Donnelly LA , Doney AS, Morris AD, Palmer CN, Donnan PT. Long-term adherence to statin treatment in diabetes. Diabet Med 2008;25:850–855. 18644071 10.1111/j.1464-5491.2008.02476.x

[124] Chodick G , Shalev V, Gerber Y, Heymann AD, Silber H, Simah V, et al Long-term persistence with statin treatment in a not-for-profit health maintenance organization: a population-based retrospective cohort study in Israel. Clin Ther 2008;30:2167–2179. 19108805 10.1016/j.clinthera.2008.11.012

[125] Helin-Salmivaara A , Lavikainen P, Korhonen MJ, Halava H, Junnila SY, Kettunen R, et al Long-term persistence with statin therapy: a nationwide register study in Finland. Clin Ther 2008;30(Pt 2):2228–2240. 19281917 10.1016/j.clinthera.2008.12.003

[126] Tavazzi L , Maggioni AP, Marchioli R, Barlera S, Franzosi MG, Latini R, et al Effect of rosuvastatin in patients with chronic heart failure (the GISSI-HF trial): a randomised, double-blind, placebo-controlled trial. Lancet 2008;372:1231–1239. 18757089 10.1016/S0140-6736(08)61240-4

[127] Ridker PM , Danielson E, Fonseca FA, Genest J, Gotto AM Jr, Kastelein JJ, et al Rosuvastatin to prevent vascular events in men and women with elevated C-reactive protein. N Engl J Med 2008;359:2195–2207. 18997196 10.1056/NEJMoa0807646

[128] Newman CB , Szarek M, Colhoun HM, Betteridge DJ, Durrington PN, Hitman GA, et al The safety and tolerability of atorvastatin 10 mg in the Collaborative Atorvastatin Diabetes Study (CARDS). Diab Vasc Dis Res 2008;5:177–183. 18777490 10.3132/dvdr.2008.029

[129] Bonnet J , McPherson R, Tedgui A, Simoneau D, Nozza A, Martineau P, et al Comparative effects of 10-mg versus 80-mg atorvastatin on high-sensitivity C-reactive protein in patients with stable coronary artery disease: results of the CAP (Comparative Atorvastatin Pleiotropic effects) study. Clin Ther 2008;30:2298–2313. 19167589 10.1016/j.clinthera.2008.12.023

[130] Conard SE , Bays HE, Leiter LA, Bird SR, Rubino J, Lowe RS, et al Efficacy and safety of ezetimibe added on to atorvastatin (20 mg) versus up-titration of atorvastatin (to 40 mg) in hypercholesterolemic patients at moderately high risk for coronary heart disease. Am J Cardiol 2008;11:1489–1494. 10.1016/j.amjcard.2008.09.075 19026302

[131] Abate N , Catapano AL, Ballantyne CM, Davidson MH, Polis A, Smugar SS, et al Effect of ezetimibe/simvastatin versus atorvastatin or rosuvastatin on modifying lipid profiles in patients with diabetes, metabolic syndrome, or neither: results of two subgroup analyses. J Clin Lipidol 2008;2:91–105. 21291725 10.1016/j.jacl.2008.02.002

[132] Yokote K , Bujo H, Hanaoka H, Shinomiya M, Mikami K, Miyashita Y, et al Multicenter collaborative randomized parallel group comparative study of pitavastatin and atorvastatin in Japanese hypercholesterolemic patients. Collaborative study on hypercholesterolemia drug intervention and their benefits for atherosclerosis prevention (CHIBA study). Atherosclerosis 2008;201:345–352. 18472103 10.1016/j.atherosclerosis.2008.02.008

[133] Sasaki J , Ikeda Y, Kuribayashi T, Kajiwara K, Kajiwara K, Biro S, et al A 52-week, randomized, open-label, parallel-group comparison of the tolerability and effects of pitavastatin and atorvastatin on high-density lipoprotein cholesterol levels and glucose metabolism in Japanese patients with elevated levels of low-density lipoprotein cholesterol and glucose intolerance. Clin Ther 2008;30:1089–1101. 18640465 10.1016/j.clinthera.2008.05.017

[134] Yamazaki T , Kurabayashi M, ASTRO-2 Study Group. A randomized controlled study to compare the effect of rosuvastatin 5 mg with atorvastatin 10mg on plasma lipids in Japanese patients with hypercholesterolemia (ASTRO-2). Ann Vasc Dis 2009;2:159–173. 23555376 10.3400/avd.AVDoa090019 PMC3595734

[135] Budinski D , Arneson V, Hounslow N, Gratsiansky N. Pitavastatin compared with atorvastatin in primary hypercholesterolemia or combined dyslipidemia. Clin Lipidol 2009;4:291–302.

[136] Davidson MH , Rooney MW, Drucker J, Eugene Griffin H, Oosman S, Beckert M. Efficacy and tolerability of atorvastatin/fenofibrate fixed-dose combination tablet compared with atorvastatin and fenofibrate monotherapies in patients with dyslipidemia: a 12-week, multicenter, double-blind, randomized, parallel-group study. Clin Ther 2009;31:2824–2838. 20110022 10.1016/j.clinthera.2009.12.007

[137] Insull W Jr , Basile JN, Vo AN, Jiang P, Thakkar R, Padley RJ. Efficacy and safety of combination therapy with niacin extended release and simvastatin versus atorvastatin in patients with dyslipidemia: the SUPREME Study. J Clin Lipidol 2009;3:109–118. 21291800 10.1016/j.jacl.2009.02.009

[138] Fellström BC , Jardine AG, Schmieder RE, Holdaas H, Bannister K, Beutler J, et al Rosuvastatin and cardiovascular events in patients undergoing hemodialysis. N Engl J Med 2009;360:1395–1407. 19332456 10.1056/NEJMoa0810177

[139] Hall AS , Jackson BM, Farrin AJ, Efthymiou M, Barth JH, Copeland J, et al A randomized, controlled trial of simvastatin versus rosuvastatin in patients with acute myocardial infarction: the Secondary Prevention of Acute Coronary Events–Reduction of Cholesterol to Key European Targets Trial. Eur J Cardiovasc Prev Rehabil 2009;16:712–721. 19745745 10.1097/HJR.0b013e3283316ce8

[140] Corrao G , Conti V, Merlino L, Catapano AL, Mancia G. Results of a retrospective database analysis of adherence to statin therapy and risk of nonfatal ischemic heart disease in daily clinical practice in Italy. Clin Ther 2010;32:300–310. 20206788 10.1016/j.clinthera.2010.02.004

[141] Lablanche JM , Leone A, Merkely B, Morais J, Alonso J, Santini M, et al Comparison of the efficacy of rosuvastatin versus atorvastatin in reducing apolipoprotein B/apolipoprotein A-1 ratio in patients with acute coronary syndrome: results of the CENTAURUS study. Arch Cardiovasc Dis 2010;103:160–169. 20417447 10.1016/j.acvd.2010.01.005

[142] Kim SH , Park K, Hong SJ, Cho YS, Sung JD, Moon GW, et al Efficacy and tolerability of a generic and a branded formulation of atorvastatin 20 mg/d in hypercholesterolemic Korean adults at high risk for cardiovascular disease: a multicenter, prospective, randomized, double-blind, double-dummy clinical trial. Clin Ther 2010;32:1896–1905. 21095484 10.1016/j.clinthera.2010.10.004

[143] Park JS , Kim YJ, Choi JY, Kim YN, Hong TJ, Kim DS, et al Comparative study of low doses of rosuvastatin and atorvastatin on lipid and glycemic control in patients with metabolic syndrome and hypercholesterolemia. Korean J Intern Med 2010;25:27–35. 20195400 10.3904/kjim.2010.25.1.27 PMC2829413

[144] Armitage J , Bowman L, Wallendszus K, Bulbulia R, Rahimi K, Haynes R, et al Intensive lowering of LDL cholesterol with 80 mg versus 20 mg simvastatin daily in 12,064 survivors of myocardial infarction: a double-blind randomized trial. Lancet 2010;376:1658–1669. 21067805 10.1016/S0140-6736(10)60310-8 PMC2988223

[145] Ose L , Budinski D, Hounslow N, Arneson V. Long-term treatment with pitavastatin is effective and well tolerated by patients with primary hypercholesterolemia or combined dyslipidemia. Atherosclerosis 2010;210:202–208. 20080236 10.1016/j.atherosclerosis.2009.12.009

[146] Gumprecht J , Gosho M, Budinski D, Hounslow N. Comparative long-term efficacy and tolerability of pitavastatin 4 mg and atorvastatin 20–40 mg in patients with type 2 diabetes mellitus and combined (mixed) dyslipidaemia. Diabetes Obes Metab 2011;13:1047–1055. 21812889 10.1111/j.1463-1326.2011.01477.x

[147] Albert MA , Glynn RJ, Fonseca FA, Lorenzatti AJ, Ferdinand KC, MacFadyen JG, et al Race, ethnicity, and the efficacy of rosuvastatin in primary prevention: the Justification for the Use of statins in Prevention: an intervention Trial Evaluating Rosuvastatin (JUPITER) trial. Am Heart J 2011;162:106–114. 21742096 10.1016/j.ahj.2011.03.032

[148] Geers HC , Bouvy ML, Heerdink ER. Estimates of statin discontinuation rates are influenced by exposure and outcome definitions. Ann Pharmacother 2011;45:576–581. 21521863 10.1345/aph.1P607

[149] Harris LJ , Thapa R, Brown M, Pabbathi S, Childress RD, Heimberg M, et al Clinical and laboratory phenotype of patients experiencing statin intolerance attributable to myalgia. J Clin Lipidol 2011;5:299–307. 21784376 10.1016/j.jacl.2011.05.005

[150] Nicholls SJ , Ballantyne CM, Barter PJ, Chapman MJ, Erbel RM, Libby P, et al Effect of two intensive statin regimens on progression of coronary disease. N Engl J Med 2011;365:2078–2087. 22085316 10.1056/NEJMoa1110874

[151] Baigent C , Landray MJ, Reith C, Emberson J, Wheeler DC, Tomson C, et al The effects of lowering LDL cholesterol with simvastatin plus ezetimibe in patients with chronic kidney disease (Study of Heart and Renal Protection): a randomised placebo-controlled trial. Lancet 2011;377:2181–2192. 21663949 10.1016/S0140-6736(11)60739-3 PMC3145073

[152] Cohen JD , Brinton EA, Ito MK, Jacobson TA. Understanding Statin Use in America and Gaps in Patient Education (USAGE): an internet-based survey of 10,138 current and former statin users. J Clin Lipidol 2012;6:208–215. 22658145 10.1016/j.jacl.2012.03.003

[153] Esposti DL , Saragoni S, Batacchi P, Benemei S, Geppetti P, Sturani A, et al Adherence to statin treatment and health outcomes in an Italian cohort of newly treated patients: results from an administrative database analysis. Clin Ther 2012;34:190–199. 22284998 10.1016/j.clinthera.2011.12.011

[154] Pitt B , Loscalzo J, Monyak J, Miller E, Raichlen J. Comparison of lipid-modifying efficacy of rosuvastatin versus atorvastatin in patients with acute coronary syndrome (from the LUNAR study). Am J Cardiol 2012;109:1239–1246. 22360820 10.1016/j.amjcard.2011.12.015

[155] Nohara R , Daida H, Hata M, Kaku K, Kawamori R. Effect of intensive lipid lowering therapy with rosuvastatin of carotid intima-media thickness in Japanese patients: Justification of Atherosclorosis Regression Treatment (JART) study. Circulation 2012;76:221–229. 10.1253/circj.cj-11-0887 22094911

[156] Chen F , Maccubbin D, Yan L, Sirah W, Chen E, Sisk CM, et al Lipid-altering efficacy and safety profile of co-administered extended-release niacin/laropiprant and simvastatin versus atorvastatin in patients with mixed hyperlipidemia. Int J Cardiol 2013;167:225–231. 22305632 10.1016/j.ijcard.2011.12.103

[157] Kim SH , Seo MK, Yoon MH, Choi DH, Hong TJ, Kim HS. Assessment of the efficacy and tolerability of 2 formulations of atorvastatin in Korean adults with hypercholesterolemia: a multicenter, prospective, open-label, randomized trial. Clin Ther 2013;35:77–86. 23274145 10.1016/j.clinthera.2012.11.009

[158] Lee JH , Kang HJ, Kim HS, Sohn DW, Oh BH, Park YB. Effects of ezetimibe/simvastatin 10/20 mg vs. atorvastatin 20 mg on apolipoprotein B/apolipoprotein A1 in Korean patients with type 2 diabetes mellitus: results of a randomized controlled trial. Am J Cardiovasc Drugs 2013;13:343–351. 23728830 10.1007/s40256-013-0031-6

[159] Liu PY , Lin LY, Lin HJ, Hsia CH, Hung YR, Yeh HI, et al Pitavastatin and Atorvastatin double-blind randomized comPArative study among hiGh-risk patients, including thOse with Type 2 diabetes mellitus, in Taiwan (PAPAGO-T Study). PLoS One 2013;8:e76298. 24098467 10.1371/journal.pone.0076298 PMC3788128

[160] Sasaki J , Otonari T, Uchida Y, Ikeda Y, Biro S, Kono S, et al Effects of pravastatin and atorvastatin on HDL cholesterol and glucose metabolism in patients with dyslipidemia and glucose intolerance: the PRAT study. J Atheroscler Thromb 2013;20:368–379. 23257975 10.5551/jat.13532

[161] Zhang H , Plutzky J, Skentzos S, Morrison F, Mar P, Shubina M, et al Discontinuation of statins in routine care settings: a cohort study. Ann Intern Med 2013;158:526–534. 23546564 10.7326/0003-4819-158-7-201304020-00004 PMC3692286

[162] Rosenbaum D , Dallongeville J, Sabouret P, Bruckert E. Discontinuation of statin therapy due to muscular side effects: a survey in real life. Nutr Metab Cardiovasc Dis 2013;23:871–875. 22748604 10.1016/j.numecd.2012.04.012

[163] Parker BA , Capizzi JA, Grimaldi AS, Clarkson PM, Cole SM, Keadle J, et al Effect of statins on skeletal muscle function. Circulation 2013;127:96–103. 23183941 10.1161/CIRCULATIONAHA.112.136101 PMC4450764

[164] Chang CH , Kusama M, Ono S, Sugiyama Y, Orii T, Akazawa M. Assessment of statin-associated muscle toxicity in Japan: a cohort study conducted using claims database and laboratory information. BMJ Open 2013;3:e002040. 10.1136/bmjopen-2012-002040 PMC3641424 23585384

[165] Mampuya WM , Frid D, Rocco M, Huang J, Brennan DM, Hazen SL, et al Treatment strategies in patients with statin intolerance: the Cleveland Clinic experience. Am Heart J 2013;166:597–603. 24016512 10.1016/j.ahj.2013.06.004 PMC4038261

[166] Robison CD , Bair TL, Horne BD, McCubrey RO, Lappe DL, Muhlestein JB, et al Hypothyroidism as a risk factor for statin intolerance. J Clin Lipidol 2014;8:401–407. 25110221 10.1016/j.jacl.2014.05.005

[167] Ito MK , Maki KC, Brinton EA, Cohen JD, Jacobson TA. Muscle symptoms in statin users, associations with cytochrome P450, and membrane transporter inhibitor use: a subanalysis of the USAGE study. J Clin Lipidol 2014;8:69–76. 24528687 10.1016/j.jacl.2013.10.006

[168] Quek RG , Fox KM, Wang L, Li L, Gandra SR, Wong ND. Lipid-lowering treatment patterns among patients with type 2 diabetes mellitus with high cardiovascular disease risk. BMJ Open Diabetes Res Care 2015;23:e000132. 10.1136/bmjdrc-2015-000132 PMC4586941 26435839

[169] Svensson E , Beck Nielsen R, Hasvold P, Aarskog P, Thomsen RW. Statin prescription patterns, adherence, and attainment of cholesterol treatment goals in routine clinical care: a Danish population based study. Clin Epidemiol 2015;7:213–223. 25759601 10.2147/CLEP.S78145 PMC4345937

[170] Izawa A , Kashima Y, Miura T, Ebisawa S, Kitabayashi H, Yamamoto H, et al Assessment of lipophilic vs. hydrophilic statin therapy in acute myocardial infarction – ALPS-AMI study. Circ J 2015;79:161–168. 25392071 10.1253/circj.CJ-14-0877

[171] Vinogradova Y , Coupland C, Brindle P, Hippisley-Cox J. Discontinuation and restarting in patients on statin treatment: prospective open cohort study using a primary care database. BMJ 2016;353:i3305. 27353261 10.1136/bmj.i3305 PMC4925919

[172] Schulman KL , Lamerato LE, Dalal MR, Sung J, Jhaveri M, Koren A, et al Development and validation of algorithms to identify statin intolerance in a US administrative database. Value Health 2016;19:852–860. 27712714 10.1016/j.jval.2016.03.1858

[173] Colantonio LD , Kent ST, Huang L, Chen L, Monda KL, Serban MC, et al Algorithms to identify statin intolerance in medicare administrative claim data. Cardiovasc Drugs Ther 2016;30:525–533. 27389413 10.1007/s10557-016-6680-3

[174] Halava H , Huupponen R, Pentti J, Kivim.ki M, Vahtera J. Predictors of first-year statin medication discontinuation: a cohort study. J Clin Lipidol 2016;10:987–995. 27578131 10.1016/j.jacl.2016.04.010 PMC5012887

[175] Yusuf S , Bosch J, Dagenais G, Zhu J, Xavier D, Liu L, et al Cholesterol lowering in intermediate-risk persons without cardiovascular disease. N Engl J Med 2016;374:2021–2031. 27040132 10.1056/NEJMoa1600176

[176] Serban MC , Colantonio LD, Manthripragada AD, Monda KL, Bittner VA, Banach M, et al Statin intolerance and risk of coronary heart events and all-cause mortality following myocardial infarction. J Am Coll Cardiol 2017;69:1386–1395. 28302290 10.1016/j.jacc.2016.12.036

[177] Brinton EA . Understanding patient adherence and concerns with STatins and MedicatION Discussions With Physicians (ACTION): a survey on the patient perspective of dialogue with healthcare providers regarding statin therapy. Clin Cardiol 2018;41:710–720. 29749101 10.1002/clc.22975 PMC6490009

[178] Ihle P , Dippel FW, Schubert I. Statin-associated myopathy. Assessment of frequency based on data of all statutory health insurance funds in Germany. Pharmacol Res Perspect 2018;6:e00404. 29760929 10.1002/prp2.404 PMC5943670

[179] Chee WJ , Abdullahi H, Chan Y, Rattle A, Snedden S, Junckerstorff R. Retrospective evaluation of statin prescription in the elderly. Intern Med J 2018;48:1463–1471. 29869449 10.1111/imj.13996

[180] Nagar SP , Rane PP, Fox KM, Meyers J, Davis K, Beaubrun A, et al Treatment patterns, statin intolerance, and subsequent cardiovascular events among Japanese patients with high cardiovascular risk initiating statin therapy. Circ J 2018;82:1008–1016. 29276211 10.1253/circj.CJ-17-0811

[181] van Delden XM , Huijgen R, Wolmarans KH, Brice BC, Barron JK, Blom DJ, et al LDL-cholesterol target achievement in patients with heterozygous familial hypercholesterolemia at Groote Schuur Hospital: minority at target despite large reductions in LDL-C. Atherosclerosis 2018;277:327–333. 30270067 10.1016/j.atherosclerosis.2018.06.820

[182] Ofori-Asenso R , Ilomäki J, Tacey M, Zomer E, Curtis AJ, Bell JS, et al Patterns of statin use and long-term adherence and persistence among older adults with diabetes. J Diabetes 2018;10:699–707. 29658177 10.1111/1753-0407.12769

[183] Mefford MT , Tajeu GS, Tanner RM, Colantonio LD, Monda KL, Dent R, et al Willingness to be reinitiated on a statin (from the REasons for Geographic and Racial Differences in Stroke Study). Am J Cardiol 2018;122:768–774. 30057227 10.1016/j.amjcard.2018.05.016 PMC9581445

[184] Kajinami K , Ozaki A, Tajima Y, Yamashita S, Arai H, Teramoto T. Real-world data to identify hypercholesterolemia patients on suboptimal statin therapy. J Atheroscler Thromb 2019;26:408–431. 30369517 10.5551/jat.46201 PMC6514177

[185] Chen WJ , Wen YC, Fox KM, Shen LJ, Lin LY, Qian Y, et al Treatment patterns of lipid-lowering therapies and possible statin intolerance among statin users with clinical atherosclerotic cardiovascular disease (ASCVD) or diabetes mellitus (DM) in Taiwan. J Eval Clin Pract 2020;26:1171–1180. 31646715 10.1111/jep.13286

[186] Ofori-Asenso R , Ilomäki J, Tacey M, Si S, Curtis AJ, Zomer E, et al Predictors of first-year nonadherence and discontinuation of statins among older adults: a retrospective cohort study. Br J Clin Pharmacol 2019;85:227–235. 30402916 10.1111/bcp.13797 PMC6303220

[187] Roh JW , Chun KH, Kang M, Lee CJ, Oh J, Shim CY, et al PRavastatin versus FlUVastatin after statin intolerance: the PRUV-intolerance study with propensity score matching. Am J Med 2019;132:1320–1326.e1. 31278931 10.1016/j.amjmed.2019.06.003

[188] Bradley CK , Wang TY, Li S, Robinson JG, Roger VL, Goldberg AC, et al Patient-reported reasons for declining or discontinuing statin therapy: insights from the PALM registry. J Am Heart Assoc 2019;8:e011765. 30913959 10.1161/JAHA.118.011765 PMC6509731

[189] Jacobson TA , Cheeley MK, Jones PH, La Forge R, Maki KC, López JAG, et al The STatin adverse treatment experience survey: experience of patients reporting side effects of statin therapy. J Clin Lipidol 2019;13:415–424. 31113745 10.1016/j.jacl.2019.04.011

[190] Bair TL , May HT, Knowlton KU, Anderson JL, Lappe DL, Muhlestein JB. Predictors of statin intolerance in patients with a new diagnosis of atherosclerotic cardiovascular disease within a large integrated health care institution: the IMPRES study. J Cardiovasc Pharmacol 2020;75:426–431. 32079856 10.1097/FJC.0000000000000808

[191] Casula M , Gazzotti M, Bonaiti F, OImastroni E, Arca M, Averna M, et al Reported muscle symptoms during statin treatment amongst Italian dyslipidaemic patients in the real-life setting: the PROSISA Study. J Intern Med 2021;290:116–128. 33259671 10.1111/joim.13219 PMC8359216

[192] Yao X , Shah ND, Gersh BJ, Lopez-Jimenez F, Noseworthy PA. Assessment of trends in statin therapy for secondary prevention of atherosclerotic cardiovascular disease in US adults from 2007 to 2016. JAMA Netw Open 2020;3:e2025505. 33216139 10.1001/jamanetworkopen.2020.25505 PMC7679951

[193] Thompson W , Jarbøl DE, Nielsen JB, Haastrup P, Pottegård A. Statin use and discontinuation in Danes age 70 and older: a nationwide drug utilisation study. Age Ageing 2021;50:554–558. 32936863 10.1093/ageing/afaa160

[194] Moore JL , McFarland GE, Novak Z, Patterson MA, Haverstock B, Passman MA, et al Effects of statin and antiplatelet therapy noncompliance and intolerance on patient outcomes following vascular surgery. J Vasc Surg 2020;71:1358–1369. 32035776 10.1016/j.jvs.2019.07.063

[195] De Vera M , Bhole V, Burns L, Lacaille D. Impact of statin adherence on cardiovascular disease and mortality outcomes: a systematic review. Br J Clin Pharmacol 2014;78:684–698. 25364801 10.1111/bcp.12339 PMC4239963

[196] Gomez Sandoval YH , Braganza MV, Daskalopoulou SS. Statin discontinuation in high-risk patients: a systematic review of the evidence. Curr Pharm Des 2011;17:3669–3689. 22074437 10.2174/138161211798220891

[197] Nissen SE , Stroes E, Dent-Acosta RE, Rosenson RS, Lehman SJ, Sattar N, et al Efficacy and tolerability of evolocumab vs ezetimibe in patients with muscle-related statin intolerance: the GAUSS-3 randomized clinical trial. JAMA 2016;315:1580–1590. 27039291 10.1001/jama.2016.3608

[198] Moriarty PM , Thompson PD, Cannon CP, Guyton JR, Bergeron J, Zieve FJ, et al Efficacy and safety of alirocumab vs ezetimibe in statin-intolerant patients, with a statin rechallenge arm: the ODYSSEY ALTERNATIVE randomized trial. J Clin Lipidol 2015;9:758–769. 26687696 10.1016/j.jacl.2015.08.006

[199] Penson PE , Banach M. Nocebo/drucebo effect in statin-intolerant patients: an attempt at recommendations. Eur Heart J 2021;42:4787–4788. 34151941 10.1093/eurheartj/ehab358

[200] Bytyçi I , Bajraktari G, Bhatt DL, Morgan CJ, Ahmed A, Aronow WS, *et al*. Hydrophilic vs lipophilic statins in coronary artery disease: a meta-analysis of randomized controlled trials. J Clin Lipidol 2017;11:624–637. 28506385 10.1016/j.jacl.2017.03.003

[201] Pasternak RC , Smith SC Jr, Bairey-Merz CN, Grundy SM, Cleeman JI, Lenfant C, et al ACC/AHA/NHLBI clinical advisory on the use and safety of statins. Circulation 2002;106:1024–1028. 12186811 10.1161/01.cir.0000032466.44170.44

[202] Penson PE , Mancini GBJ, Toth PP, Martin SS, Watts GF, Sahebkar A, et al Introducing the ‘Drucebo’ effect in statin therapy: a systematic review of studies comparing reported rates of statin-associated muscle symptoms, under blinded and open-label conditions. J Cachexia Sarcopenia Muscle 2018;9:1023–1033. 30311434 10.1002/jcsm.12344 PMC6240752

[203] Banach M , Penson PE. Drucebo effect—the challenge we should all definitely face! Arch Med Sci 2021;17:542–543. 33747289 10.5114/aoms/132304 PMC7959055

[204] Bell RA , Franks P, Duberstein PR, Epstein RM, Feldman MD, Fernandez Y, et al Suffering in silence: reasons for not disclosing depression in primary care. Ann Fam Med 2011;9:439–446. 21911763 10.1370/afm.1277 PMC3185469

[205] Catapano AL , Graham I, De Backer G, Wiklund O, Chapman MJ, Drexel H, et al 2016 ESC/EAS guidelines for the management of dyslipidaemias: the Task Force for the Management of Dyslipidaemias of the European Society of Cardiology (ESC) and European Atherosclerosis Society (EAS) developed with the special contribution of the European Association for Cardiovascular Prevention & Rehabilitation (EACPR). Atherosclerosis 2016;253:281–344. 27594540 10.1016/j.atherosclerosis.2016.08.018

[206] Newman CB , Preiss D, Tobert JA, Jacobson TA, Page RL II, Goldstein LB, et al Statin safety and associated adverse events: a scientific statement from the American Heart Association. Arterioscler Thromb Vasc Biol 2019;39:e38–e81. 30580575 10.1161/ATV.0000000000000073

