# Supplementary material for: Prevalence of statin intolerance: a meta-analysis
Source: Eur Heart J. 2022 Feb 16;43(34):3213–23. doi: 10.1093/eurheartj/ehac015 (PMC9757867; doi:10.1093/eurheartj/ehac015)
Supplement: ehac015_Supplementary_Data [file ehac015_supplementary_data.zip › SI_SUPPLEMENTAL-MATERIAL_EHJ_Revised_Final_09122021_1.docx]

**SUPPLEMENTAL MATERIAL**

**­­­­­­­­­––––––––––––––––––––––––––––––––––––––––––––––––––––––––––––––––––––––––**

**Prevalence of statin intolerance: A systematic review and meta-analysis**

Contents

[Table S1. PECOS model 2](#_Toc67823651)

[Table S2. The search and screening strategy. 2](#_Toc67823652)

[Table S3. Main characteristics of papers included in the study. 3-10](#_Toc67823653)

[Table S4. The list of studies that were excluded from the meta-analysis due to data overlapping…. 11-15](#_Toc67823653)

[Figure S1. Defination of statin intolerance. 16](#_Toc67823653)

[Figure S2. Pooled prevalence of SI. 17](#_Toc67823654)

[Figure S3. Prevalence of SI based on NLA criteria. 18](#_Toc67823655)

[Figure S4. Prevalence of SI based on ESA criteria. 19](#_Toc67823656)

[Figure S5. Prevalence of SI based on ILEP criteria. 20](#_Toc67823657)

[Figure S6. Prevalence of SI on RCT studies . 21](#_Toc67823658)

[Figure S6. Prevalence of SI on RCT studies . 22](#_Toc67823658)

[Figure S7. Prevalence of SI on Cohort studies. 23](#_Toc67823659)

[Figure S8. Prevalence of SI on different disease in the primary prevention. 24](#_Toc67823663)

[Figure S9. Prevalence of SI on different disease in the secondary prevention. 25](#_Toc67823664)

[Figure 10. Prevalence of SI on different disease from hydrphilic statins. 26](#_Toc67823664)

[Figure S11. Prevalence of SI on different disease from lipophilic statins. 27](#_Toc67823664)

[Figure S12. Meta-regression of demographic indices on SI. 28](#_Toc67823665)

[Figure S13. Meta-regression of risk factors on SI. 29](#_Toc67823666)

[Figure S14. Meta-regression of risk factors and drugs on SI. 30](#_Toc67823667)

[Table S5. Assessment of risk of bias in the included studies using Cochrance criteri for RCTs….30-34](#_Toc67823653)

[Table S6. Assessment of risk of bias in the included studies using NOS for cohort studies 35-37](#_Toc67823653)

**Table S1.** PECOS model

| **PECOS model** | |
| --- | --- |
| **Participants:** | Participants using statin for primary or secondary prevention |
| **Exposure:** | Use of statin in different disease settings. Primary prevention: primary hypercholesterolemia, |
|  | hypercholesterolemia, dyslipidemia, diabetes mellitus and secondary prevention: coronary artery |
|  | disease (stable coronary artery disease, acute coronary syndrome, myocardial infarction), stroke/TIA |
| **Comparator/Control:** | Not applicable |
| **Outcomes:** | Primary outcomes: overall prevalence and prevalence based on different diagnostic criteria: National |
|  | Lipid Association/NLA, International Lipid Expert Panel/ILEP and European Atherosclerosis Society/EAS. |
|  | Secondary outcome: Identify the predictors of statin intolerance. |
| **Study design:** | Previously systematic reviews or meta-analysis were not included but these articles were searched |
|  | for individual studies |

**Table S2.** The search and screening strategy

| **Electronic data** | PubMed-Medline, EMBASE, Scopus, Google Scholar, the Cochrane Central Registry of Controlled |
| --- | --- |
|  | Trials and ClinicalTrial.gov |
| **Statin intolerance** | "Statin intolerance" OR "Statin Toxicity" OR "Statin adverse effects" OR "Statin side effects" OR |
| **term** | "Statin-associated muscle symptoms" OR "SAMS" OR "statin-related myopathy" OR "statin-related |
|  | side effects" OR "statin-related myalgia" OR "Statin discontinuation" OR "Statin withdrawal" |
|  | AND |
| **Occurrence term** | "Prevalence" OR "Occurrence rate" OR "Frequency rate" |
| **Additional search** | Scientific sessions: American Heart Association (AHA), American College of Cardiology (ACC), |
|  | National Lipid Association (NLA), European Society of Atherosclerosis (EAS) |

**Table S3.** Main characteristics of papers included in the study

| **Study name** | **Design** | **Prevention** | **Sample** | **Age** | **Female** | **Prevalence** | **Diagnostic criteria** | | |
| --- | --- | --- | --- | --- | --- | --- | --- | --- | --- |
|  |  | **Disease** | **size (n)** | **(year)** | **(%)** |  | **NLA** | **ILEP** | **EAS** |
| Kannel 1990 | Cohort study | Primary | 489 | NR | 48.3 | 0.04 | 0.04 | 0.04 | NR |
| Bradford 1991 | RCTs | Primary | 1663 | 55±4 | 41 | 0.04 | 0.04 | 0.04 | NR |
| Crepaldi 1991 | RCTs | Primary | 193 | NR | NR | 0.16 | 0.16 | 0.16 | NR |
| PMS group 1993 | RCTs | Primary | 530 | 55±5.2 | 23 | 0.05 | 0.05 | 0.05 | NR |
| PMS Group II 1993 | RCTs | Primary | 190 | 52.8±3.4 | 30.5 | 0.13 | 0.13 | 0.13 | 0.04 |
| Blankenhorn 1993 | RCTs | Secondary | 123 | 58±4.1 | 9.0 | 0.05 | 0.05 | 0.05 | 0.03 |
| Wiklund 1993 | RCTs | Primary | 129 | 52.5 ±1.3 | 34.3 | 0.12 | 0.12 | 0.12 | 0.09 |
| Furberg 1994 | RCTs | Primary | 231 | 61±9 | 50 | 0.04 | 0.04 | NR | NR |
| Keech 1994 | RCTs | Primary | 208 | 63 ±3.7 | 15 | 0.09 | 0.09 | 0.09 | NR |
| Insull 1994 | RCTs | Primary | 138 | 54 ±11 | 30.4 | 0.00 | 0.00 | 0.00 | NR |
| Jacotot 1994 | RCTs | Primary | 344 | 48 ± 12 | 47 | 0.00 | 0.00 | 0.00 | NR |
| Salonen 1994 | RCTs | Secondary | 224 | 57.3±6.2 | NR | 0.03 | 0.03 | 0.03 | NR |
| Shepherd 1995 | RCTs | Primary | 3302 | 55.3 ±5.5 | NR | 0.05 | 0.05 | 0.05 | NR |
| Andrade 1995 | Cohort study | Primary | 537 | >70 | 53.1 | 0.07 | 0.07 | 0.07 | NR |
| Pitt 1995 | RCTs | Secondary | 206 | NR | 21 | 0.00 | 0.00 | 0.00 | NR |
| Jukema 1995 | RCTs | Secondary | 392 | 56.5 ± 8 | NR | 0.03 | 0.03 | 0.03 | NR |
| Simons 1996 | Cohort study | Mixed | 610 | >70 | 51 | 0.04 | 0.04 | NR | NR |
| Sacks, 1996 | RCTs | Secondary | 2081 | 59 ± 9 | 14 | 0.02 | 0.02 | 0.02 | 0.01 |
| Bertolini, 1997 | RCTs | Primary | 305 | 57 ± 1.2 | 55 | 0.03 | 0.03 | 0.02 | NR |
| Dart 1997 | RCTs | Primary | 177 | NR | NR | 0.07 | 0.07 | 0.07 | 0.04 |
| Davidson 1997 | RCTs | Primary | 1049 | 57 ± 0.4 | 42 | 0.03 | 0.03 | NR | NR |

*Continued table*

| Herd 1997 | RCTs | Secondary | 214 | NR | 23 | 0.03 | 0.00 | NR | NR |
| --- | --- | --- | --- | --- | --- | --- | --- | --- | --- |
| Jones 1998 | RCTs | Primary | 518 | 55±1.2 | 41 | 0.02 | 0.02 | NR | NR |
| Tonkin 1998 | RCTs | Secondary | 4512 | >70 | 15 | 0.03 | 0.03 | 0.03 | NR |
| Downs 1998 | RCTs | Primary | 3304 | 58 ± 7 | NR | 0.14 | 0.14 | 0.14 | 0.02 |
| Eriksson 1998 | RCTs | Primary | 1541 | 53.7 ± 7.8 | NR | 0.13 | 0.13 | 0.13 | NR |
| Hiatt 1999 | Cohort study | Primary | 970 | 64 ± 11 | 7 | 0.07 | NR | NR | NR |
| Bruckert 1999 | RCTs | Primary | 2888 | 56.8 ± 11 | 41 | 0.10 | NR | NR | NR |
| Serruys 1999 | RCTs | Secondary | 526 | 60 ± 9 | 17 | 0.06 | NR | NR | NR |
| März 1999 | RCTs | Secondary | 2856 | 60.5 ± 8.6 | 63 | 0.04 | NR | NR | NR |
| Barter 2000 | RCTs | Primary | 1028 | 56.5±3.3 | 48 | 0.08 | 0.08 | 0.08 | NR |
| Gentile 2000 | RCTs | Primary | 412 | 58 ± 6 | 27 | 0.01 | 0.01 | 0.01 | NR |
| Schwartz 2001 | RCTs | Secondary | 1538 | 65 ± 12 | 35.5 | 0.03 | 0.03 | 0.03 | 0.00 |
| Olsson 2001 | RCTs | Primary | 189 | 56±2.8 | NR | 0.02 | 0.02 | NR | NR |
| Smilde 2001 | RCTs | Primary | 280 | NR | NR | 0.03 | 0.03 | 0.03 | NR |
| Andrews 2001 | RCTs | Primary | 3785 | 62 ± 11 | 38 | 0.07 | 0.07 | 0.07 | NR |
| Insull 2001 | RCTs | Primary | 1424 | 60.9±8.9 | 47 | 0.02 | 0.02 | 0.02 | 0.03 |
| Branchi 2001 | RCTs | Primary | 235 | 56.7±6.4 | 55 | 0.04 | 0.04 | 0.04 | 0.00 |
| Illingworth 2001 | RCTs | Primary | 813 | NR | 45 | 0.02 | 0.02 | 0.02 | 0.00 |
| DALI study 2001 | RCTs | Primary | 145 | 59.7 ± 76 | 43 | 0.01 | 0.01 | 0.01 | 0.02 |
| Hunninghake 2001 | RCTs | Primary | 115 | 56.5±3.2 | 41 | 0.03 | 0.03 | 0.03 | NR |
| Saito 2002 | RCTs | Primary | 2529 | NR | NR | 0.02 | 0.02 | 0.02 | NR |
| Jackevicius 2002 | Cohort study | Primary & S | 144504 | 72.5 ± 5 | 43 | 0.39 | NR | NR | 0.01 |
| Benner 2002 | Cohort study | Mixed | 34501 | 74.4 ± 6.2 | 79.9 | 0.29 | NR | NR | NR |
| Larsen 2002 | Cohort study | Mixed | 3623 | NR | NR | 0.14 | NR | NR | NR |

*Continued table*

| HPC Group 2002 | RCTs | Secondary | 10269 | NR | NR | 0.05 | 0.05 | 0.05 | 0.00 |
| --- | --- | --- | --- | --- | --- | --- | --- | --- | --- |
| Shepherd 2002 | RCTs | Primary | 2891 | 75.3±3.4 | 52 | 0.04 | 0.04 | 0.04 | 0.01 |
| Serruys 2002 | RCTs | Secondary | 844 | 75.3±3.4 | 16 | 0.09 | NR | NR | NR |
| Wei 2002 | Cohort study | Secondary | 427 | >70 | 7 | 0.16 | NR | NR | NR |
| Karalis 2002 | RCTs | Mixed | 1694 | 61.5±3.8 | 39 | 0.02 | 0.02 | NR | 0.00 |
| Matsuzaki 2002 | Cohort study | Primary | 47,294 | 58.9 ± 7.6 | 70 | 0.02 | 0.02 | NR | NR |
| Mohler 2003 | RCTs | Secondary | 354 | 69±9.8 | 23 | 0.03 | NR | NR | NR |
| Bruckert 2003 | RCTs | Primary | 607 | >80 | 74.8 | 0.03 | NR | NR | 0.01 |
| Ballantyne 2003 | RCTs | Primary | 503 | NR | 44 | 0.06 | 0.06 | 0.06 | 0.04 |
| Kerzner 2003 | RCTs | primary | 412 | 56 ± 12 | 57 | 0.05 | NR | NR | 0.04 |
| Rosenson 2003 | RCTs | primary | 594 | NR | NR | 0.08 | 0.08 | 0.08 | 0.04 |
| Yang 2003 | Cohort study | Mixed | 12167 | >80 | 47 | 0.19 | NR | NR | NR |
| Abraha 2003 | Cohort study | Mixed | 39,222 | 62.9±8.7 | 54.6 | 0.13 | NR | NR | NR |
| Holdaas 2003 | RCTs | Mixed | 1045 | 49.5± 10 | 33 | 0.31 | NR | NR | 0.01 |
| Olsson 2003 | RCTs | Primary | 1093 | 63 ± 8 | 24 | 0.03 | 0.03 | 0.03 | 0.02 |
| Ballantyne 2003 | RCTs | Primary | 917 | NR | 43 | 0.07 | 0.07 | 0.07 | 0.07 |
| Schneck 2003 | RCTs | Primary | 374 | 56.3 ± 12 | 49 | 0.02 | 0.02 | 0.02 | 0.02 |
| Stein 2003 | RCTs | Primary | 623 | 47.5 ± 13 | 45 | 0.03 | 0.03 | 0.03 | 0.10 |
| Schuster 2004 | RCTs | Primary | 3134 | 62.2 ± 10.2 | 44 | 0.01 | 0.01 | 0.01 | 0.01 |
| Schwartz 2004 | RCTs | Mixed | 382 | 62±6.7 | 52 | 0.05 | 0.05 | 0.05 | 0.02 |
| Beishuizen 2004 | RCTs | Primary | 125 | 58.8 ± 1.3 | 51 | 0.14 | 0.14 | 0.14 | 0.14 |
| Hunninghake 2004 | RCTs | Primary | 130 | 56 ± 10 | 40 | 0.02 | 0.02 | 0.02 | 0.05 |
| Goldberg 2004 | RCTs | Primary | 702 | NR | 51.5 | 0.02 | 0.02 | 0.02 | NR |
| Ellis 2004 | Cohort study | Primary | 5189 | 51 ± 13 | 45 | 0.19 | NR | NR | NR |

*Continued table*

| Cannon 2004 | RCTs | Secondary | 4162 | 58 ± 11.3 | 22 | 0.03 | 0.03 | 0.03 | 0.02 |
| --- | --- | --- | --- | --- | --- | --- | --- | --- | --- |
| Nissen 2004 | RCTs | Secondary | 654 | 58 ±9.2 | 28 | 0.04 | 0.04 | 0.04 | 0.03 |
| Benner 2004 | Cohort | Mixed | 19422 | NR | 50 | 0.31 | 0.31 | NR | NR |
| Eagle 2004 | Cohort study | Secondary | 21,408 | 65±7.5 | 32.5 | 0.13 | 0.13 | NR | NR |
| Howell 2004 | Cohort study | Primary | 869 | NR | NR | 0.14 | 0.14 | NR | NR |
| Koren 2004 | RCTs | Secondary | 1,217 | 61 ± 9 | 17.8 | 0.02 | 0.02 | 0.02 | NR |
| [Colhoun 2004](https://pubmed.ncbi.nlm.nih.gov/?term=Colhoun+HM&cauthor_id=15325833) | RCTs | Primary | 1428 | 62 ± 8.3 | 32 | 0.09 | 0.09 | 0.09 | 0.04 |
| SSS Group | RCTs | Secondary | 2221 | 59.5 ± 6.2 | 18 | 0.06 | 0.06 | 0.06 | NR |
| De Lemos 2004 | RCTs | Secondary | 4497 | 61±5.5 | 24 | 0.02 | 0.02 | 0.02 | 0.02 |
| ADRAC 2004 | RCTs | Mixed | 4238 | NR | NR | 0.22 | 0.22 | 0.22 | 0.22 |
| Bruckert 2005 | Cohort study | Primary | 7092 | 58.5 ±10 | 65 | 0.02 | 0.02 | 0.02 | 0.02 |
| Caspard, 2005 | Cohort study | Primary | 4776 | NR | 48 | 0.20 | NR | NR | NR |
| Perreault 2005 | Cohort | Primary | 17958 | 57±8.4 | 50 | 0.33 | NR | NR | NR |
| Blackburn 2005 | Cohort | Secondary | 1221 | 59±8.7 | 24 | 0.32 | NR | NR | NR |
| La Rosa 2005 | RCTs | Secondary | 10,001 | 61±8 | 19 | 0.06 | 0.06 | 0.06 | 0.05 |
| Pedersen 2005 | RCTs | Secondary | 8888 | 62±9.5 | 19 | 0.03 | 0.03 | 0.03 | 0.02 |
| Amarenco 2006 | RCTs | Secondary | 2365 | 63±0.2 | 40 | 0.06 | 0.06 | 0.06 | 0.06 |
| KNOPP 2006 | RCTs | Secondary | 1211 | 62±8.1 | 34 | 0.05 | 0.05 | 0.05 | 0.03 |
| Nakamura 2006 | RCTs | Primary | 3866 | 58±7.3 | 68 | 0.04 | 0.04 | 0.04 | NR |
| Nissen 2006 | RCTs | Secondary | 507 | 58.5±10 | 30 | 0.05 | 0.05 | 0.05 | 0.04 |
| Save 2006 | Cohort study | Primary | 110 | 51.3±8.6 | 71 | 0.00 | 0.00 | 0.00 | 0.00 |
| Goldberg 2006 | RCTs | Primary | 494 | 59.1±10 | 55.1 | 0.02 | 0.02 | NR | NR |
| Binbrek 2006 | RCTs | Primary | 1497 | 58.4±10.9 | 51 | 0.02 | 0.02 | 0.02 | 0.01 |
| Clearfield 2006 | RCTs | Secondary | 954 | 60.2±10 | 46 | 0.02 | 0.02 | 0.02 | 0.02 |

*Continued table*

| Betteridge 2007 | RCTs | Primary | 494 | 61±10 | 22 | 0.06 | 0.06 | 0.06 | 0.05 |
| --- | --- | --- | --- | --- | --- | --- | --- | --- | --- |
| Blagden 2007 | RCTs | Secondary | 148 | 65±9.4 | 28 | 0.05 | 0.05 | 0.05 | NR |
| Lee 2007 | RCTs | Secondary | 122 | 59±8 | 50 | 0.04 | 0.04 | 0.04 | 0.01 |
| McGinnis 2007 | Cohort study | Mixed | 34735 | 60±4.4 | 32.5 | 0.19 | 0.19 | 0.19 | NR |
| Hudson 2007 | Cohort study | Secondary | 381 | 71±11 | NR | 0.24 | NR | NR | NR |
| Kamal-Bahl 2007 | Cohort study | Mixed | 46846 | >65 | 17.8 | 0.29 | NR | NR | NR |
| Kjekshus 2007 | RCTs | Primary | 2514 | 73±7 | 32 | 0.10 | 0.10 | 0.10 | 0.09 |
| Crouse JR 3rd 2007 | RCTs | Primary | 700 | 57±6.2 | 18 | 0.13 | 0.13 | 0.13 | 0.13 |
| Leiter 2007 | RCTs | Secondary | 871 | 62.6±8.5 | 24 | 0.04 | 0.04 | 0.04 | NR |
| Deedwinia 2007 | RCTs | Secondary | 891 | 72±10 | NR | 0.11 | 0.11 | 0.11 | 0.03 |
| Yu 2008 | Cohort study | Mixed | 19038 | 58.2±11 | 65 | 0.29 | NR | NR | NR |
| Donnelly 2008 | Cohort study | Primary | 6462 | 62.8±11 | 48 | 0.13 | NR | NR | NR |
| Chodick 2008 | Cohort study | Primary | 136052 | 58.5±6.2 | 50 | 0.31 | NR | NR | NR |
| Helin-Salmivaara | Cohort study | Mixed | 18072 | NR | 24 | 0.26 | NR | NR | NR |
| 2008 |  |  |  |  |  |  |  |  |  |
| Tavazzi 2008 | RCTs | Primary | 2314 | 68±11 | 19 | 0.04 | 0.04 | 0.04 | 0.01 |
| Ridker 2008 | RCTs | Primary | 8901 | 66±7.1 | 19 | 0.05 | 0.05 | NR | 0.03 |
| Newman 2008 | RCTs | Primary | 1428 | NR | 40 | 0.09 | 0.09 | 0.09 | 0.02 |
| Bonnet 2008 | RCTs | Secondary | 239 | 62.9±8.9 | 34 | 0.04 | 0.04 | 0.04 | 0.03 |
| Conard 2008 | RCTs | Primary | 196 | 58±10 | 68 | 0.01 | 0.01 | 0.01 | NR |
| Abate 2008 | RCTs | Primary | 6967 | 59.9±9.3 | 30 | 0.00 | 0.00 | 0.00 | NR |
| Yokote 2008 | RCTs | Primary | 204 | 62±11 | 71 | 0.05 | 0.05 | 0.05 | 0.02 |
| Sasaki 2008 | RCTs | Primary | 173 | 64±9.5 | 55.1 | 0.07 | 0.07 | 0.07 | 0.02 |
| Yamazaki 2009 | RCTs | Primary | 900 | >60 | 51 | 0.09 | 0.09 | 0.09 | 0.02 |
| Budinski 2009 | RCTs | Primary | 821 | 58±9.1 | 46 | 0.01 | 0.01 | 0.01 | 0.01 |

*Continued table*

| Davidson 2009 | RCTs | Primary | 147 | 57±9.9 | 52.7 | 0.06 | 0.06 | 0.06 | 0.05 |
| --- | --- | --- | --- | --- | --- | --- | --- | --- | --- |
| Insull 2009 | RCTs | Primary | 193 | 52±11 | 45 | 0.14 | 0.14 | 0.14 | 0.04 |
| Fellström 2009 | RCTs | Primary | 1389 | 64±8.6 | 38.7 | 0.01 | 0.01 | 0.01 | NR |
| Hall 2009 | RCTs | Secondary | 1263 | 62±11 | 21 | 0.02 | 0.02 | 0.02 | NR |
| Corrao 2010 | Cohort study | Secondary | 90,832 | 62±12 | 59.7 | 0.35 | NR | NR | NR |
| Lablanche 2010 | RCTs | Secondary | 887 | 60±11 | 26 | 0.03 | 0.03 | 0.03 | 0.01 |
| Kim 2010 | RCTs | Secondary | 235 | 61±3 | 48.7 | 0.05 | 0.05 | 0.05 | 0.02 |
| Park 2010 | RCTs | Primary | 350 | 60±075 | 40 | 0.00 | 0.00 | 0.00 | NR |
| Armitage 2010 | RCTs | Secondary | 12064 | NR | NR | 0.02 | 0.02 | 0.02 | 0.01 |
| Ose 2010 | RCTs | Primary | 1353 | NR | NR | 0.04 | 0.04 | 0.04 | 0.01 |
| Gumprecht 2011 | RCTs | Primary | 412 | 59.8±9 | 57 | 0.02 | 0.02 | 0.02 | 0.02 |
| Albert 2011 | RCTs | Primary | 17,802 | 66±4.5 | 46 | 0.07 | NR | NR | NR |
| Geers 2011 | Cohort study | Mixed | 6615 | NR | NR | 0.21 | NR | NR | NR |
| Harris 2011 | Cohort study | Mixed | 418 | 64±8 | 8 | 0.25 | 0.25 | 0.25 | 0.23 |
| Nicholls 2011 | RCTs | Secondary | 1031 | 58±8.5 | 28 | 0.09 | 0.09 | 0.09 | NR |
| Baigent 2011 | RCTs | Mixed | 4650 | 62±12 | 37 | 0.02 | 0.02 | 0.02 | 0.02 |
| Cohen 2012 | Cohort study | Mixed | 10,138 | 61±6 | 39 | 0.12 | NR | NR | NR |
| Esposti 2012 | Cohort study | Mixed | 19,232 | 66±12.8 | 55.2 | 0.23 | NR | NR | NR |
| Pitt 2012 | RCTs | Secondary | 825 | 53±8.8 | 23 | 0.06 | 0.06 | 0.06 | 0.03 |
| Nohara 2012 | RCTs | Secondary | 314 | 64±8.9 | 49 | 0.08 | 0.08 | 0.08 | 0.08 |
| Chen 2013 | RCTs | Primary | 1799 | NR | NR | 0.01 | 0.12 | 0.12 | NR |
| Kim 2013 | RCTs | Primary | 298 | 62±9.2 | 45 | 0.04 | 0.04 | 0.04 | 0.03 |
| Lee 2013 | RCTs | Primary | 132 | 64±7.7 | 45 | 0.07 | 0.07 | NR | 0.05 |
| Liu 2013 | RCTs | Primary | 225 | 59±8.6 | 38 | 0.02 | 0.02 | 0.02 | 0.01 |

*Continued table*

| Sasaki 2013 | RCTs | Primary | 187 | 26±3.5 | 37 | 0.02 | 0.02 | 0.02 | NR |
| --- | --- | --- | --- | --- | --- | --- | --- | --- | --- |
| Zhang 2013 | Cohort study | Mixed | 107,835 | 61±13 | 50 | 0.10 | 0.10 | 0.10 | 0.05 |
| Rosenbaum 2013 | Cohort study | Primary | 1074 | >65 | NR | 0.03 | NR | NR | 0.03 |
| Parker 2013 | RCTs | Primary | 203 | 43.6 ±2.2 | 50 | 0.09 | NR | NR | 0.09 |
| Chang 2013 | Cohort study | Mixed | 18036 | 66±12 | NR | 0.03 | 0.03 | NR | 0.03 |
| Mampuya 2013 | Cohort study | Mixed | 1605 | 64±9.8 | 37 | 0.28 | NR | NR | NR |
| Robison 2014 | Cohort study | Mixed | 10,789 | 67±11 | 52 | 0.25 | 0.25 | NR | 0.04 |
| Ito 2014 | Cohort study | Mixed | 10,138 | 61±6 | 39.4 | 0.15 | NR | NR | 0.15 |
| Quek 2015 | Cohort study | Primary | 136854 | NR | NR | 0.02 | NR | NR | NR |
| Svensson 2015 | Cohort study | Mixed | 161,646 | >65 | 48 | 0.16 | NR | NR | NR |
| Izawa 2015 | RCTs | Secondary | 508 | 66±11 | 19 | 0.03 | 0.03 | 0.03 | NR |
| Vinogradova 2016 | Cohort study | Primary | 570337 | >65 | 47.6 | 0.08 | 0.13 | NR | NR |
| Schulman 2016 | Cohort study | Primary | 990 | 61±12 | 53 | 0.12 | 0.12 | 0.12 | 0.10 |
| Colantonio 2016 | Cohort study | Mixed | 134,863 | 75±9 | 70 | 0.05 | NR | NR | NR |
| Halava 2016 | Cohort study | Mixed | 9285 | >65 | 76 | 0.12 | NR | NR | NR |
| Yusuf 2016 | RCTs | Primary | 6361 | 66±6 | 46 | 0.01 | 0.01 | 0.01 | 0.01 |
| Serban 2017 | Cohort study | Secondary | 105,329 | >65 | NR | 0.02 | NR | NR | NR |
| Brinton 2018 | Cohort study | Primary | 5014 | 64±8 | 48 | 0.05 | 0.05 | 0.05 | 0.05 |
| Ihle 2018 | Cohort study | primary | 531672 | NR | NR | 0.02 | NR | NR | NR |
| Chee 2018 | Cohort study | Mixed | 359 | 85±4 | 54.9 | 0.16 | NR | NR | 0.03 |
| Nagar 2018 | Cohort study | Mixed | 15552 | 56±8 | 36 | 0.09 | NR | NR | NR |
| van Delden 2018 | Cohort study | Primary | 776 | 43±13 | 54 | 0.11 | 0.11 | NR | NR |
| Ofori-Asenso 2018 | Cohort study | Primary | 175,526 | NR | NR | 0.17 | 0.17 | NR | NR |
| Mefford 2018 | Cohort study | Mixed | 7,216 | >65 | 53 | 0.15 | NR | NR | 0.11 |

*Continued table*

| Kajinami 2019 | Cohort study | Secondary | 54,296 | 71±10 | 40.7 | 0.15 | 0.15 | 0.15 | 0.02 |
| --- | --- | --- | --- | --- | --- | --- | --- | --- | --- |
| Chen 2019 | Cohort study | Secondary | 42192 | >65 | NR | 0.20 | NR | NR | NR |
| Ofori-Asenso 2019 | Cohort study | Mixed | 22340 | NR | 51.7 | 0.51 | NR | NR | NR |
| Roh 2019 | Cohort study | Mixed | 8073 | 63±12 | 63 | 0.06 | 0.06 | 0.01 | NR |
| Bradley 2019 | Cohort study | Mixed | 4182 | 68±7 | 40 | 0.11 | NR | NR | NR |
| Jacobson 2019 | Cohort study | Mixed | 1168 | 58.1±13 | 59 | 0.28 | 0.28 | 0.28 | NR |
| Bair 2020 | Cohort study | Secondary | 48997 | 66.3±11.7 | NR | 0.06 | 0.06 | 0.06 | NR |
| Casula 2020 | Cohort study | Primary | 16,717 | 61±12 | 48 | 0.10 | 0.10 | 0.10 | 0.10 |
| Yao 2020 | Cohort study | Secondary | 284954 | NR | NR | 0.05 | NR | NR | NR |
| Thompson 2020 | Cohort study | Mixed | 395,279 | NR | NR | 0.19 | NR | NR | NR |
| Moore 2020 | Cohort study | Secondary | 105,628 | NR | NR | 0.02 | NR | NR | NR |

Abbreviations: NR: non-reported; NLA: National Lipid Association; ILEP: International Lipid Expert Panel; EAS: European Atherosclerosis Society, *±, standard deviation.*

**Table S4.** The list of studies that were excluded from the meta-analysis due to data overlapping.

| 1 | [Dujovne](https://pubmed.ncbi.nlm.nih.gov/?sort=date&term=Dujovne+CA&cauthor_id=1831006) CA, [Chremos](https://pubmed.ncbi.nlm.nih.gov/?sort=date&term=Chremos+AN&cauthor_id=1831006) NA, [Pool](https://pubmed.ncbi.nlm.nih.gov/?sort=date&term=Pool+JL&cauthor_id=1831006) JL, et al.   Expanded clinical evaluation of lovastatin (EXCEL) study results: IV. Additional perspectives on the tolerability of lovastatin. Am J Med. 1991 Jul 31;91(1B):25S-30S. | Overlap |
| --- | --- | --- |
|  |  |  |
| 2 | [Bradford](https://pubmed.ncbi.nlm.nih.gov/?sort=date&term=Bradford+RH&cauthor_id=7942524) RH, [Shear](https://pubmed.ncbi.nlm.nih.gov/?sort=date&term=Shear+CL&cauthor_id=7942524) CL, [Chremos](https://pubmed.ncbi.nlm.nih.gov/?sort=date&term=Chremos+AN&cauthor_id=7942524) AN,  et al. Expanded Clinical Evaluation of Lovastatin (EXCEL) study results: two-year efficacy and safety follow-up. Am J Cardiol. 1994 Oct 1;74(7):667-73. | Overlap |
|  |  |  |
| 3 | [Bradford](https://pubmed.ncbi.nlm.nih.gov/?sort=date&term=Bradford+RH&cauthor_id=8480959) RH, [Downton](https://pubmed.ncbi.nlm.nih.gov/?sort=date&term=Downton+M&cauthor_id=8480959) M, [Chremos](https://pubmed.ncbi.nlm.nih.gov/?sort=date&term=Chremos+AN&cauthor_id=8480959) AN, et al. Efficacy and tolerability of lovastatin in 3390 women with moderate hypercholesterolemia. Ann Intern Med.1993 Jun 1;118(11):850-5. | Overlap |
|  |  |  |
| 4 | [Behounek](https://pubmed.ncbi.nlm.nih.gov/?sort=date&term=Behounek+BD&cauthor_id=8001304) BD, [McGovern](https://pubmed.ncbi.nlm.nih.gov/?sort=date&term=McGovern+ME&cauthor_id=8001304) ME, [Kassler-Taub](https://pubmed.ncbi.nlm.nih.gov/?sort=date&term=Kassler-Taub+KB&cauthor_id=8001304) KB, et al. A multinational study of the effects of low-dose pravastatin in patients with non-insulin-dependent diabetes mellitus and hypercholesterolemia. Pravastatin Multinational Study Group for Diabetes. Clin Cardiol.1994 Oct;17(10):558-62. | Overlap |
|  |  |  |
| 5 | [Cashin-Hemphill](https://pubmed.ncbi.nlm.nih.gov/?sort=date&term=Cashin-Hemphill+L&cauthor_id=1343613) L, [Kramsch](https://pubmed.ncbi.nlm.nih.gov/?sort=date&term=Kramsch+DM&cauthor_id=1343613) DM, [Azen](https://pubmed.ncbi.nlm.nih.gov/?sort=date&term=Azen+SP&cauthor_id=1343613) SP, et al. The Monitored Atherosclerosis Regression Study (MARS). Design, methods and baseline results. ne J Curr Clin Trials.1992 Oct 23;Doc No 26:[9897 words; 83 paragraphs]. | Overlap |
|  |  |  |
| 6 | Mack WJ, et al. [Lipoprotein subclasses in the Monitored Atherosclerosis Regression Study (MARS). Treatment effects and relation to coronary angiographic progression.](https://pubmed.ncbi.nlm.nih.gov/8963728/) Arterioscler Thromb Vasc Biol. 1996. PMID: 8963728 Clinical Trial. | Overlap |
|  |  |  |
| 7 | [Byington](https://pubmed.ncbi.nlm.nih.gov/?sort=date&term=Byington+RP&cauthor_id=10411862) RP, [Evans](https://pubmed.ncbi.nlm.nih.gov/?sort=date&term=Evans+GW&cauthor_id=10411862) GW, [Espeland](https://pubmed.ncbi.nlm.nih.gov/?sort=date&term=Espeland+MA&cauthor_id=10411862) MA, et al. Effects of lovastatin and warfarin on early carotid atherosclerosis: sex-specific analyses. Asymptomatic Carotid Artery Progression Study (ACAPS) Research Group. Circulation.1999 Jul 20;100(3):e14-7. | Overlap |
|  |  |  |
| 8 | [Jacotot](https://pubmed.ncbi.nlm.nih.gov/?sort=date&term=Jacotot+B&cauthor_id=7604799) B, [Benghozi](https://pubmed.ncbi.nlm.nih.gov/?sort=date&term=Benghozi+R&cauthor_id=7604799) R, [Pfister](https://pubmed.ncbi.nlm.nih.gov/?sort=date&term=Pfister+P&cauthor_id=7604799) P, [Holmes](https://pubmed.ncbi.nlm.nih.gov/?sort=date&term=Holmes+D&cauthor_id=7604799) D. Comparison of fluvastatin versus pravastatin treatment of primary hypercholesterolemia. French Fluvastatin Study Group. Am J Cardiol.1995 Jul 13;76(2):54A-56A. | Overlap |
|  |  |  |
| 9 | [Salonen](https://pubmed.ncbi.nlm.nih.gov/?sort=date&term=Salonen+R&cauthor_id=7572684) R, [Nyssönen](https://pubmed.ncbi.nlm.nih.gov/?sort=date&term=Nyss%C3%B6nen+K&cauthor_id=7572684) K, [Porkkala-Sarataho](https://pubmed.ncbi.nlm.nih.gov/?sort=date&term=Porkkala-Sarataho+E&cauthor_id=7572684) E, [Salonen](https://pubmed.ncbi.nlm.nih.gov/?sort=date&term=Salonen+JT&cauthor_id=7572684) JT. The Kuopio Atherosclerosis Prevention Study (KAPS): effect of pravastatin treatment on lipids, oxidation resistance of lipoproteins, and atherosclerotic progression. Am J Cardiol.1995; 76(9):34C-39C. | Overlap |
|  |  |  |
| 10 | [Packard](https://pubmed.ncbi.nlm.nih.gov/?sort=date&term=Packard+CJ&cauthor_id=11036120) CJ, [O'Reilly](https://pubmed.ncbi.nlm.nih.gov/?sort=date&term=O%27Reilly+DS&cauthor_id=11036120) DS, [Caslake](https://pubmed.ncbi.nlm.nih.gov/?sort=date&term=Caslake+MJ&cauthor_id=11036120) MJ, et al. Lipoprotein-associated phospholipase A2 as an independent predictor of coronary heart disease. West of Scotland Coronary Prevention Study Group. Engl J Med.2000; 343(16):1148-55. | Overlap |
|  |  |  |
| 11 | [Furberg](https://pubmed.ncbi.nlm.nih.gov/?sort=date&term=Furberg+CD&cauthor_id=7572689) CD, [Pitt](https://pubmed.ncbi.nlm.nih.gov/?sort=date&term=Pitt+B&cauthor_id=7572689) B, [Byington](https://pubmed.ncbi.nlm.nih.gov/?sort=date&term=Byington+RP&cauthor_id=7572689) RB, [Park](https://pubmed.ncbi.nlm.nih.gov/?sort=date&term=Park+JS&cauthor_id=7572689) JS, [McGovern](https://pubmed.ncbi.nlm.nih.gov/?sort=date&term=McGovern+ME&cauthor_id=7572689) ME. Reduction in coronary events during treatment with pravastatin. PLAC I and PLAC II Investigators. Pravastatin Limitation of Atherosclerosis in the Coronary Arteries. Am J Cardiol.1995 Sep 28;76(9):60C-63C. | Overlap |
|  |  |  |

*Continued table*

| 12 | Jukema JW, Zwinderman AH, van Boven AJ, et al. [Evidence for a synergistic effect of calcium channel blockers with lipid-lowering therapy in retarding progression of coronary atherosclerosis in symptomatic patients with normal to moderately raised cholesterol levels. The REGRESS Study Group.](https://pubmed.ncbi.nlm.nih.gov/8630669/) Arterioscler Thromb Vasc Biol. 1996;16:425-30. | Overlap |
| --- | --- | --- |
|  |  |  |
| 13 | Herd JA. [The lipoprotein and coronary atherosclerosis study (LCAS): lipid and metabolic factors related to atheroma and clinical events.](https://pubmed.ncbi.nlm.nih.gov/9684851/) Am J Med. 1998;104(6A): 42S-49S. | Overlap |
|  |  |  |
| 14 | Pincus J. Comparative dose efficacy study of atorvastatin versus simvastatin, pravastatin, lovastatin, and fluvastatin in patients with hypercholesterolemia (the CURVES study). Am JCardiol.1998;82: 406-7. | Overlap |
|  |  |  |
| 15 | Haq IU, Wallis EJ, Yeo WW, Jackson PR, Ramsay LE. [Coronary events with lipid-lowering therapy: the AFCAPS/TexCAPS trial.](https://pubmed.ncbi.nlm.nih.gov/9952191/) JAMA. 1999; 281:414. | Overlap |
|  |  |  |
| 16 | Downs JR, Clearfield M, Tyroler HA, et al. [Air Force/Texas Coronary Atherosclerosis Prevention Study (AFCAPS/TEXCAPS): additional perspectives on tolerability of long-term treatment with lovastatin.](https://pubmed.ncbi.nlm.nih.gov/11348605/) Am J Cardiol. 2001; 87:1074-9. | Overlap |
|  |  |  |
| 17 | Clearfield M, Downs JR, Weis S, et al. [Air Force/Texas Coronary Atherosclerosis Prevention Study (AFCAPS/TexCAPS): efficacy and tolerability of long-term treatment with lovastatin in women.](https://pubmed.ncbi.nlm.nih.gov/11788107/) J Womens Health Gend Based Med. 2001;10: 971-81 | Overlap |
|  |  |  |
| 18 | Lloyd GW, Jackson G, Foley DP, Boersma E, Shepherd J, Serruys PW. [The influence of plasma lipoprotein (a) on angiographic restenosis and coronary events in patients undergoing planned coronary balloon angioplasty. Ancillary analysis of the Fluvastatin Angioplasty Restenosis (FLARE) trial.](https://pubmed.ncbi.nlm.nih.gov/11583725/) Atherosclerosis. 200;158(2):445-54. | Overlap |
|  |  |  |
| 19 | Kinlay S, Schwartz GG, Olsson AG, et al. [Effect of atorvastatin on risk of recurrent cardiovascular events after an acute coronary syndrome associated with high soluble CD40 ligand in the Myocardial Ischemia Reduction with Aggressive Cholesterol Lowering (MIRACL) Study.](https://pubmed.ncbi.nlm.nih.gov/15262833/) Circulation. 2004;110: 386-91. | Overlap |
|  |  |  |
| 20 | van Wissen S, Smilde TJ, de Groot E, et al. [The significance of femoral intima-media thickness and plaque scoring in the Atorvastatin versus Simvastatin on Atherosclerosis Progression (ASAP) study.](https://pubmed.ncbi.nlm.nih.gov/14671468/) Eur J Cardiovasc Prev Rehabil. 2003;10: 451-5. | Overlap |
|  |  |  |
| 21 | van Venrooij FV, van de Ree MA, et al. [Aggressive lipid lowering does not improve endothelial function in type 2 diabetes: the Diabetes Atorvastatin Lipid Intervention (DALI) Study: a randomized, double-blind, placebo-controlled trial.](https://pubmed.ncbi.nlm.nih.gov/12087021/) Diabetes Care. 2002 Jul;25(7):1211-6. | Overlap |
|  |  |  |
| 22 | van de Ree MA, Huisman MV, Princen HM, et al. [Strong decrease of high sensitivity C-reactive protein with high-dose atorvastatin in patients with type 2 diabetes mellitus.](https://pubmed.ncbi.nlm.nih.gov/12482559/) Atherosclerosis. 2003;166(1):129-35. | Overlap |
|  |  |  |

*Continued table*

| 23 | Berk-Planken II, Hoogerbrugge N, Stolk RP, et al. [Atorvastatin dose-dependently decreases hepatic lipase activity in type 2 diabetes: effect of sex and the LIPC promoter variant.](https://pubmed.ncbi.nlm.nih.gov/12547874/) Diabetes Care. 2003; 26:427-32. | Overlap |
| --- | --- | --- |
|  |  |  |
| 24 | van Hoek M, van Tol A, van Vark-van der Zee LC, et al. [Role of plasma adiponectin on the HDL-cholesterol raising effect of atorvastatin in patients with type 2 diabetes.](https://pubmed.ncbi.nlm.nih.gov/19210142/) Curr Med Res Opin. 2009 Jan;25(1):93-101. | Overlap |
|  |  |  |
| 25 | Collins R, Armitage J, Parish S, Sleigh P, Peto R; Heart Protection Study Collaborative Group. [MRC/BHF Heart Protection Study of cholesterol-lowering with simvastatin in 5963 people with diabetes: a randomised placebo-controlled trial.](https://pubmed.ncbi.nlm.nih.gov/12814710/) Lancet. 2003 Jun 14;361(9374):2005-16. | Overlap |
|  |  |  |
| 26 | Collins R, Armitage J. [High-risk elderly patients PROSPER from cholesterol-lowering therapy.](https://pubmed.ncbi.nlm.nih.gov/12457780/) Lancet. 2002 Nov 23;360(9346):1618-9. | Overlap |
|  |  |  |
| 27 | Arampatzis CA, Goedhart D, Serruys PW, et al; LIPS Investigators. [Fluvastatin reduces the impact of diabetes on long-term outcome after coronary intervention--a Lescol Intervention Prevention Study (LIPS) substudy.](https://pubmed.ncbi.nlm.nih.gov/15846273/) Am Heart J. 2005 Feb;149(2):329-35. | Overlap |
|  |  |  |
| 28 | [Foody](https://pubmed.ncbi.nlm.nih.gov/?sort=date&term=Foody+JM&cauthor_id=18774618) MJ, [Joyce](https://pubmed.ncbi.nlm.nih.gov/?sort=date&term=Joyce+AT&cauthor_id=18774618) AT, [Jeffers](https://pubmed.ncbi.nlm.nih.gov/?sort=date&term=Jeffers+BW&cauthor_id=18774618) BW et al. A large observational study of cardiovascular outcomes associated with atorvastatin or simvastatin therapy in diabetic patients without prior cardiovascular disease. Diabetes Res Clin Pract.2008 Oct;82(1):e13-5. | Overlap |
|  |  |  |
| 29 | Jardine AG, Holdaas H, Fellström B, et al. [fluvastatin prevents cardiac death and myocardial infarction in renal transplant recipients: post-hoc subgroup analyses of the ALERT Study.](https://pubmed.ncbi.nlm.nih.gov/15147434/) Am J Transplant. 2004 Jun;4(6):988-95. | Overlap |
|  |  |  |
| 30 | Stender S, Schuster H, Barter P, Watkins C, Kallend D; MERCURY I Study Group. [Comparison of rosuvastatin with atorvastatin, simvastatin and pravastatin in achieving cholesterol goals and improving plasma lipids in hypercholesterolaemic patients with or without the metabolic syndrome in the MERCURY I trial.](https://pubmed.ncbi.nlm.nih.gov/15955130/) Diabetes Obes Metab. 2005 Jul;7(4):430-8. | Overlap |
|  |  |  |
| 31 | Beishuizen ED, Tamsma JT, Jukema JW, van de Ree MA, van der Vijver JC, Meinders AE, Huisman MV. [The effect of statin therapy on endothelial function in type 2 diabetes without manifest cardiovascular disease.](https://pubmed.ncbi.nlm.nih.gov/15983318/) Diabetes Care. 2005 Jul;28(7):1668-74. | Overlap |
|  |  |  |
| 32 | Bays HE, Ose L, Fraser N, et al. [A multicenter, randomized, double-blind, placebo-controlled, factorial design study to evaluate the lipid-altering efficacy and safety profile of the ezetimibe/simvastatin tablet compared with ezetimibe and simvastatin monotherapy in patients with primary hypercholesterolemia.](https://pubmed.ncbi.nlm.nih.gov/15639688/) Clin Ther. 2004 Nov;26(11):1758-73. | Overlap |
|  |  |  |
| 33 | Nissen SE. [Effect of intensive lipid lowering on progression of coronary atherosclerosis: evidence for an early benefit from the Reversal of Atherosclerosis with Aggressive Lipid Lowering (REVERSAL) trial.](https://pubmed.ncbi.nlm.nih.gov/16126025/) Am J Cardiol. 2005;96(5A):61F-68F. | Overlap |
|  |  |  |

*Continued table*

| 34 | Murad O, Palmer J, Sowers J, et al. [Statins and CVD prevention in the diabetic population: implications of the CARDS trial.](https://pubmed.ncbi.nlm.nih.gov/15929865/) Curr Diab Rep. 2005; 5:191-3. | Overlap |
| --- | --- | --- |
|  |  |  |
| 35 | Kjekshus J, Pedersen TR. [Reducing the risk of coronary events: evidence from the Scandinavian Simvastatin Survival Study (4S).](https://pubmed.ncbi.nlm.nih.gov/7572690/)Kjekshus J, Pedersen TR. Am J Cardiol. 1995;76(9):64C-68C. | Overlap |
|  |  |  |
| 36 | Miettinen TA, Pyörälä K, Olsson AG, et al. [Cholesterol-lowering therapy in women and elderly patients with myocardial infarction or angina pectoris: findings from the 4S.](https://pubmed.ncbi.nlm.nih.gov/9416884/) Circulation. 1997; 96(12):4211-8. | Overlap |
|  |  |  |
| 37 | Mizuno K, Nakaya N, Ohashi Y, et al. [Usefulness of pravastatin in primary prevention of cardiovascular events in women: analysis of the Management of Elevated Cholesterol in the Primary Prevention Group of Adult Japanese (MEGA study).](https://pubmed.ncbi.nlm.nih.gov/18172039/) Circulation. 2008 Jan 29;117(4):494-502. | Overlap |
|  |  |  |
| 38 | Chhatriwalla AK, Nicholls SJ, Nissen SE. [The ASTEROID trial: coronary plaque regression with high-dose statin therapy.](https://pubmed.ncbi.nlm.nih.gov/19804256/) Future Cardiol. 2006;2:651-4. | Overlap |
|  |  |  |
| 39 | Foody JM, Brown WV, Zieve F, et al. [Safety and efficacy of ezetimibe/simvastatin combination versus atorvastatin alone in adults ≥65 years of age with hypercholesterolemia and with or at moderately high/high risk for coronary heart disease (the VYTELD study).](https://pubmed.ncbi.nlm.nih.gov/21029821/) Am J Cardiol. 2010;106:1255-63. | Overlap |
|  |  |  |
| 40 | Zhu JR, Tomlinson B, Ro YM, Sim KH, Lee YT, Sriratanasathavorn C. [A randomised study comparing the efficacy and safety of rosuvastatin with atorvastatin for achieving lipid goals in clinical practice in Asian patients at high risk of cardiovascular disease (DISCOVERY-Asia study).](https://pubmed.ncbi.nlm.nih.gov/18196620/) Curr Med Res Opin. 2007;23:3055-68. | Overlap |
|  |  |  |
| 41 | Haddad RM, Ballantyne CM. [METEOR Trial Reports on the Effect of Rosuvastatin on Progression of Carotid Intima-Media Thickness in Low-Risk Individuals with Subclinical Atherosclerosis.](https://pubmed.ncbi.nlm.nih.gov/20631478/) Phys Sportsmed. 2010; 38:180-2. | Overlap |
|  |  |  |
| 42 | Bots ML, Palmer MK, Dogan S, et al. [Intensive lipid lowering may reduce progression of carotid atherosclerosis within 12 months of treatment: the METEOR study.](https://pubmed.ncbi.nlm.nih.gov/19298496/) J Intern Med. 2009; 265: 698-707. | Overlap |
|  |  |  |
| 43 | Crouse JR 3rd, Bots ML, Evans GW, et al. [Does baseline carotid intima-media thickness modify the effect of rosuvastatin when compared with placebo on carotid intima-media thickness progression? The METEOR study.](https://pubmed.ncbi.nlm.nih.gov/20038840/) Eur J Cardiovasc Prev Rehabil. 2010;17: 223-9. | Overlap |
|  |  |  |
| 44 | Clearfield M. [Effects of lipid-lowering therapy on coronary heart disease in older patients: the SAGE study.](https://pubmed.ncbi.nlm.nih.gov/18383620/) Curr Atheroscler Rep. 2008;10(1):5-6. | Overlap |
| 45 | [Tavazzi L, Maggioni AP, Marchioli R, et al. Effect of n-3 polyunsaturated fatty acids in patients with chronic heart failure (the GISSI-HF trial): a randomised, double-blind, placebo-controlled trial.](https://pubmed.ncbi.nlm.nih.gov/18757090/) Lancet. 2008; 372:1223-30. |  |

*Continued table*

| 46 | Charlton-Menys V, Betteridge DJ, et al [Targets of statin therapy: LDL cholesterol, non-HDL cholesterol, and apolipoprotein B in type 2 diabetes in the Collaborative Atorvastatin Diabetes Study (CARDS).](https://pubmed.ncbi.nlm.nih.gov/19147732/) Clin Chem. 2009 Mar;55(3):473-80. | Overlap |
| --- | --- | --- |
|  |  |  |
| 47 | Yokote K, Saito Y; CHIBA. [Influence of statins on glucose tolerance in patients with type 2 diabetes mellitus: subanalysis of the collaborative study on hypercholesterolemia drugintervention and their benefits for atherosclerosis prevention (CHIBA study).](https://pubmed.ncbi.nlm.nih.gov/19556714/) J Atheroscler Thromb. 2009 Jun;16(3):297-8. | Overlap |
|  |  |  |
| 48 | Barth JH, Jackson BM, Farrin AJ, et al. [Change in serum lipids after acute coronary syndromes: secondary analysis of SPACE ROCKET study data and a comparative literature review.](https://pubmed.ncbi.nlm.nih.gov/20729301/) Clin Chem. 2010 Oct;56(10):1592-8. | Overlap |
|  |  |  |
| 49 | Hsia J, MacFadyen JG, Monyak J, Ridker PM. [Cardiovascular event reduction and adverse events among subjects attaining low-density lipoprotein cholesterol <50 mg/dl with rosuvastatin. The JUPITER trial (Justification for the Use of Statins in Prevention: an Intervention Trial Evaluating Rosuvastatin).](https://pubmed.ncbi.nlm.nih.gov/21492764/) J Am Coll Cardiol. 2011;57(16):1666-75. | Overlap |
|  |  |  |
| 50 | Upadhyay A, Weiner DE. [Lipid-lowering therapy in individuals with CKD: lessons learned from SHARP.](https://pubmed.ncbi.nlm.nih.gov/22033284/) Am J Kidney Dis. 2012; 59(:170-3. | Overlap |
|  |  |  |
| 51 | Berthold HK, Krone W, Erdmann E, Gouni-Berthold I. [Lipid lowering in patients with chronic kidney disease: a SHARP turn in the wrong direction?](https://pubmed.ncbi.nlm.nih.gov/22087046/) Eur J Cardiovasc Prev Rehabil. 2011 Dec;18(6):858-61. | Overlap |
|  |  |  |
| 52 | Wei MY, Ito MK, Cohen JD, Brinton EA, Jacobson TA. [Predictors of statin adherence, switching, and discontinuation in the USAGE survey: understanding the use of statins in America and gaps in patient education.](https://pubmed.ncbi.nlm.nih.gov/24079289/) J Clin Lipidol. 2013t;7(5):472-83. | Overlap |
|  |  |  |
| 53 | Yokoi H, Nohara R, Daida H, Hata M, et al. [Change in carotid intima-media thickness in a high-risk group of patients by intensive lipid-lowering therapy with rosuvastatin: subanalysis of the JART study.](https://pubmed.ncbi.nlm.nih.gov/24632963/) Int Heart J. 2014;55(2):146-52. | Overlap |
|  |  |  |
| 54 | Daida H, Nohara R, Hata M, Kaku K, et al. [Can intensive lipid-lowering therapy improve the carotid intima-media thickness in Japanese subjects under primary prevention for cardiovascular disease?: The JART and JART extension subanalysis.](https://pubmed.ncbi.nlm.nih.gov/24953046/) J Atheroscler Thromb. 2014;21(7):739-54 | Overlap |
|  |  |  |
| 55 | Miura T, Izawa A, Motoki H, et al. [Clinical Impact of Rapid Reduction of Low-Density Lipoprotein Cholesterol Level on Long-Term Outcome of Acute Myocardial Infarction in the Statin Era: Subanalysis of the ALPS-AMI Study.](https://pubmed.ncbi.nlm.nih.gov/26083546/) PLoS One. 2015 Jun 17;10(6):e0127835. | Overlap |
|  |  |  |
| 56 | Colantonio LD, Huang L, Monda KL, et al. [Adherence to High-Intensity Statins Following a Myocardial Infarction Hospitalization Among Medicare Beneficiaries.](https://pubmed.ncbi.nlm.nih.gov/28423147/) JAMA Cardiol. 2017 Aug 1;2(8):890-895. | Overlap |
|  |  |  |

**Figure S1**. Definition of statin intolerance

**
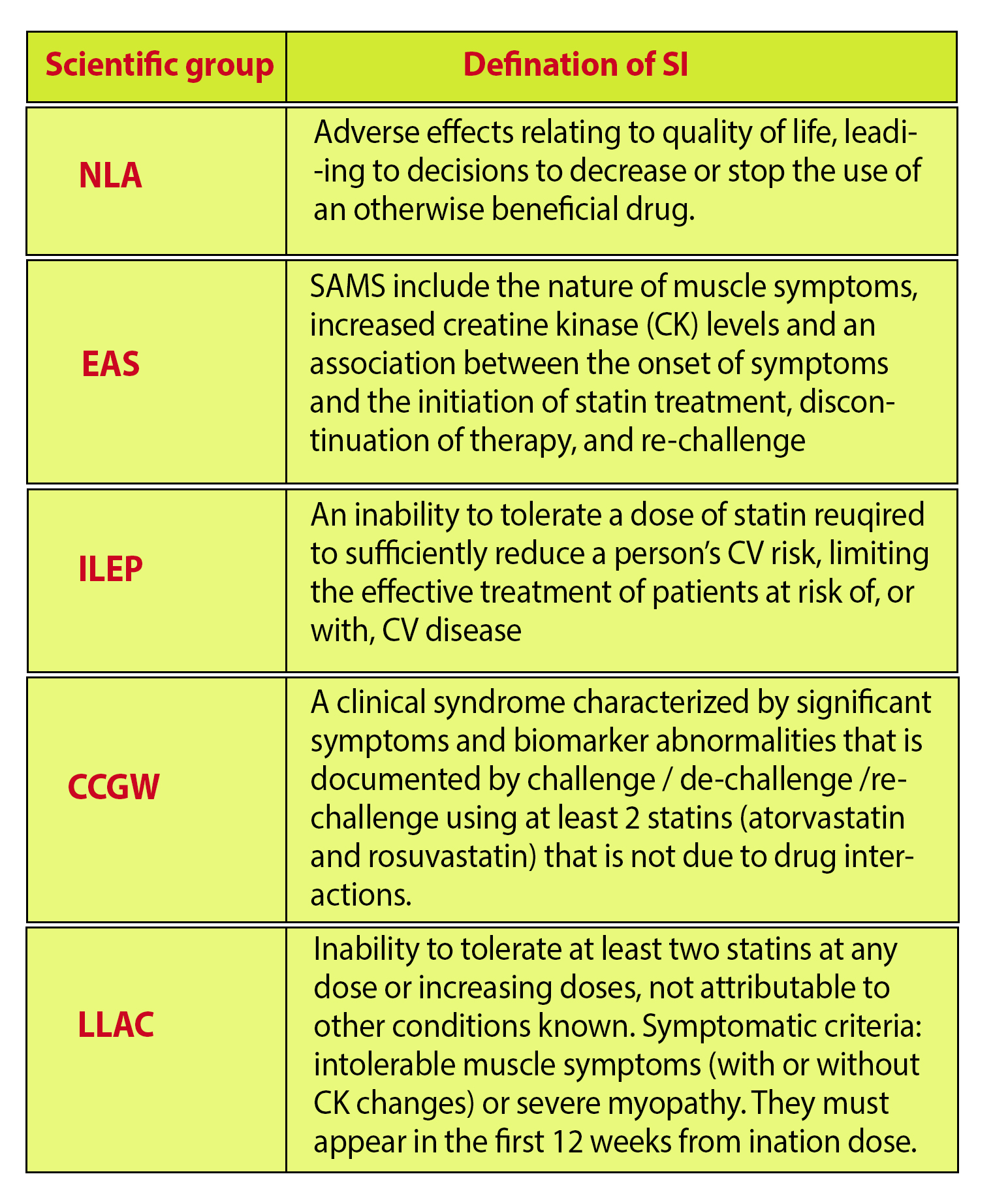
**

*Abbreviations: NLA: National Lipid Association; EAS: European Atherosclerosis Society; ILEP: International Lipid Expert Panel; CCGW: Canadian Consensus Working Group; LLAC: Luso-Latin American Consortium*

**Figure S2.** Pooled prevalence of statin intolerance

**
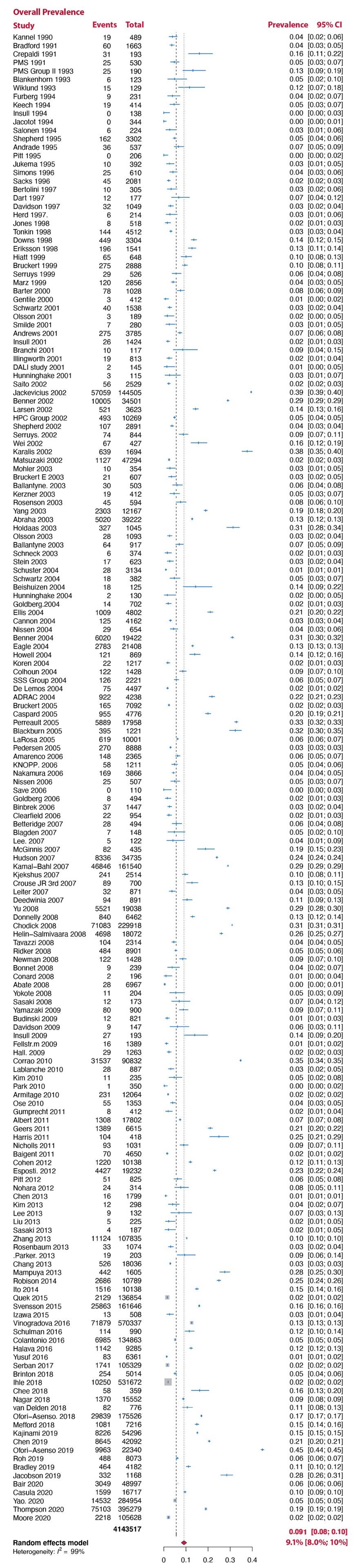
**

*Note:* D-L random-effects model was used

**Figure S3.** Prevalence of statin intolerance based on NLA criteria


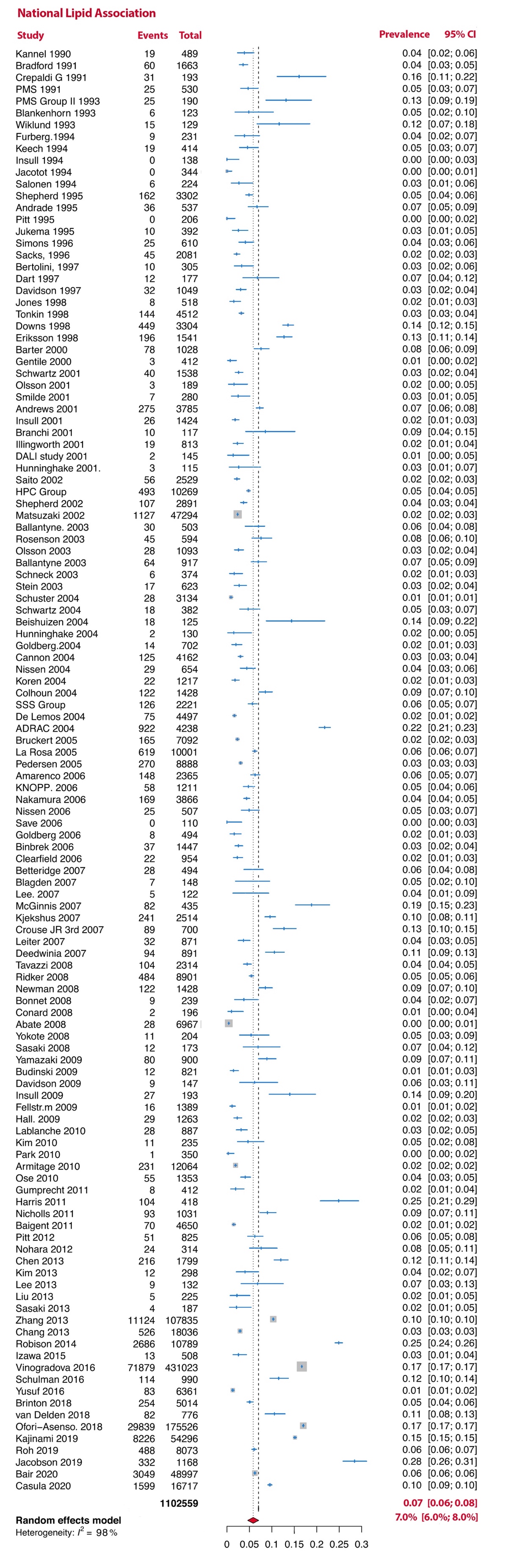


*Abbreviations: NLA: National Lipid Association.*

*Note:* D-L random-effects model used

**Figure S4.** Prevalence of statin intolerance based on EAS criteria


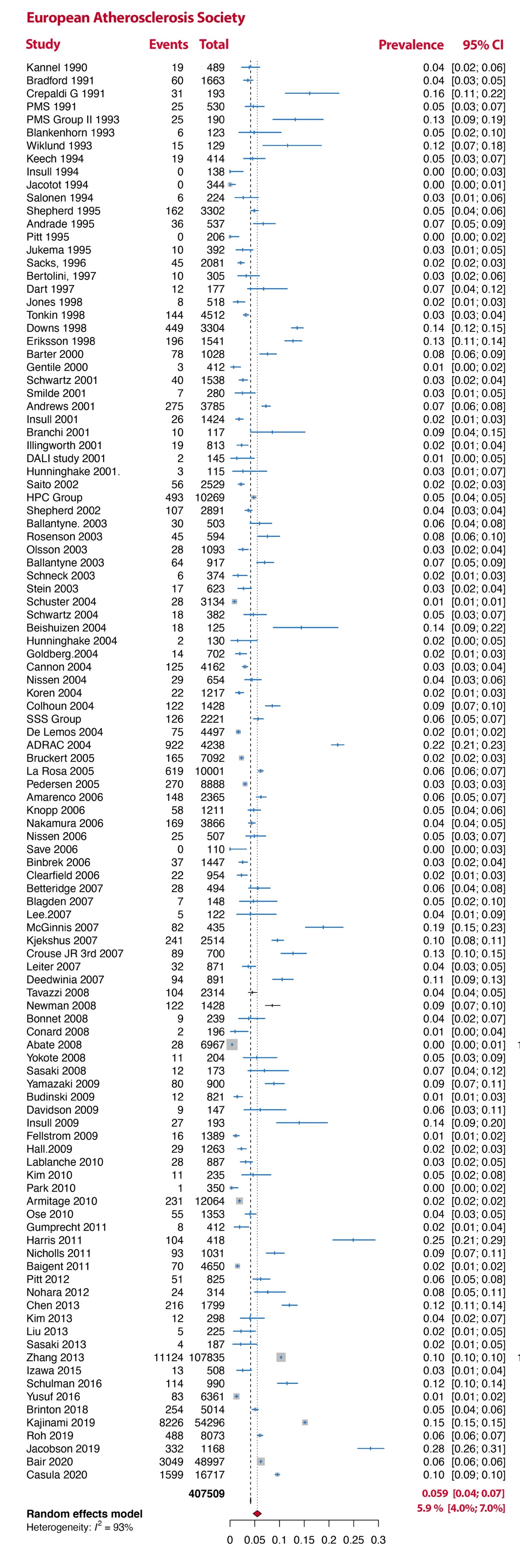


*Abbreviations; EAS: European Association Society.*

*Note:* D-L random-effects model used

**Figure S5.** Prevalence of statin intolerance based on ILEP criteria


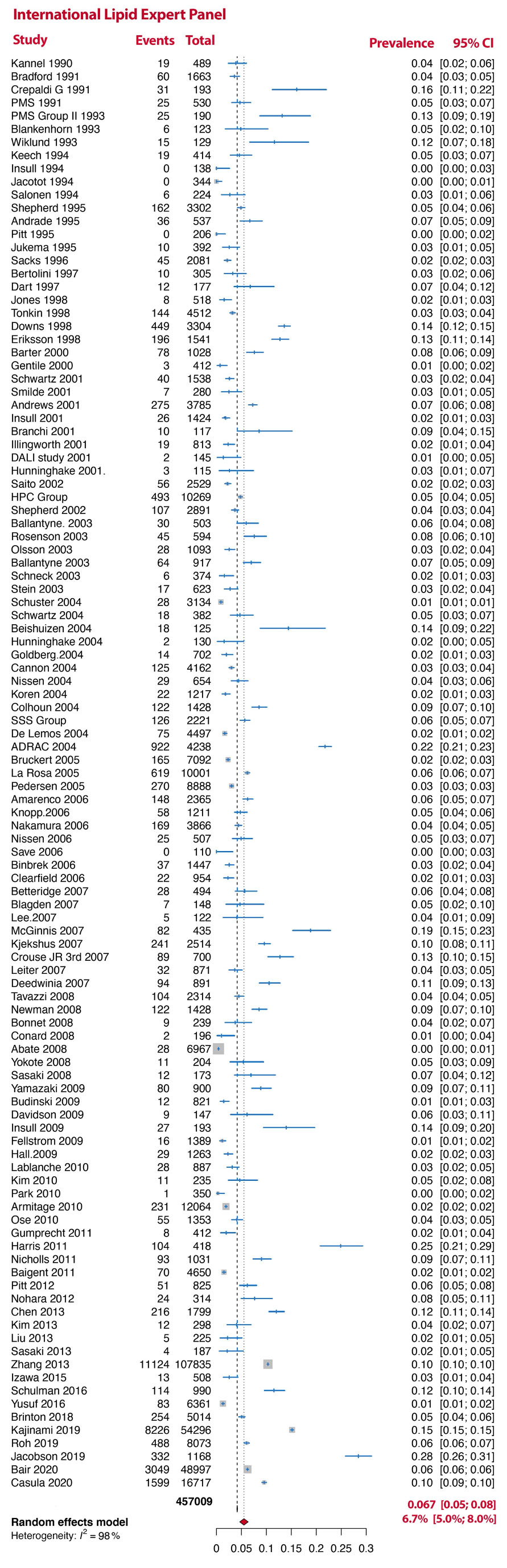


*Abbreviations: ILEP: International Lipid Expert Panel.*

*Note:* D-L random-effects model used

**Figure S6.** Prevalence of statin intolerance in RCT studies


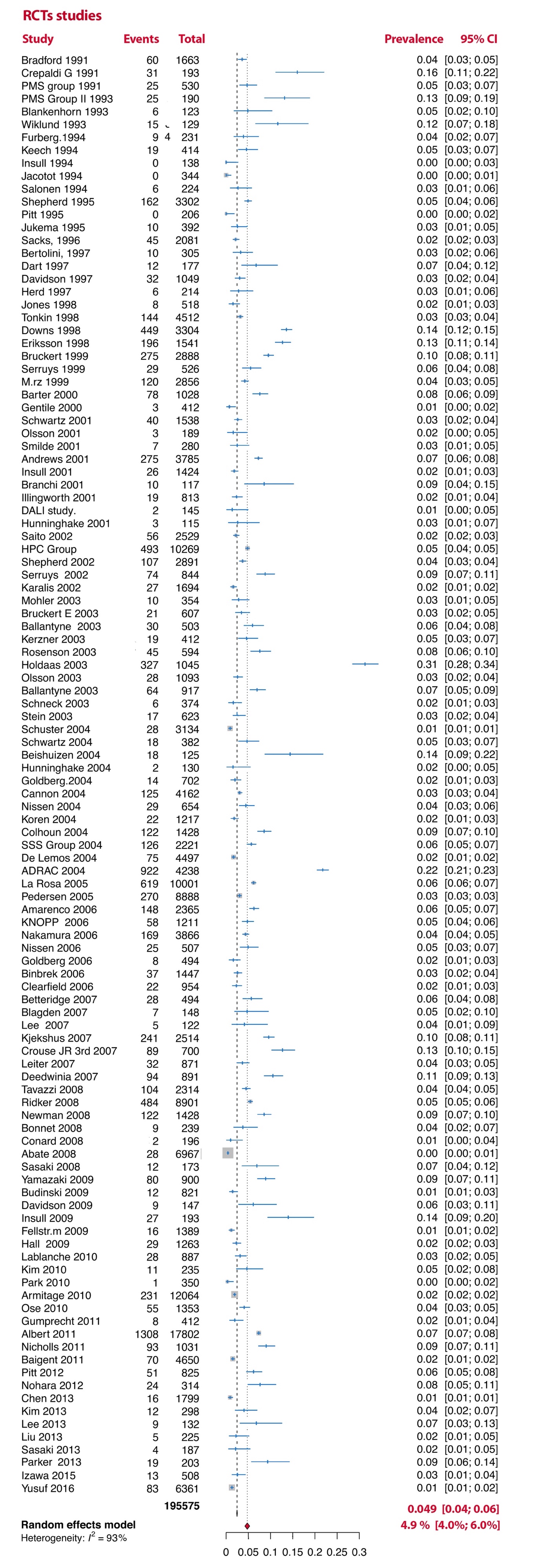


*Abbreviations: RCTs: Randomized Clinical Trials.*

*Note:* D-L random-effects model used

**Figure S7.** Prevalence of statin intolerance in cohort studies


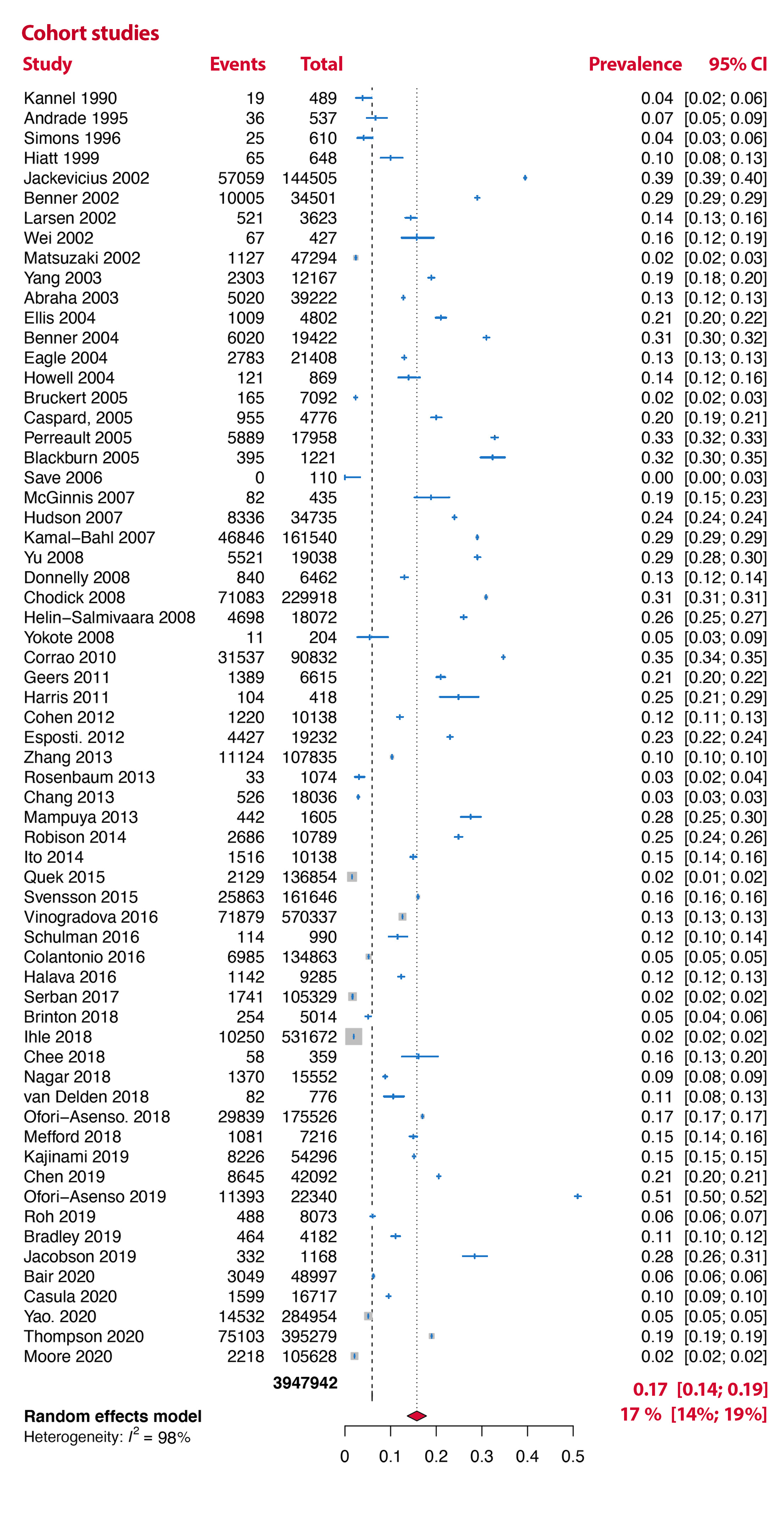


*Note:* D-L random-effects model used

**Figure S8.** Prevalence of statin intolerance on different disease in the primary prevention


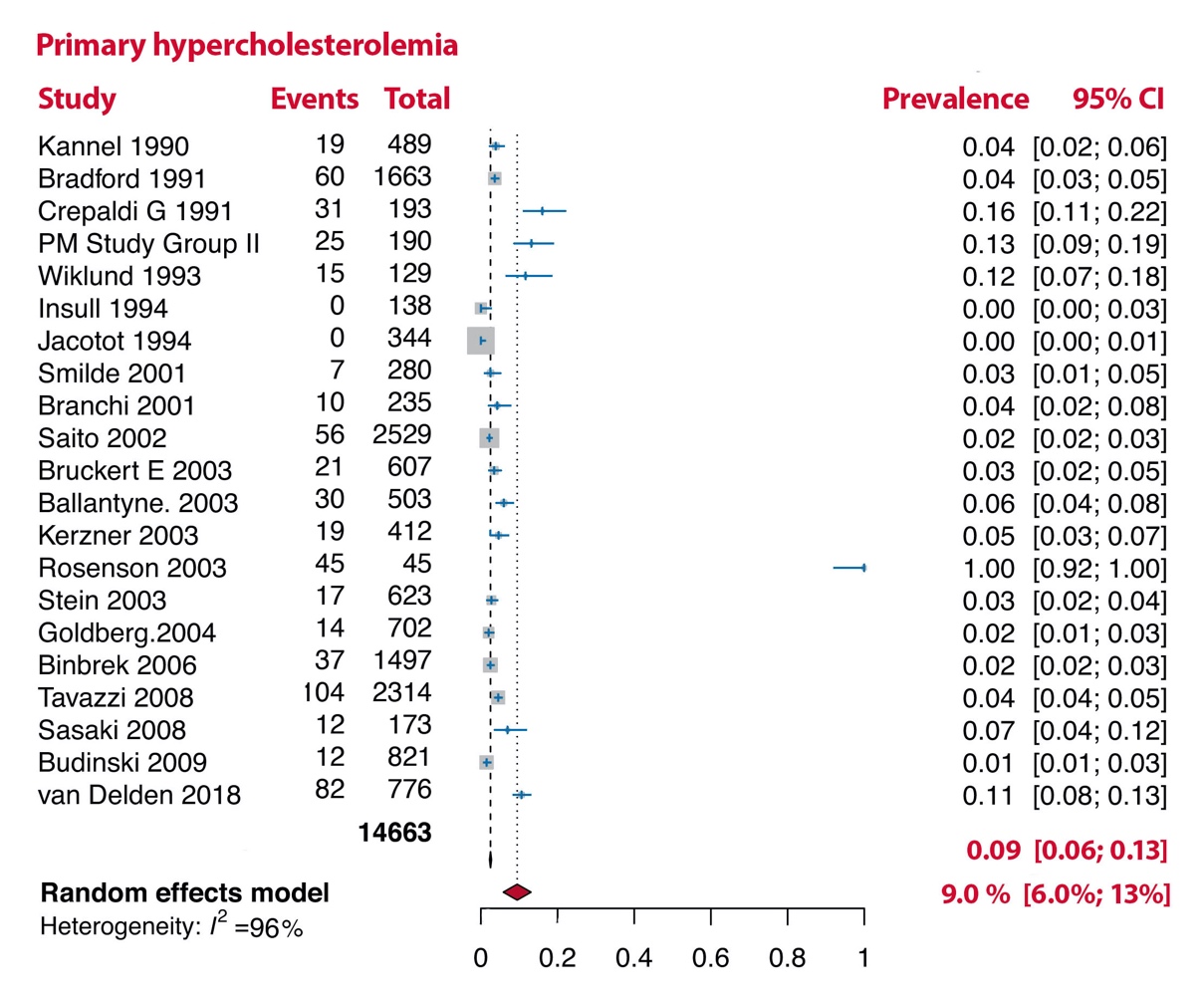


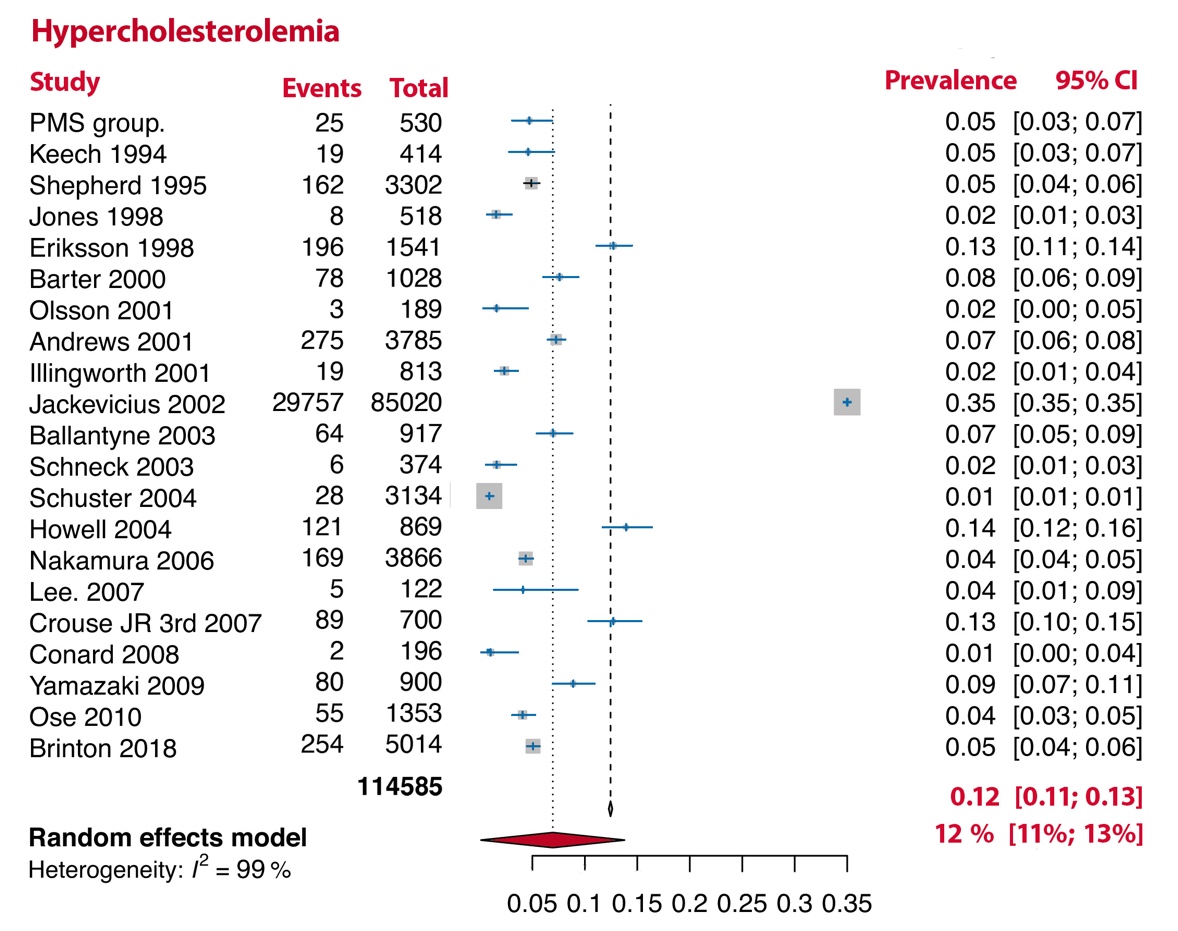


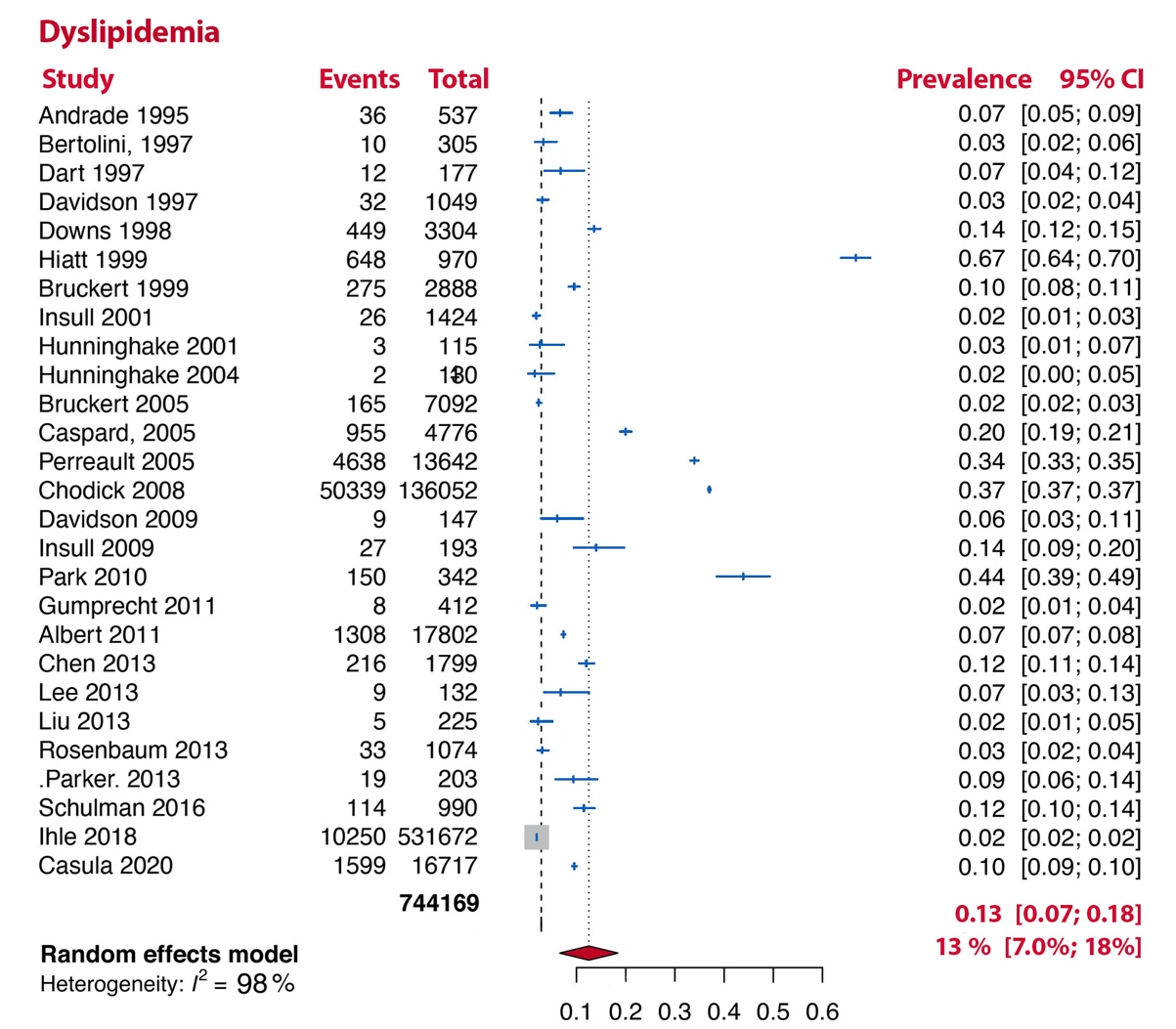


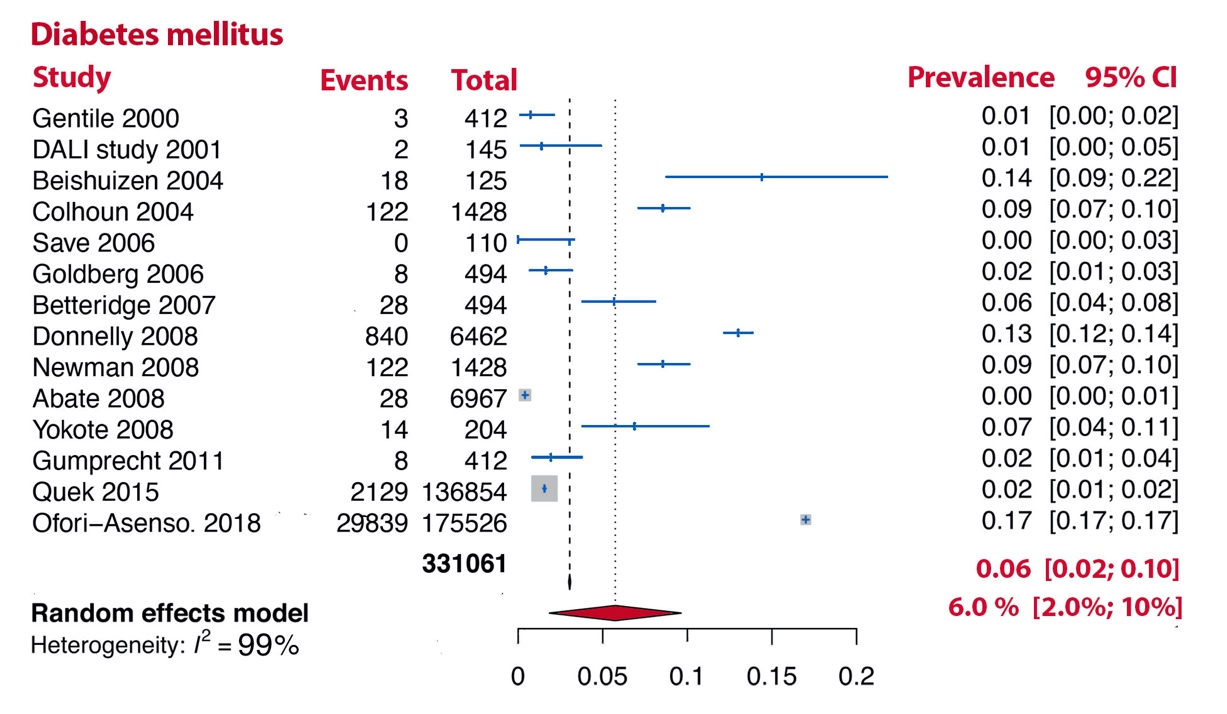


*Note:* D-L random-effects model used

**Figure S9.** Prevalence of statin intolerance on different disease in the secondary prevention


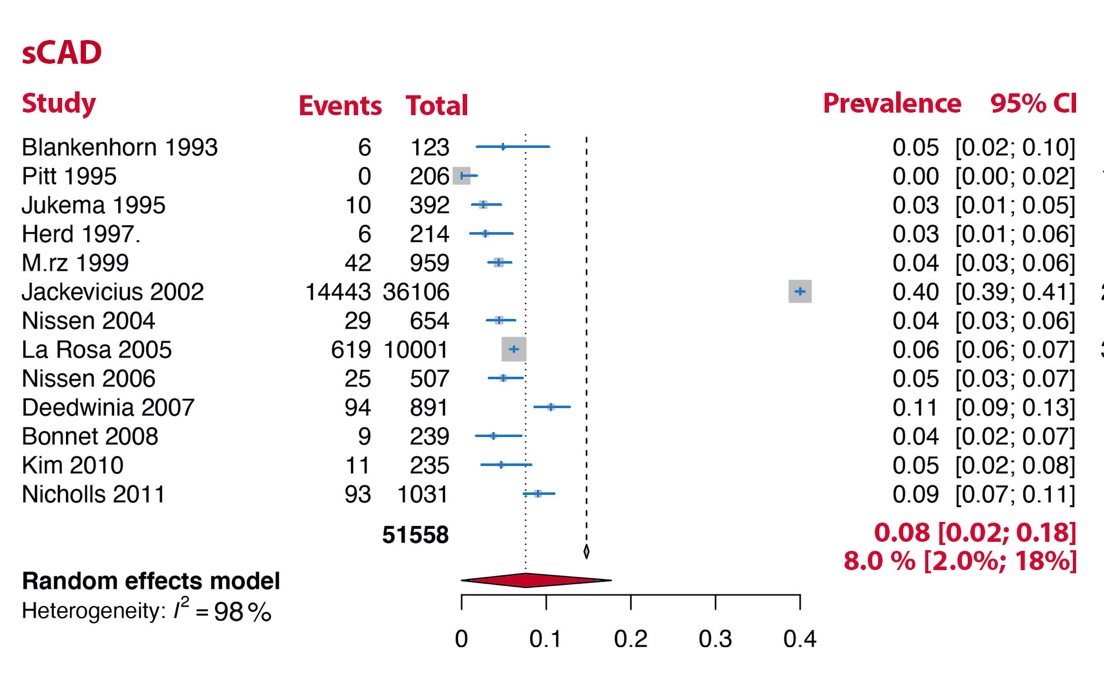


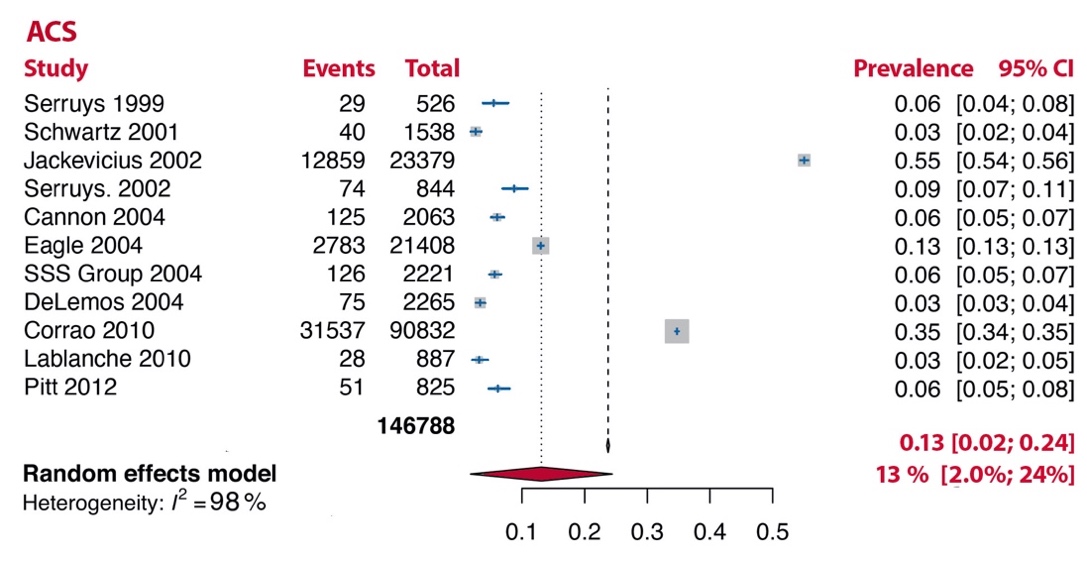


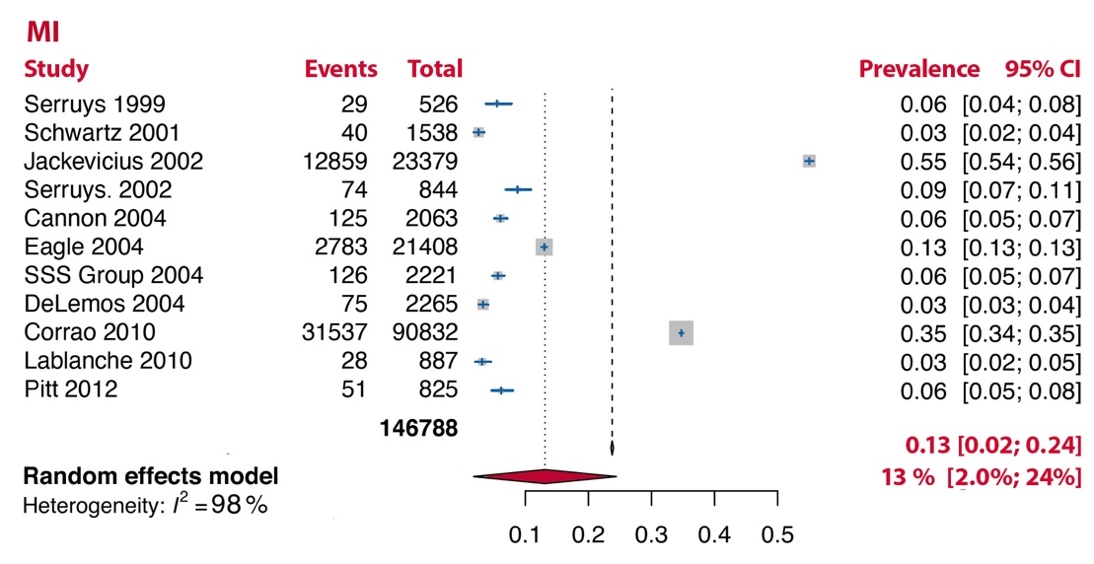


*Abbreviations: sCAD: Stable coronary artery disease; ACS: Acute coronary syndrome; MI: Myocardial infarction. Note:* D-L random-effects model used

**Figure S10.** Prevalence of statin intolerance on different disease from hydrophilic

statins


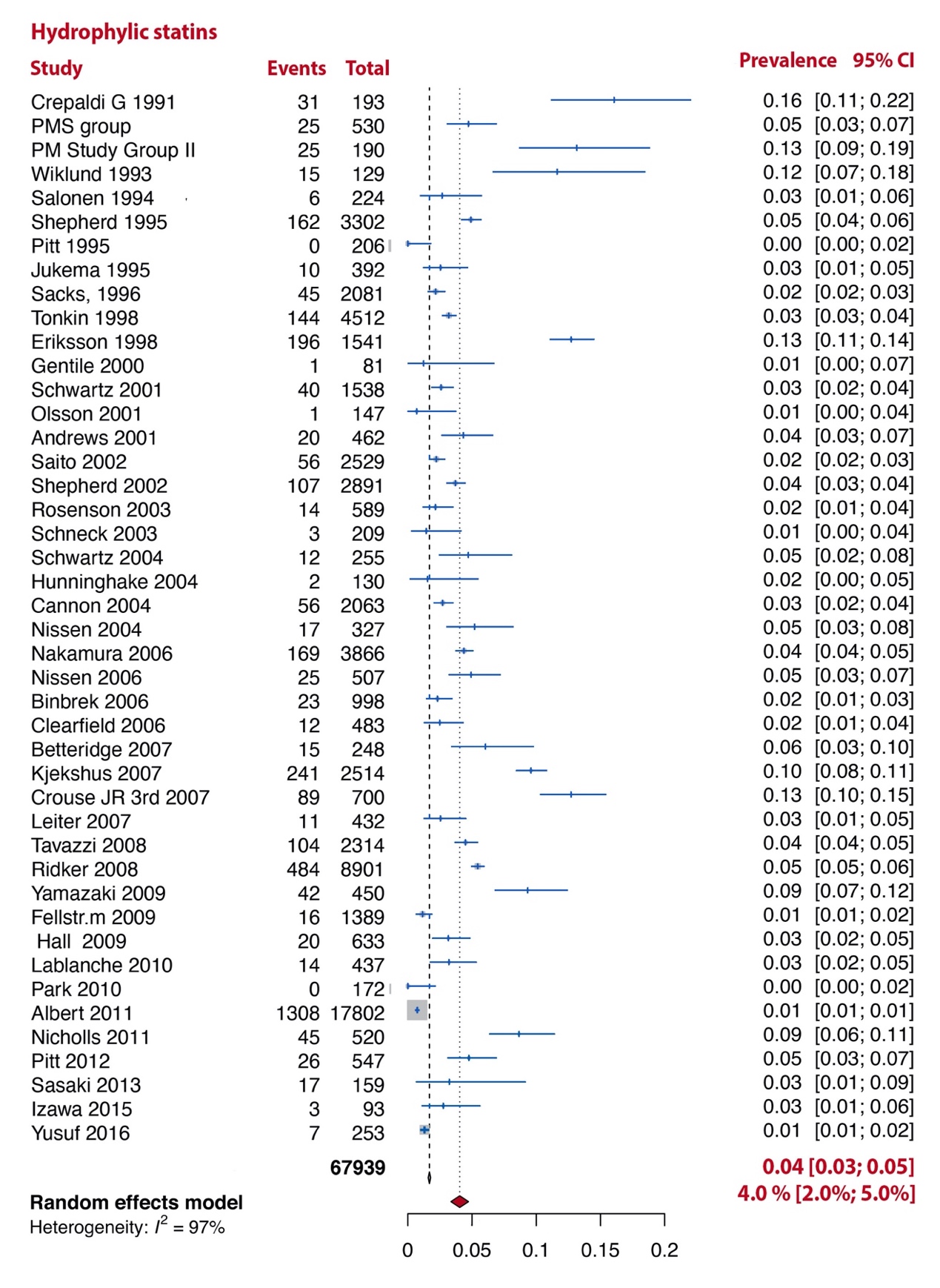


*Note:* D-L random-effects model used

**Figure S11.** Prevalence of statin intolerance on different disease from lipophilic statins

**
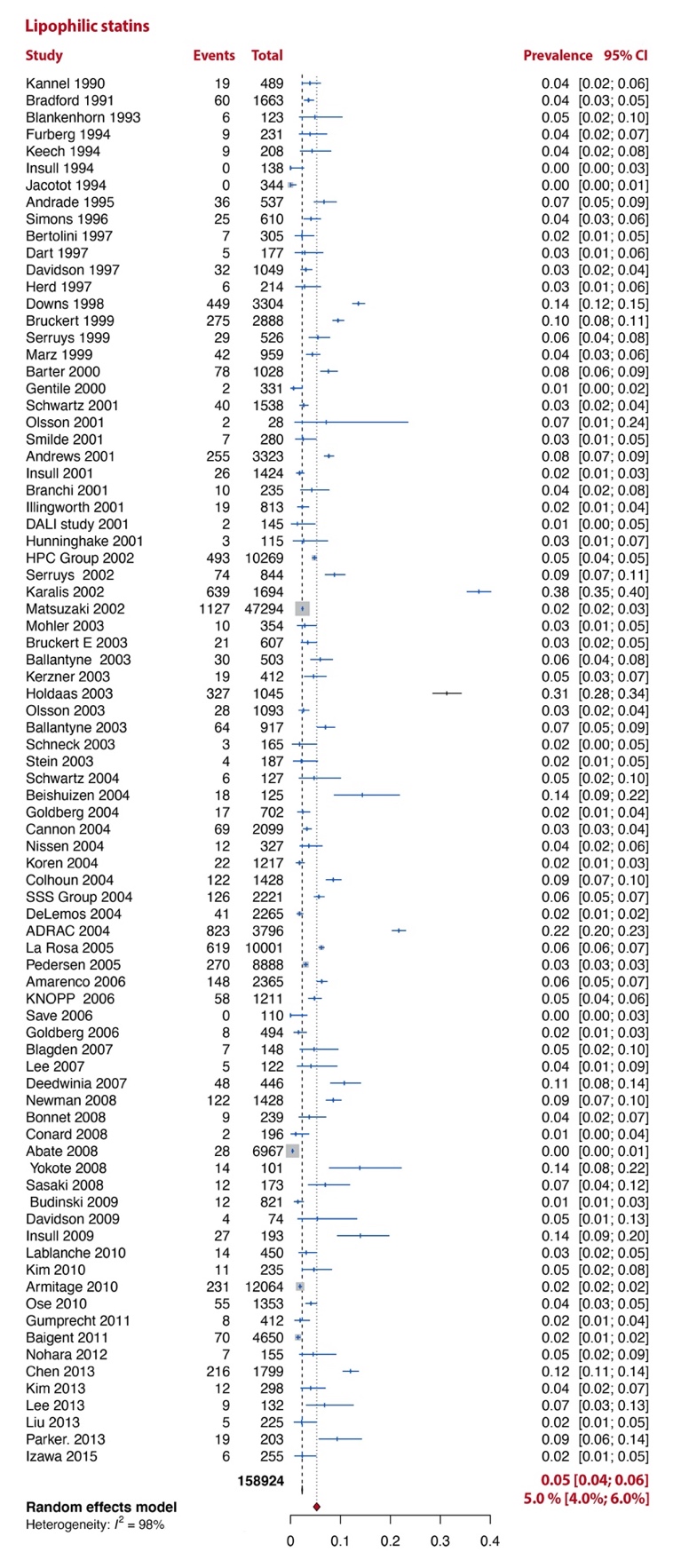
**

*Note:* D-L random-effects model used

**Figure S 12.** Meta-regression of demographic indices on SI: A) Age; B) Age ≥ 65 years; C) Female gender; D) white race; E) caucasian race; F) Hispanic race; G) Asian race; H) Black race.


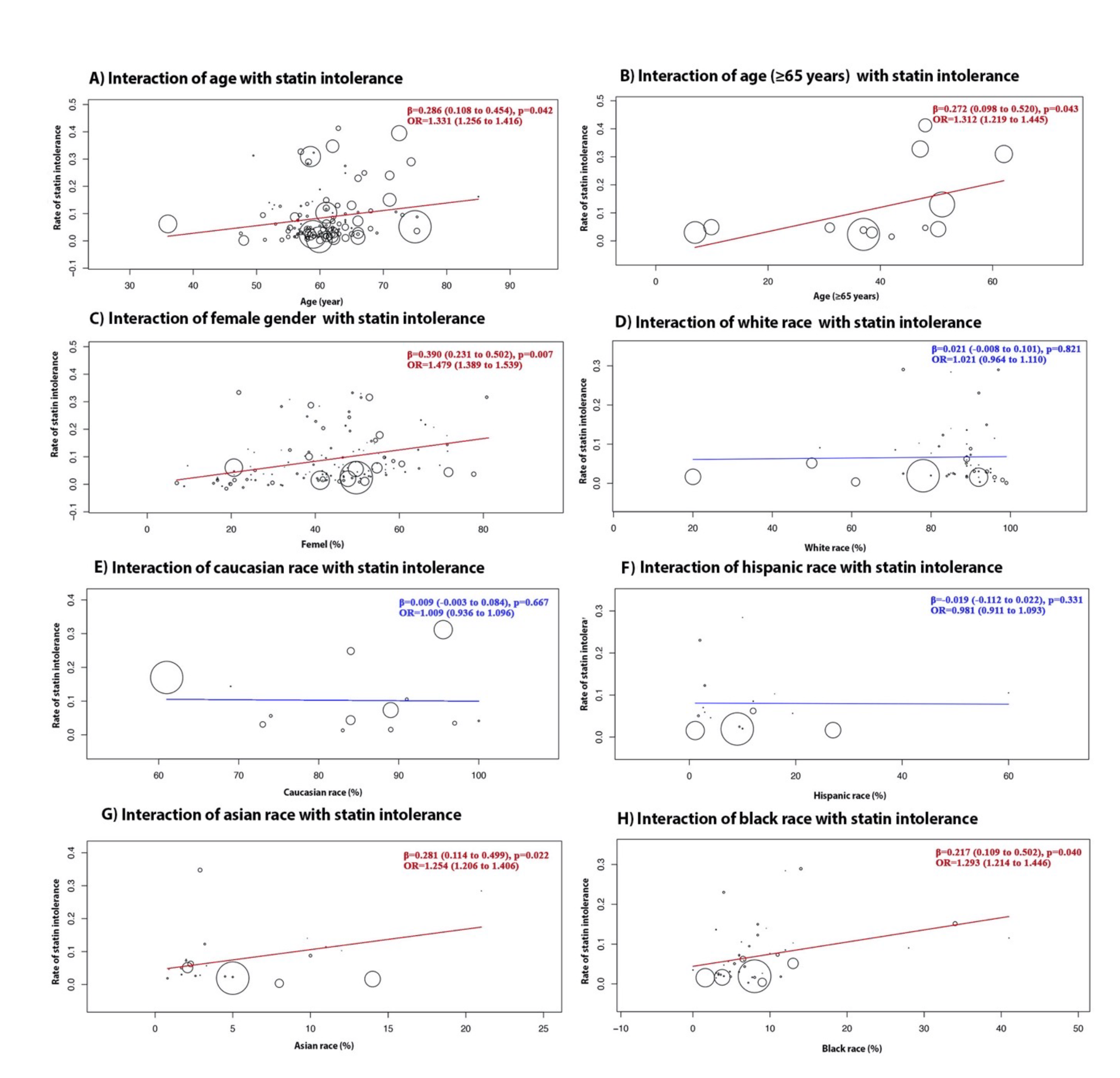


*Note:* D-L random-effects model used

**Figure S 13.** Meta-regression of risk factors on SI:

1. Arterial hypertension; B) Diabetes mellitus; C) Chronic renal failure; D) Obesity; E) Depression; F) Hypothyroidism; G) Chronic liver disease; H) Chronic renal failure


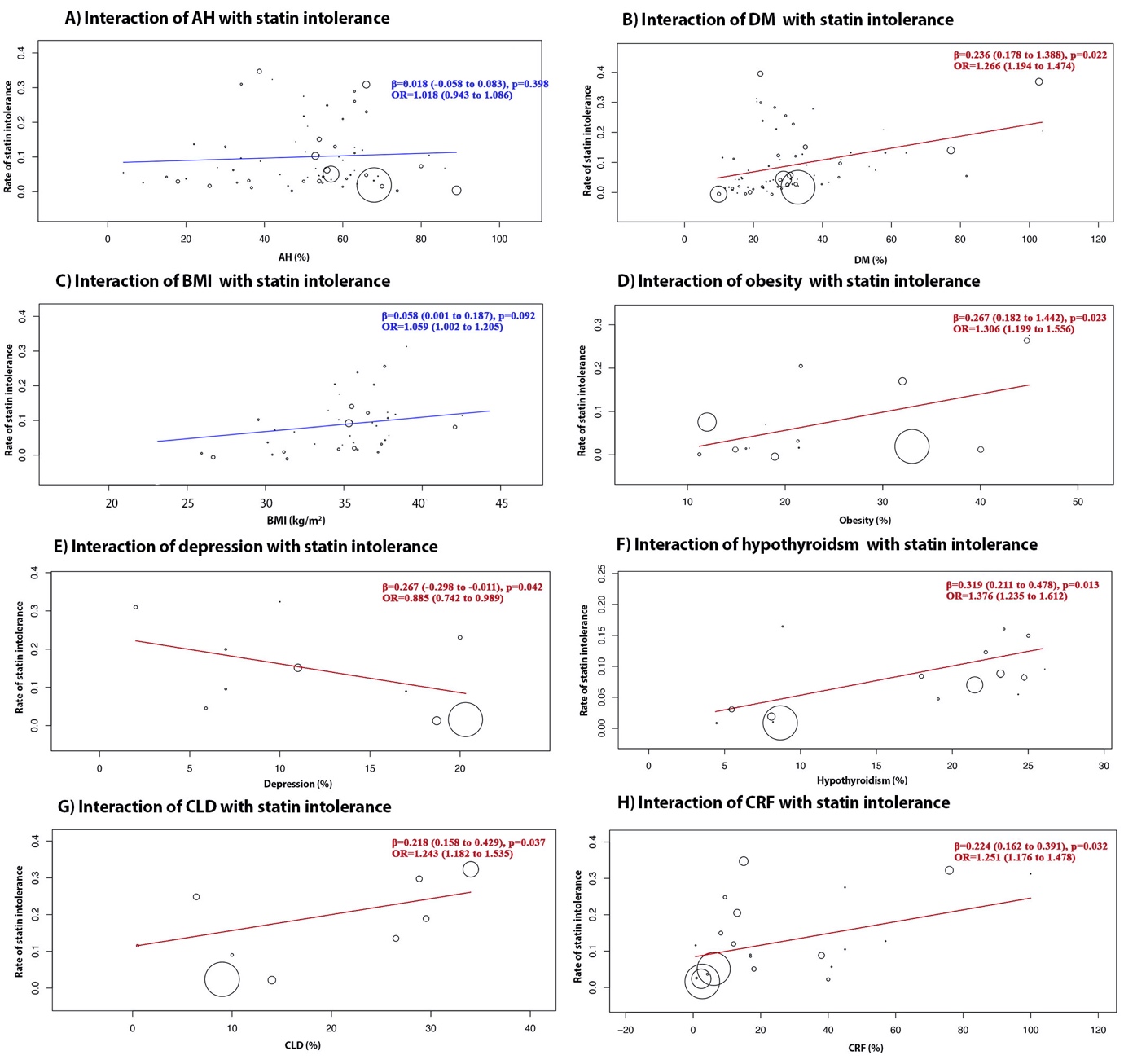


*Note:* D-L random-effects model used

**Figure S 14.** Meta-regression of risk factors and drugs on SI:

A)Smoking; B) Alcohol; C) Exercise; D) Warfarin; E) Calcium chanales blockers; F) Antyarrhytmic; G) Statin dose; H) Follow-up duration


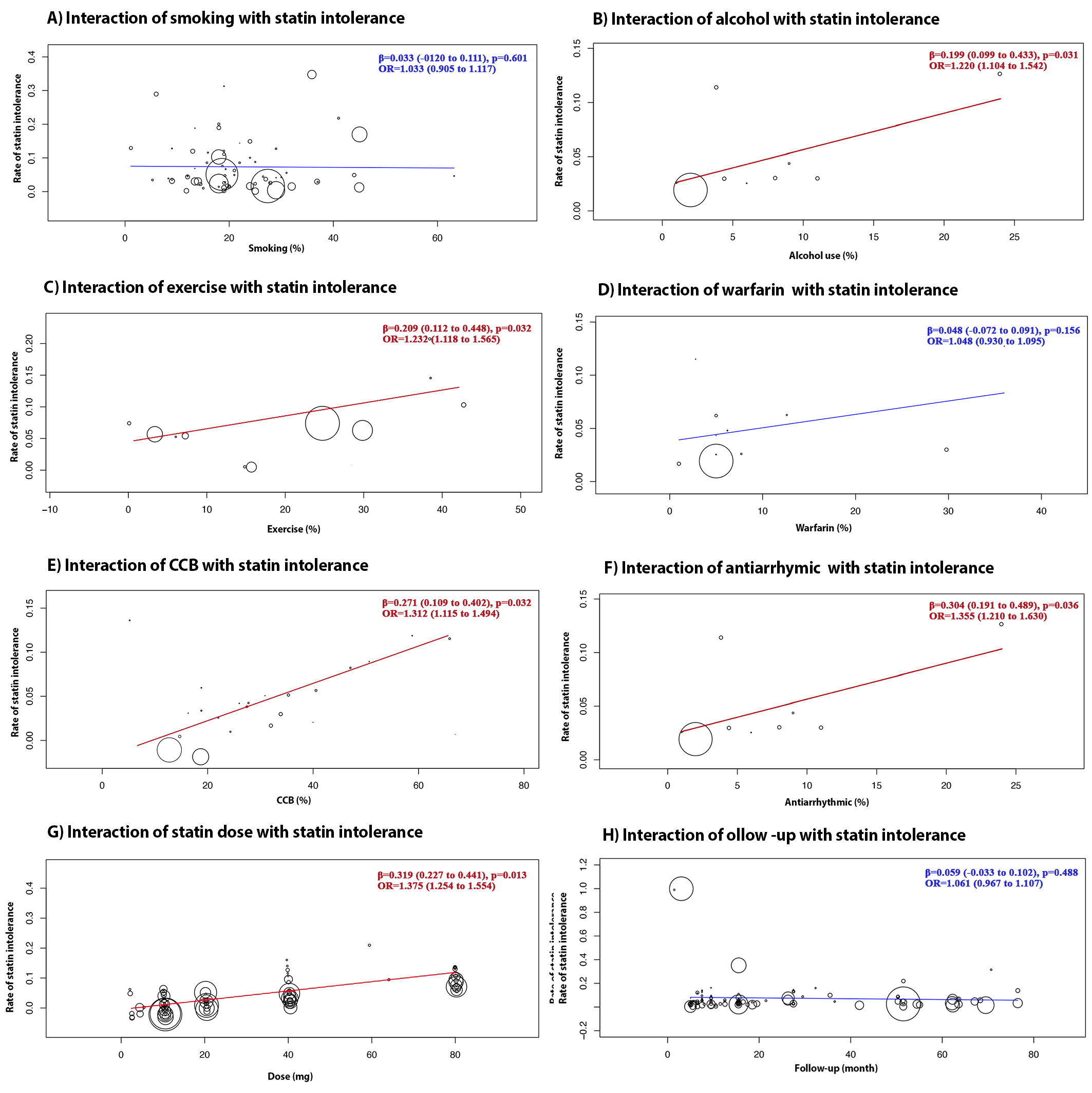


*Note:* D-L random-effects model used

**Table S4.** Assessment of risk of bias in the included studies using RoB2 for RCTs studies.

| **Study, year** | **Randomization** | **Deviation** | **Missing** | **Measurement** | **Selection** | **Overall** |
| --- | --- | --- | --- | --- | --- | --- |
|  | **process** | **from intended** | **outcome** | **of the** | **of the reported** |  |
|  |  | **interventions** | **data** | **outcome** | **results** |  |
| Bradford 1991 | L | L | L | L | L | L |
| Crepaldi 1991 | L | L | L | L | L | L |
| PMS group 1993 | L | L | L | L | L | L |
| PMS Group II 1993 | L | L | L | L | L | L |
| Blankenhorn 1993 | L | L | L | L | L | L |
| Wiklund 1993 | L | L | L | L | L | L |
| Furberg 1994 | L | L | L | L | S | S |
| Keech 1994 | L | L | L | L | L | L |
| Insull 1994 | L | L | L | L | L | L |
| Jacotot 1994 | L | L | L | L | L | L |
| Salonen 1994 | L | L | S | L | L | S |
| Shepherd 1995 | L | L | S | L | L | S |
| Pitt 1995 | L | L | L | L | L | L |
| Jukema 1995 | L | L | L | L | L | L |
| Sacks, 1996 | L | L | L | L | L | L |
| Bertolini, 1997 | L | L | L | L | L | L |
| Dart 1997 | L | L | L | L | L | L |
| Davidson 1997 | L | L | L | L | L | L |
| Herd 1997 | L | L | L | L | L | L |
| Jones 1998 | L | L | L | L | L | L |
| Tonkin 1998 | L | L | L | L | S | S |
| Downs 1998 | L | L | L | L | L | L |
| Eriksson 1998 | L | L | L | L | L | L |

*Continued table*

| Bruckert 1999 | S | L | L | L | S | **H** |
| --- | --- | --- | --- | --- | --- | --- |
| Serruys 1999 | L | L | L | L | L | L |
| März 1999 | L | L | L | L | L | L |
| Barter 2000 | S | L | L | L | L | S |
| Gentile 2000 | L | L | L | L | L | L |
| Schwartz 2001 | L | L | L | L | L | L |
| Olsson 2001 | L | L | S | L | L | S |
| Smilde 2001 | L | L | L | L | L | L |
| Andrews 2001 | L | L | S | L | L | S |
| Insull 2001 | L | L | L | L | L | L |
| Branchi 2001 | S | L | L | L | L | S |
| Illingworth 2001 | L | L | L | L | L | L |
| DALI study 2001 | L | L | L | L | L | L |
| Hunninghake 2001 | L | L | L | L | L | L |
| Saito 2002 | S | L | S | L | L | H |
| HPC Group 2002 | L | L | S | L | L | S |
| Shepherd 2002 | L | L | L | L | L | L |
| Serruys 2002 | L | L | L | L | L | L |
| Karalis 2002 | L | L | L | L | L | L |
| Mohler 2003 | L | L | L | L | L | L |
| Bruckert 2003 | L | L | L | L | L | L |
| Ballantyne 2003 | L | L | L | L | L | L |
| Kerzner 2003 | S | L | L | L | L | S |
| Rosenson 2003 | L | L | L | L | L | L |
| Holdaas 2003 | L | L | L | L | L | L |
| Olsson 2003 | L | L | L | L | L | L |

*Continued table*

| Ballantyne 2003 | L | L | L | L | L | L |
| --- | --- | --- | --- | --- | --- | --- |
| Schneck 2003 | L | L | L | L | L | L |
| Stein 2003 | L | L | L | L | L | L |
| Schuster 2004 | L | L | L | L | L | L |
| Schwartz 2004 | L | L | L | L | L | L |
| Beishuizen 2004 | S | S | L | L | L | H |
| Hunninghake 2004 | L | L | L | L | L | L |
| Goldberg 2004 | L | L | L | L | L | L |
| Cannon 2004 | L | L | S | L | L | S |
| Nissen 2004 | L | L | L | L | L | L |
| Koren 2004 | L | L | S | L | L | S |
| [Colhoun 2004](https://pubmed.ncbi.nlm.nih.gov/?term=Colhoun+HM&cauthor_id=15325833) | L | L | L | L | L | L |
| SSS Group | L | L | L | L | L | L |
| De Lemos 2004 | L | L | L | L | L | L |
| ADRAC 2004 | S | L | S | L | L | H |
| La Rosa 2005 | L | L | L | L | L | L |
| Pedersen 2005 | L | L | S | L | L | S |
| Amarenco 2006 | L | L | L | L | L | L |
| KNOPP 2006 | L | L | L | L | L | L |
| Nakamura 2006 | L | L | L | S | L | S |
| Nissen 2006 | L | L | L | L | L | L |
| Goldberg 2006 | L | L | L | L | L | L |
| Binbrek 2006 | L | L | L | L | L | L |
| Clearfield 2006 | L | L | L | L | L | L |
| Betteridge 2007 | L | L | L | L | L | L |
| Blagden 2007 | S | L | L | L | L | S |

*Continued table*

| Lee 2007 | L | L | L | L | L | L |
| --- | --- | --- | --- | --- | --- | --- |
| Kjekshus 2007 | L | L | L | L | L | L |
| Crouse JR 3rd 2007 | L | L | L | L | L | L |
| Leiter 2007 | L | L | L | L | L | L |
| Deedwinia 2007 | L | L | L | L | L | L |
| Tavazzi 2008 | L | L | L | L | L | L |
| Ridker 2008 | L | L | L | L | L | L |
| Newman 2008 | L | L | L | L | L | L |
| Bonnet 2008 | L | L | L | L | L | L |
| Conard 2008 | L | L | L | L | L | L |
| Abate 2008 | S | L | L | L | L | S |
| Yokote 2008 | L | L | L | L | L | L |
| Sasaki 2008 | L | L | L | L | L | L |
| Yamazaki 2009 | L | L | S | L | L | S |
| Budinski 2009 | L | L | L | L | L | L |
| Davidson 2009 | L | L | S | L | L | S |
| Insull 2009 | L | L | S | L | L | L |
| Fellström 2009 | L | L | L | L | L | L |
| Hall 2009 | L | L | L | L | L | L |
| Lablanche 2010 | L | L | L | S | L | S |
| Kim 2010 | L | L | L | L | L | L |
| Park 2010 | L | L | L | L | L | L |
| Armitage 2010 | L | L | L | L | L | L |
| Ose 2010 | S | L | S | L | L | H |
| Gumprecht 2011 | S | L | L | L | L | S |
| Albert 2011 | L | L | L | L | L | L |

*Continued table*

| Nicholls 2011 | L | L | L | L | L | L |
| --- | --- | --- | --- | --- | --- | --- |
| Baigent 2011 | L | L | L | L | L | L |
| Pitt 2012 | L | L | L | L | L | L |
| Nohara 2012 | L | L | L | L | L | L |
| Chen 2013 | L | L | L | L | L | L |
| Kim 2013 | L | L | L | L | L | L |
| Lee 2013 | L | L | L | S | L | S |
| Liu 2013 | L | L | L | L | L | L |
| Sasaki 2013 | L | L | S | L | L | S |
| Parker 2013 | L | L | L | L | L | L |
| Izawa 2015 | L | L | L | L | L | L |
| Yusuf 2016 | L | L | L | L | L | L |

***L:*** *Low;* ***S:*** *Some concerns;* ***H:*** *High*

| ***Overall risk-of-bias judgement*** | ***Criteria*** |
| --- | --- |
| *Low risk of bias* | *The study is judged to be at low risk of bias for all domains for this result.* |
| *Some concerns* | *The study is judged to raise some concerns in at least one domain for this result, but not to be at high risk of bias for any domain.* |
| *High risk of bias* | *The study is judged to be at high risk of bias in at least one domain for this result. The study is judged to have some concerns for multiple domains in a way that substantially lowers confidence in the result.* |

**Table S5.** Assessment of risk of bias in the included studies using Newcastle-Ottawa Quality Assessment Scale (NOS) for cohort (observational) studies.

| **Study, year** | **Selection** | **Selection** | **Selection** | **Selection** | **Comparability** | **Exposure** | **Exposure** | **Exposure** | **Quality** |
| --- | --- | --- | --- | --- | --- | --- | --- | --- | --- |
|  | **1** | **2** | **3** | **4** | **1** | **1** | **2** | **3** |  |
| Kannel 1990 | b) | a) | a) | a) | a) | b) | a) | a) | Good |
| Andrade 1995 | a) | b) | a) | a) | b) | b) | a) | c) | Good |
| Simons 1996 | b) | b) | c) | b) | b) | b) | b) | b) | Fair |
| Hiatt 1999 | a) | a) | b) | b) | a) | b) | a) | b) | Good |
| Jackevicius 2002 | b) | b) | a) | b) | a) | b) | a) | b) | Good |
| Benner 2002 | a) | b) | b) | b) | b) | a) | b) | b) | Good |
| Larsen 2002 | b) | b) | b) | c) | b) | b) | b) | c) | Fair |
| Wei 2002 | b) | b) | a) | b) | b) | b) | a) | b) | Good |
| Matsuzaki 2002 | a) | b) | a) | b) | b) | b) | a) | b) | Good |
| Yang 2003 | b) | b) | b) | b) | b) | b) | b) | c) | Fair |
| Abraha 2003 | b) | b) | b) | c) | b) | b) | b) | c) | Fair |
| Ellis 2004 | b) | b) | b) | b) | a) | b) | b) | b) | Good |
| Benner 2004 | b) | a) | b) | b) | b) | b) | b) | b) | Good |
| Eagle 2004 | a) | a) | b) | b) | b) | b) | b) | b) | Good |
| Howell 2004 | a) | b) | a) | a) | b) | b) | b) | a) | Good |
| Bruckert 2005 | a) | b) | a) | a) | a) | a) | b) | b) | Good |
| Caspard, 2005 | b) | b) | a) | b) | b) | b) | b) | b) | Good |
| Perreault 2005 | a) | b) | a) | b) | b) | a) | b) | b) | Good |
| Blackburn 2005 | b) | b) | a) | b) | b) | b) | b) | b) | Good |
| Save 2006 | a) | b) | a) | a) | a) | a) | a) | b) | Good |
| McGinnis 2007 | a) | b) | b) | a) | a) | a) | b) | b) | Good |
| Hudson 2007 | a) | b) | b) | b) | b) | b) | b) | b) | Good |
| Kamal-Bahl 2007 | b) | b) | b) | c) | b) | b) | b) | b) | Fair |

*Continued table*

| Yu 2008 | a) | b) | a) | a) | a) | b) | b) | b) | Good |
| --- | --- | --- | --- | --- | --- | --- | --- | --- | --- |
| Donnelly 2008 | b) | b) | a) | b) | b) | b) | a) | b) | Good |
| Chodick 2008 | b) | b) | b) | b) | b) | b) | b) | b) | Fair |
| Helin-Salmivaara 2008 | b) | a) | b) | b) | b) | a) | b) | b) | Good |
| Corrao 2010 | b) | a) | a) | a) | b) | b) | a) | b) | Good |
| Geers 2011 | b) | a) | b) | c) | b) | b) | b) | b) | Fair |
| Harris 2011 | a) | a) | b) | b) | b) | b) | a) | a) | Good |
| Cohen 2012 | a) | a) | b) | b) | b) | a) | b) | a) | Good |
| Esposti 2012 | a) | a) | b) | b) | a) | a) | a) | b) | Good |
| Zhang 2013 | b) | a) | a) | a) | a) | a) | a) | b) | Good |
| Rosenbaum 2013 | a) | b) | a) | b) | b) | b) | a) | b) | Good |
| Chang 2013 | a) | a) | b) | a) | a) | a) | a) | b) | Good |
| Mampuya 2013 | a) | a) | b) | b) | b) | a) | a) | b) | Good |
| Robison 2014 | a) | a) | a) | b) | a) | b) | a) | b) | Good |
| Ito 2014 | a) | a) | a) | a) | b) | a) | a) | b) | Good |
| Quek 2015 | b) | a) | a) | a) | a) | b) | a) | b) | Good |
| Svensson 2015 | b) | a) | a) | a) | a) | b) | a) | b) | Good |
| Vinogradova 2016 | b) | b) | a) | b) | b) | b) | b) | b) | Fair |
| Schulman 2016 | a) | a) | a) | b) | a) | b) | a) | a) | Good |
| Colantonio 2016 | a) | b) | a) | a) | a) | a) | b) | b) | Good |
| Halava 2016 | a) | b) | a) | a) | a) | a) | a) | b) | Good |
| Serban 2017 | a) | a) | b) | a) | b) | b) | a) | b) | Good |
| Brinton 2018 | a) | b) | a) | a) | a) | c) | a) | b) | Fair |
| Ihle 2018 | a) | a) | a) | b) | a) | b) | a) | b) | Good |
| Chee 2018 | a) | b) | a) | b) | a) | a) | a) | b) | Good |
| Nagar 2018 | b) | a) | a) | a) | b) | b) | a) | b) | Good |

*Continued table*

| van Delden 2018 | a) | b) | a) | a) | b) | b) | a) | b) | Good |
| --- | --- | --- | --- | --- | --- | --- | --- | --- | --- |
| Ofori-Asenso 2018 | b) | b) | b) | b) | b) | b) | b) | c) | Fair |
| Mefford 2018 | b) | a) | b) | a) | b) | c) | b) | b) | Fair |
| Kajinami 2019 | b) | a) | a) | a) | a) | b) | a) | b) | Good |
| Chen 2019 | a) | b) | a) | a) | a) | b) | a) | b) | Good |
| Ofori-Asenso 2019 | b) | a) | b) | b) | b) | b) | a) | b) | Good |
| Roh 2019 | a) | a) | a) | b) | a) | a) | a) | a) | Good |
| Bradley 2019 | a) | a) | b) | a) | a) | b) | a) | a) | Good |
| Jacobson 2019 | a) | a) | a) | b) | a) | a) | b) | b) | Good |
| Bair 2020 | a) | a) | b) | b) | a) | a) | b) | b) | Good |
| Casula 2020 | a) | a) | a) | b) | a) | a) | a) | b) | Good |
| Yao 2020 | a) | a) | a) | b) | a) | a) | a) | b) | Good |
| Thompson 2020 | a) | a) | b) | b) | b) | b) | a) | b) | Good |
| Moore 2020 | a) | a) | a) | b) | b) | a) | a) | b) | Good |

Legend: NOS: Selection- 1: a), b) one star, c), d) no star; Selection- 2: a) one star, b, c) no star; Selection- 3: a), b) one star, c), d), e) no star, Selection- 4: a) one star, b) no star; Comparability: a), b) one star, c) no star; Exposure-1: a),b) one star, c), d), e) no star; Exposure-2: a) one star, b) no star; Exposure-2**:** a),b) one star, c), d), no star.

**Good quality**: 3 or 4 stars in selection domain AND 1 or 2 stars in comparability domain AND 2 or 3 stars in outcome/exposure domain

**Fair quality:** 2 stars in selection domain AND 1 or 2 stars in comparability domain AND 2 or 3 stars in outcome/exposure domain

**Poor quality**: 0 or 1 star in selection domain OR 0 stars in comparability domain OR 0 or 1 stars in outcome/exposure domain
